# Projected declines in zooplankton energy supporting Northwest European Shelf ecosystems

**DOI:** 10.1038/s43247-026-03840-1

**Published:** 2026-07-23

**Authors:** Emma Tyldesley, Neil S. Banas, Sarah Wakelin, David G. Johns, Jennifer E. Jardine, Kate Searle, Francis Daunt, Colin Bull, Vladimir Krivtsov, Michael R. Heath, Douglas C. Speirs

**Affiliations:** 1 Department of Mathematics and Statistics, University of Strathclyde, Glasgow, UK; 2 Atlantic Salmon Trust, Orchard House, Kilgraston Walled Garden, Bridge of Earn, UK; 3 National Oceanography Centre Liverpool, Joseph Proudman Building, Liverpool, UK; 4 The Marine Biological Association, The Laboratory Citadel Hill, Plymouth, UK; 5 UK Centre for Ecology & Hydrology, Bush Estate Penicuik, Midlothian, UK; 6 Biological and Environmental Sciences, University of Stirling, Stirling, UK

**Keywords:** Ocean sciences, Marine biology, Biooceanography

## Abstract

The abundances of zooplankton species supporting Northeast Atlantic food webs have declined over the past 60 years, and their future is uncertain given continuing climate change. Here, we develop a new high-resolution machine-learning model of key taxa, driven by past ocean conditions and trained on Continuous Plankton Recorder observations. We apply it to an ensemble of regional climate projections for the Northwest European Shelf under the high emissions scenario RCP8.5 to enhance the detectability of a clear signal of climate-driven change in zooplankton abundance. The results project large declines in abundance: ensemble mean of 58%-72% by 2050 and 84%-93% by 2100 for small copepods and large *Calanus*. The projections are not geographically uniform, with evidence of regional refugia. The future declines are primarily driven by increasing temperature and decreasing salinity and nutrient concentrations, which shift conditions away from the species’ environmental niches and water mass associations. Impacts on food webs would be severe unless alternative zooplankton assemblages emerged to support existing planktivorous fish and their predators.

## Abstract (web summary)

Large declines in abundance of key zooplankton species on Northwest European Shelf can be driven by rising temperatures, falling nutrient concentrations, and a slowdown in the Atlantic Meridional Overturning Circulation, according to machine learning projections of a high emissions scenario.

## Introduction

Climate change is driving wide-ranging restructuring of global zooplankton assemblages^1,2^. General responses of zooplankton to warming oceans are range shifts, reduced body size and altered phenology^2^. Zooplankton species are moving polewards to maintain their temperature niches e.g^3–5^. Warming can reduce zooplankton community mean body size, either directly, through altered metabolic rates, or indirectly, through an environment that favours smaller species^6,7^. Warming of surface ocean layers increases water column stability, reducing nutrient supply and primary productivity. Altered seasonality of temperature and phytoplankton dynamics in turn affects zooplankton phenology and productivity^8,9^. In addition, climate change is altering ocean circulation, currents, winds and river run-off, all of which affect environmental conditions and dispersal patterns for zooplankton^10^. In combination, these pressures are resulting in altered zooplankton community compositions, size distributions, seasonality and abundance, e.g.^3,4,7,9,11–13^. These changes are highly regional^10^.

**Fig. 1:**
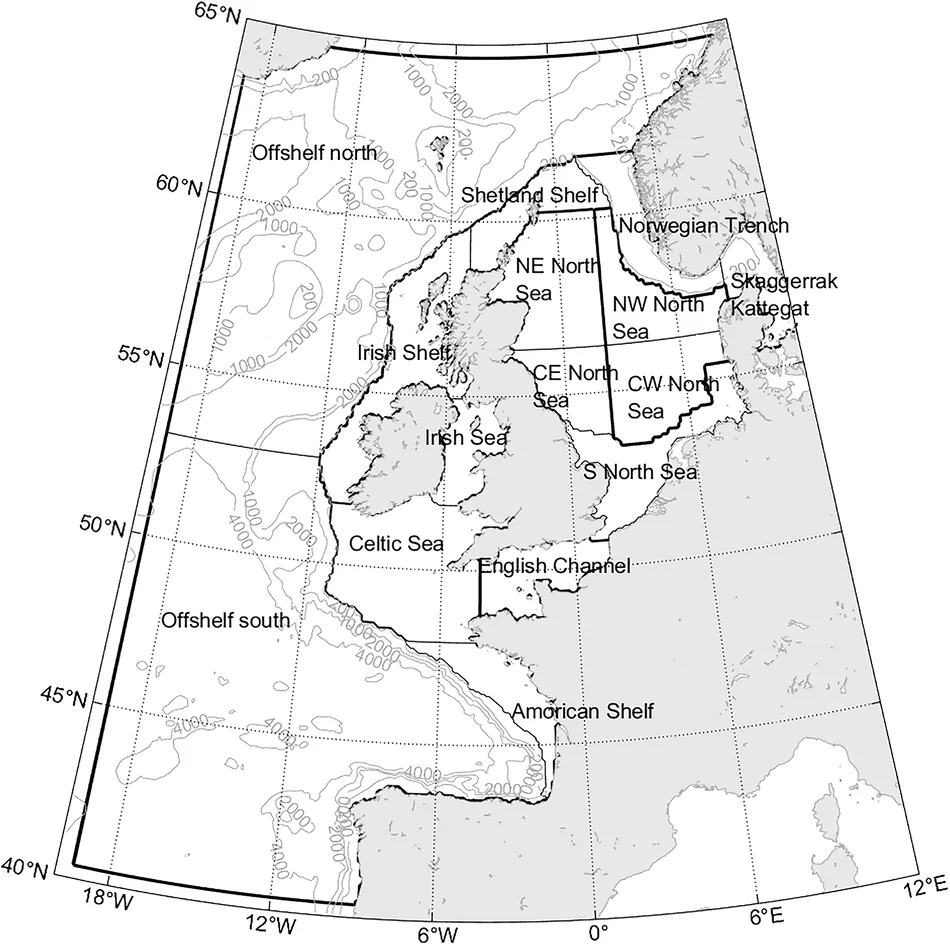
Study domain. Northwest European Shelf, averaging regions and bathymetry.

The aim of this study was to model future changes in the key zooplankton supporting food webs on the Northwest European Shelf, including the North Sea, the Irish Sea, the English Channel and the immediately adjacent waters (Fig. 1). This is a productive, biodiverse and economically important region that is subject to multiple and expanding anthropogenic stressors^14,15^. In the Northeast Atlantic, the dominant zooplankton group, copepods, is in a long-term abundance decline, which is expected to continue^16,17^. Crustacean zooplankton, especially copepods, are crucial in the efficient transfer of energy from phytoplankton to higher trophic levels such as fish, seabirds and mammals^18–20^. Evidence suggests that climate-driven changes in zooplankton are affecting higher predators^21–27^.

How ongoing climate change will affect zooplankton is highly uncertain. The current generation of global Coupled Model Intercomparison Project (CMIP) Earth System Models (ESMs) project declining zooplankton abundance^28^. However, accurate representation of zooplankton is not a focus of these models; the zooplankton terms are a large source of uncertainty that may be causing biases in climate projections^29^. An alternative approach to projecting zooplankton under climate change is to couple the physical and lower trophic level output of global climate models with zooplankton-focused statistical and mechanistic models^2,8,9,30,31^.

A global trait-based zooplankton model, driven by an ensemble of CMIP6 climate projections, showed 7–16% declines in global mean zooplankton by the end of the century under a range of emissions scenarios^1^. However, global projections probably underestimate some regional declines in zooplankton^1,28,32–34^ because of differences in how regions are responding to climate change and in how well they are represented by global ESMs. Coastal and shelf seas are poorly represented by global models because of coarse spatial resolution and the omission of coastal ocean processes^35^. Climate change impact studies for such areas, therefore, require a regional downscaling approach^36–41^. A regionally focused approach is particularly important for the Northwest European Shelf, which is warming faster than the global mean^42^. The region is also sensitive to another potential consequence of climate change: a slowdown in the Atlantic Meridional Overturning Circulation (AMOC), the system of currents that transports surface warm water north and deep cold water south within the Atlantic Ocean^43,44^. On the Northwest European Shelf, climate change is expected to result in warming, freshening, reduced circulation, reduced nutrients and increased water column stratification, but there is much uncertainty around the degree and trajectory of change^39,40^.

In this study, we generated high-resolution climate projections for the Northwest European Shelf (Fig. 1) for the key copepod zooplankton supporting planktivorous fishes and their predators. Models were developed for small copepod species modelled in aggregate, and the dominant large calanoid copepod species, *Calanus finmarchicus* and *Calanus helgolandicus*. The Calanus species were modelled separately because of their distinct spatial and ecological niches. Calanus zooplankton are lipid-rich, abundant zooplankton providing the key link between primary production and higher predators^45,46^. This is the main prey for many planktivorous fish, such as sandeel (*Ammodytes* spp.), sprat (*Sprattus sprattus*), herring (*Clupea harengus*), mackerel (*Scomber scrombrus*) and blue whiting (*Micromesistius poutassou*). *C. finmarchicus* is a boreal species dominating mesozooplankton biomass across the North Atlantic. In Arctic seas, it is replaced by the larger, polar congener species *C. glacialis* and *C. hyperboreus*^47^. *C. helgolandicus* is a temperate species occurring mainly on the continental shelf from the Mediterranean Sea to the North Sea^48^. Since the 1960s, the core range of *C. finmarchicus* has shifted north with warming oceans, and the core range of *C. helgolandicus* has expanded in all directions^49^. In the North Sea, *C. finmarchicus* abundance has declined, and *C. helgolandicus* has increased, so that it is now dominant where the two species overlap^49,50^.

Random Forest machine learning algorithms were used to model historical variability in copepod zooplankton abundance, derived from Continuous Plankton Recorder sampling, as a function of physical and biogeochemical predictors, derived from a regional ocean model reanalysis for 1998–2023. Predictors included in the models were yearday, bathymetric depth, daily sea surface temperature (SST), chlorophyll, mixed layer depth, sea surface salinity, monthly SST anomaly, upper ocean nitrate anomaly, annual winter nitrate concentration and an annual salinity-based proxy for the sub-polar gyre (SPG) strength.

We applied the zooplankton models to an ensemble of regionally downscaled, bias-corrected ocean model CMIP5 climate projections to 2100 under the Representative Concentration Pathways RCP8.5 emissions scenario (Table 1). This is a higher emission scenario than is currently considered plausible given developments in global climate policy and industry^51–53^. It is used here because the primary aim of the project is to understand the strength and direction of the regional response relative to global projections. The strong climate forcing gives a clear and interpretable signal of response in the zooplankton. The resulting zooplankton projections should be interpreted in terms of qualitative change and not as predictions of zooplankton biomass for a particular time. To account for inherent climate model uncertainty, we use a regionally downscaled ensemble whose parent global forcing models span the range of CMIP5 climate sensitivity^54^. Zooplankton projections were extracted for the near future (2040 s; +2.0 °C global surface temperature anomaly compared with pre-industrial conditions) and far future (2090 s; +3.7 °C)^43^. Under this high-emissions scenario, the model projects ensemble declines in copepod zooplankton abundance of 58–72% by 2050 and 84–93% by 2100. Although the magnitude of change is likely to be lower under more plausible emissions pathways, the results show the potential for larger declines on the Northwest Atlantic Shelf than predicted by global models, with likely negative impacts for higher trophic levels.

**Table 1:** Summary of model simulations used to derive past and future ocean conditions

Simulation	Time period	Horizontal grid	Vertical grid	Forcing data		Output temporal resolution	References
				Atmospheric	Ocean boundary		
Reanalysis	1993–2023	Atlantic Margin Model ~7 km resolution, 40° to 65°N, −20° to 13°E (AMM7)	Interpolated to 24 fixed depth layers.	ECMWF ERA5	GloSea5	Monthly and daily	^132,144,145^
ROAM-HADGEM2	1980–2099		33 terrain-following depth layers	HadGEM2-ES	NEMO-MEDUSA	Physics daily, biogeochemistry monthly	^37,41,148–150^
RECICLE-IPSL			51 terrain-following depth layers	IPSL-CM5A-MR		Monthly and daily	^147^
RECICLE-GFDL				GFDL-ESM2G		Monthly and daily	^146^

## Results and discussion

### Model structure and comparison with ecological knowledge

The trained random forest models explained 43%, 46% and 42% of the observed variability in abundance for small copepods, *C. finmarchicus* and *C. helgolandicus*, respectively. The unexplained variance may include missing predictors and ecological processes, measurement error and non-stationary processes. This is a typical level of model skill for statistical plankton spatial distribution models^55^. The explanatory power declined when testing the ability of the model to predict in areas of space or periods of time not included within the model training data using block cross-validation (Supplementary Information). Again, this is typical for statistical plankton models^55^. The unexplained variance will weaken the model’s ability to make precise forecasts; the zooplankton projections should instead be interpreted in terms of broad trends, differences due to the sensitivity of the climate models, species differences and relative spatial patterns.

**Fig. 2:**
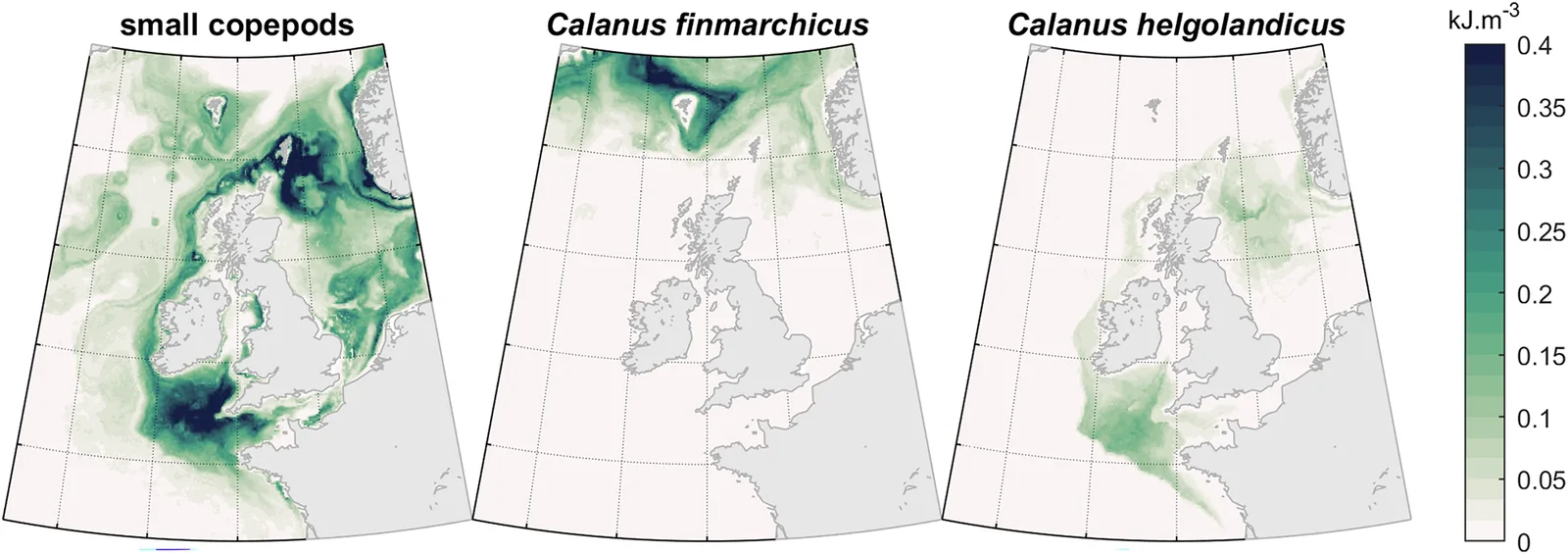
Modelled copepod zooplankton distributions. Modelled recent past distributions of aggregated small copepod species, *Calanus finmarchicus* and *Calanus helgolandicus*: mean over 2010–2022. Isobaths at 200 m and 800 m indicate the continental shelf edge.

The model predictions for present-day (mean over 2010–2020) spatial distributions of each zooplankton class matched existing knowledge, with the warm temperate species *C. helgolandicus* confined primarily to the shelf and shelf-edge, the sub-polar species *C. finmarchicus* occupying deeper, colder waters and some overlap in the northern North Sea (Fig. 2)^47,50,56,57^. It is harder to make a direct comparison for our small copepod class, but published distributions for each individual species indicate that in aggregate the abundance should be as predicted by our model: concentrated on the shelf, especially the North Sea and the Celtic Sea, and extending off-shelf, especially to the northwest^56^.

**Fig. 3:**
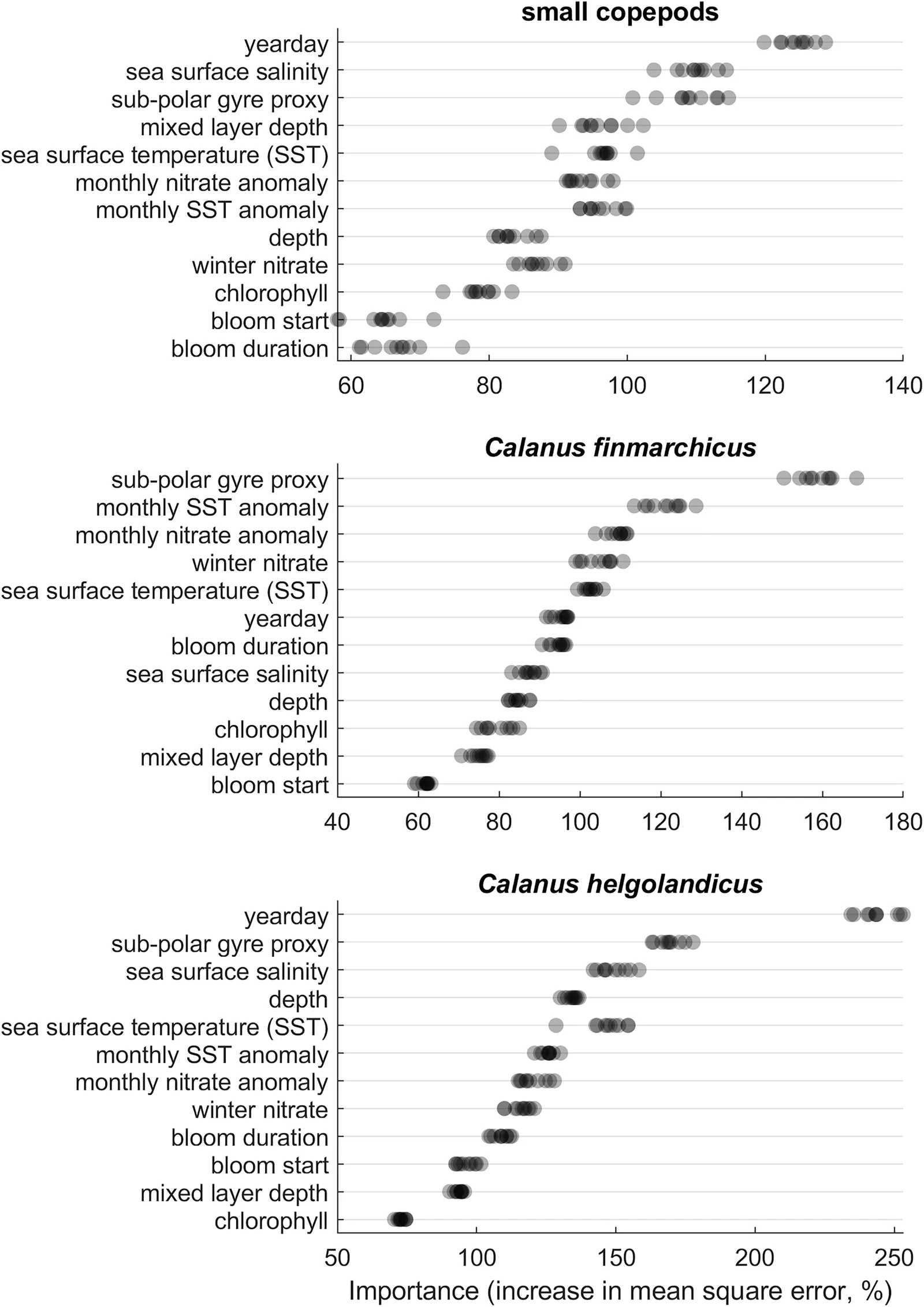
Environmental predictor importance. Importance ranking of predictors for each zooplankton class. Each point represents a repetition of training the models with the same parameters, but with random subsampling of data for each tree and predictors at each tree node.

Random Forest models calculate a measure of “variable importance”, i.e., how strongly each explanatory variable influences the model predictions. The importance ranking for each zooplankton group was stable, with only minor reordering of similarly ranked variables with each repetition of model training (Fig. 3). The strongest influence on the abundance of small copepods and the temperate shelf species *C. helgolandicus* was yearday, reflecting strong seasonality—zooplankton abundance in high latitude shelf seas is highly seasonal due to the strong variation in light, temperature, water column stratification and nutrient availability, which drives seasonal variation in phytoplankton, as well as directly affecting zooplankton metabolism^58^. For small copepods, the next strongest influences were salinity, temperature, subpolar gyre strength, mixed layer depth, SST anomaly and nitrate anomaly, each having a similar ranking. For *C. helgolandicus*, the SPG strength, SST, salinity and depth formed the next most influential group of predictors, reflecting the importance of water mass variability, seasonality and shelf habitat for this species. The most important predictor for *C. finmarchicus* abundance was the SPG strength, followed by SST anomaly. The next group of most influential predictors were nitrate anomaly, SST and winter nitrate.

**Fig. 4:**
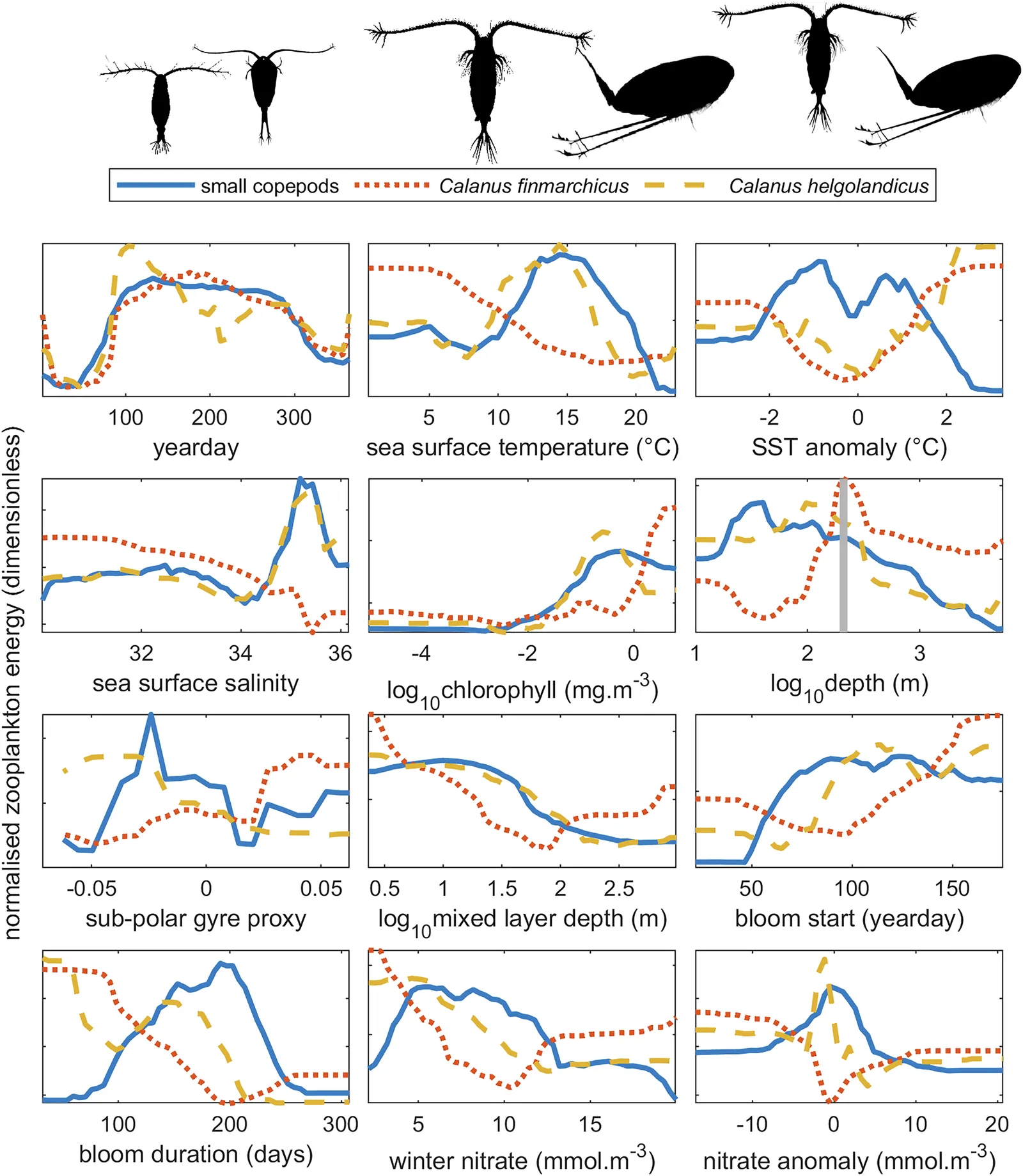
Influence of environmental predictors on zooplankton abundance. Partial dependence of normalised zooplankton energy on each environmental predictor. The vertical grey line in the depth plot indicates the continental shelf edge.

Figure 4 shows the marginal effect (partial dependency) of each variable on the predicted zooplankton abundance for each species. This shows how the variables have affected zooplankton abundance historically and, in combination with future projections of each variable, how zooplankton abundance may vary into the future. We can also compare the predicted relationships with existing ecological knowledge. These can sometimes be difficult to interpret if a variable has multiple, or spatially and temporally varying, impacts on zooplankton abundance. The partial dependencies indicate that the model successfully captures expected seasonal cycles, bathymetric, temperature and salinity niches and water mass associations of the different zooplankton classes. The model correctly predicts a steep rise in zooplankton abundance from April–May and a gradual decline over September-November^59^. *C. helgolandicus* peaks in both spring and autumn on the Northwest European Shelf, while *C. finmarchicus* and small copepods have one peak, the timing of which varies regionally^50,60^.

The model also captures the transition from small copepod and *C. helgolandicus* dominance on the continental shelf (<200 m water depth) to *C. finmarchicus* dominance in deeper water^57^. The model predicts peak *C. finmarchicus* abundance at 0–9 °C SST and below 35 salinity, and peak *C. helgolandicus* abundance at 13–17 °C and above 35 salinity, as observed^34,45,48,50,57^. As expected, a strong subpolar gyre was associated with increased *C. finmarchicus* and reduced *C. helgolandicus*, and vice versa^61,62^. The model also predicted a switch from small copepods and *C. helgolandicus* to *C. finmarchicus* dominance with increasing winter nitrate levels, as found previously^57^.

The model shows a saturating numerical (population) response with chlorophyll concentration, which is a proxy for the concentration of zooplankton’s phytoplankton prey. This threshold response is likely to result from the interplay of the zooplankton ingestion functional response, losses from metabolic costs and predation, and varying suitability of the phytoplankton community composition^63–65^. The increase in zooplankton abundance at mixed layer depth less than 100 m is in line with the increase in phytoplankton productivity that results from seasonal stratification^58^; the relationship with *C. finmarchicus* abundance appears to be more complicated and possibly reflects bathymetric influences or a mixed layer definition that is less appropriate off-shelf^66^.

The model results show that small copepod and *C. helgolandicus* abundance peaked when phytoplankton blooms started in April–May, whereas *C. finmarchicus* was more abundant for May–June blooms and decreased with longer bloom duration. This is probably related to the tendency for a later, shorter productive season with increasing latitude^67^, favouring taxa like *C. finmarchicus* that specialise in high energy storage and winter dormancy^30,45,50^.

The partial dependencies for winter nitrate and monthly anomaly in SST and nitrate are harder to interpret since they integrate the response of zooplankton abundance over season (month), time (the longer term climate signal), space and, in the case of the small copepods, also species. For example, a positive SST anomaly might lead to increased *C. finmarchicus* abundance in the northern regions of the model domain and decreased abundance in the North Sea. A warmer than usual spring might give a longer productive season and higher zooplankton abundance, or may be indicative of less productive water masses and lower zooplankton abundance. It is not straightforward to disentangle these signals. In general, the model predicts a bell-shaped curve of higher Calanus abundance at both low and high SST anomalies. For the aggregated small copepod group, small SST anomalies lead to increasing abundance while larger anomalies lead to a decline.

### Physical–biogeochemical climate projections

**Fig. 5:**
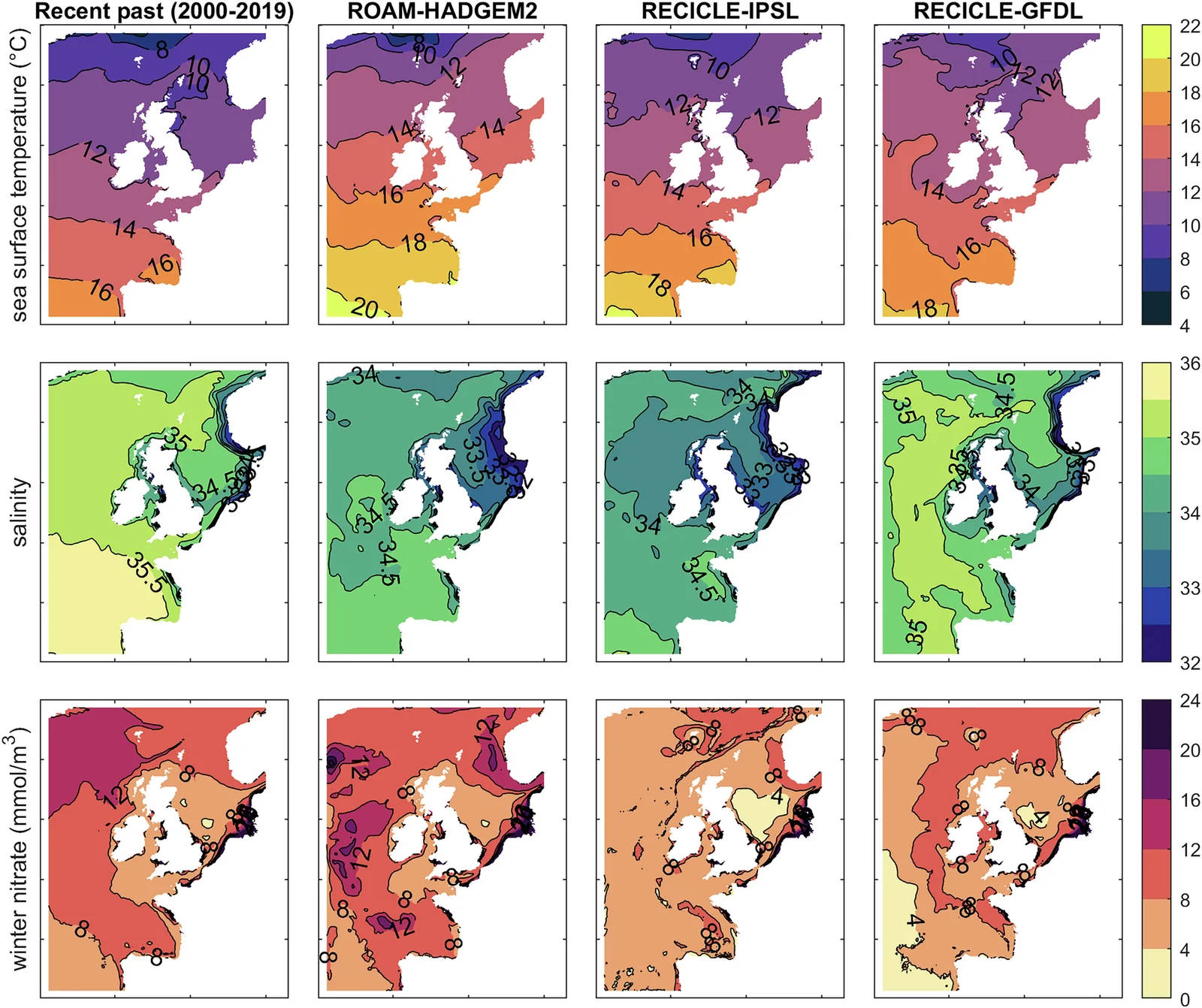
Far future marine conditions. Bias-corrected far future (2090–2100) mean decadal sea surface temperature, salinity and winter nitrate concentration for each climate projection model. The past ocean conditions are taken from the Northwest European Shelf model reanalysis provided by the Copernicus Marine Service. ROAM-HAGEM2, RECICLE-IPSL and RECICLE-GFDL are the regionally downscaled climate projection models that have been bias-corrected for this study. See Table 1.

The direction, magnitude and regionality of change of each of the predictor variables differ between the regional climate projections, despite all of them incorporating the same carbon emissions pathway (far future bias corrected SST, salinity and nutrients illustrated in Fig. 5; near future and far future change in all variables given in Supplementary Information Fig. S3). This is as expected from the range of physical projections made by their global forcing models^43^. A key difference among the global projections is the degree to which climate forcing is projected to weaken the AMOC, which affects water mass transport and circulation patterns on the Northwest European Shelf^43^. All three regional simulations used in this study project reduced Atlantic water salinity and circulation, seen as water mass freshening in all regions, but the change is minor for the GFDL projection (see Table 1 for details of the projection ensemble), dramatic but slow for HADGEM, and dramatic and rapid for IPSL (SI Fig. 3). The shutdown of exchange between the Atlantic and the North Sea and reduced circulation, salinity and nutrient concentrations in the North Sea has been described as a “novel potential future state for the North Sea”^37^.

As a result of the rapid weakening of circulation, IPSL projects cooling into the near future (by up to 0.4 °C by 2040–2050), while the other models project warming. All simulations project significant warming by the far future (2090–2100). All simulations project an increase in stratification (reduction in mean annual mixed layer depth) in all regions, as expected^40^.

The variation in physical projections affects the biogeochemical projections. Chlorophyll is projected to decrease in the near future, particularly for IPSL. By the far future, it decreases for all simulations in all regions except in the south off-shelf region for HADGEM. Both nitrate measures decrease into the near future and far future, except for HADGEM, which shows a far future increase for the shelf and south off-shelf regions. All simulations project a shift in phytoplankton bloom phenology: earlier blooms in most regions, longer blooms in the south off-shelf and shelf regions, and shorter blooms for the north off-shelf region.

### Zooplankton climate projections

**Fig. 6:**
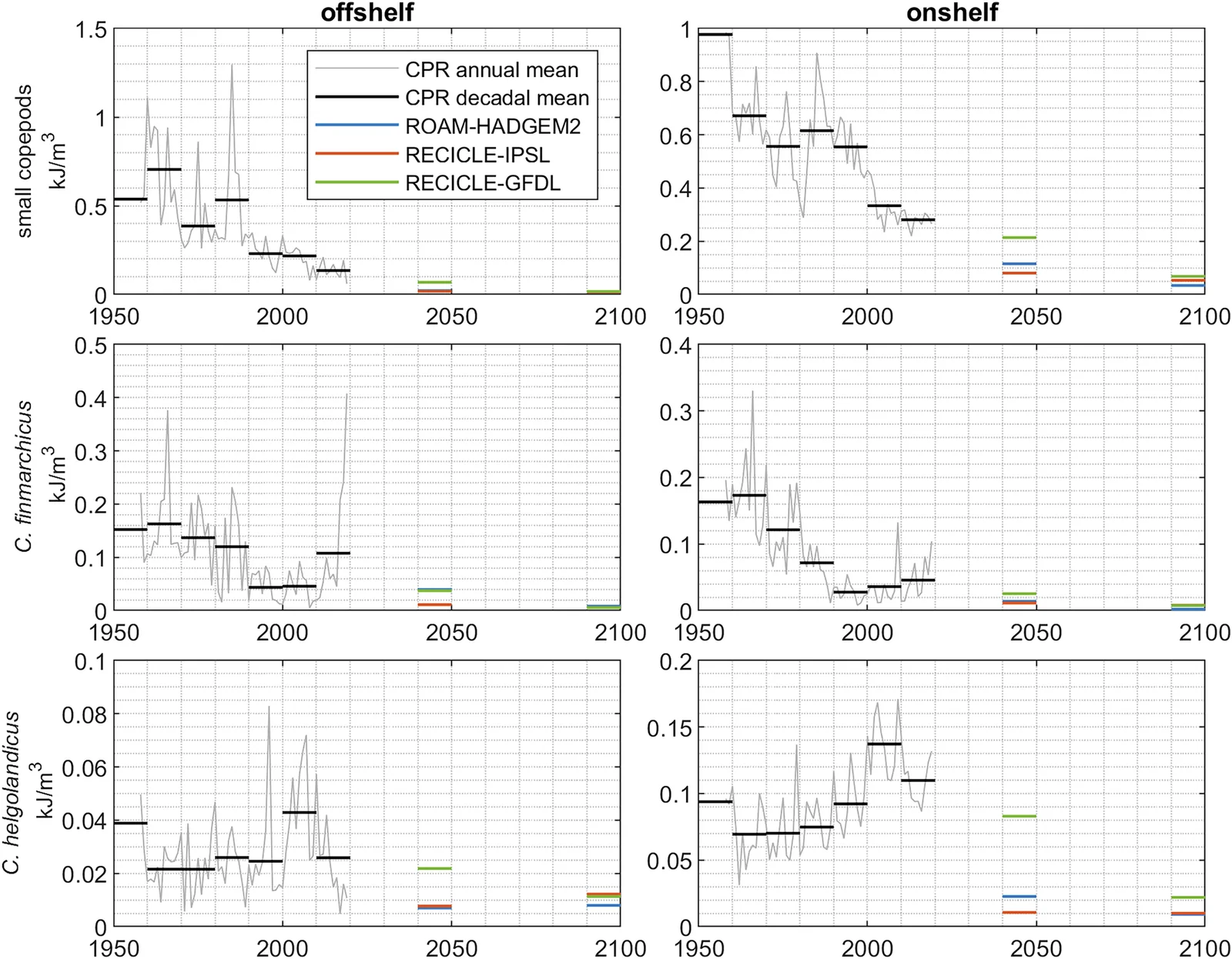
Past and future zooplankton abundance. Observed mean annual and decadal Continuous Plankton Recorder (CPR) zooplankton energy and modelled projected zooplankton energy for shelf (<200 m water depth) and off-shelf regions of the Northwest European Shelf.

**Fig. 7:**
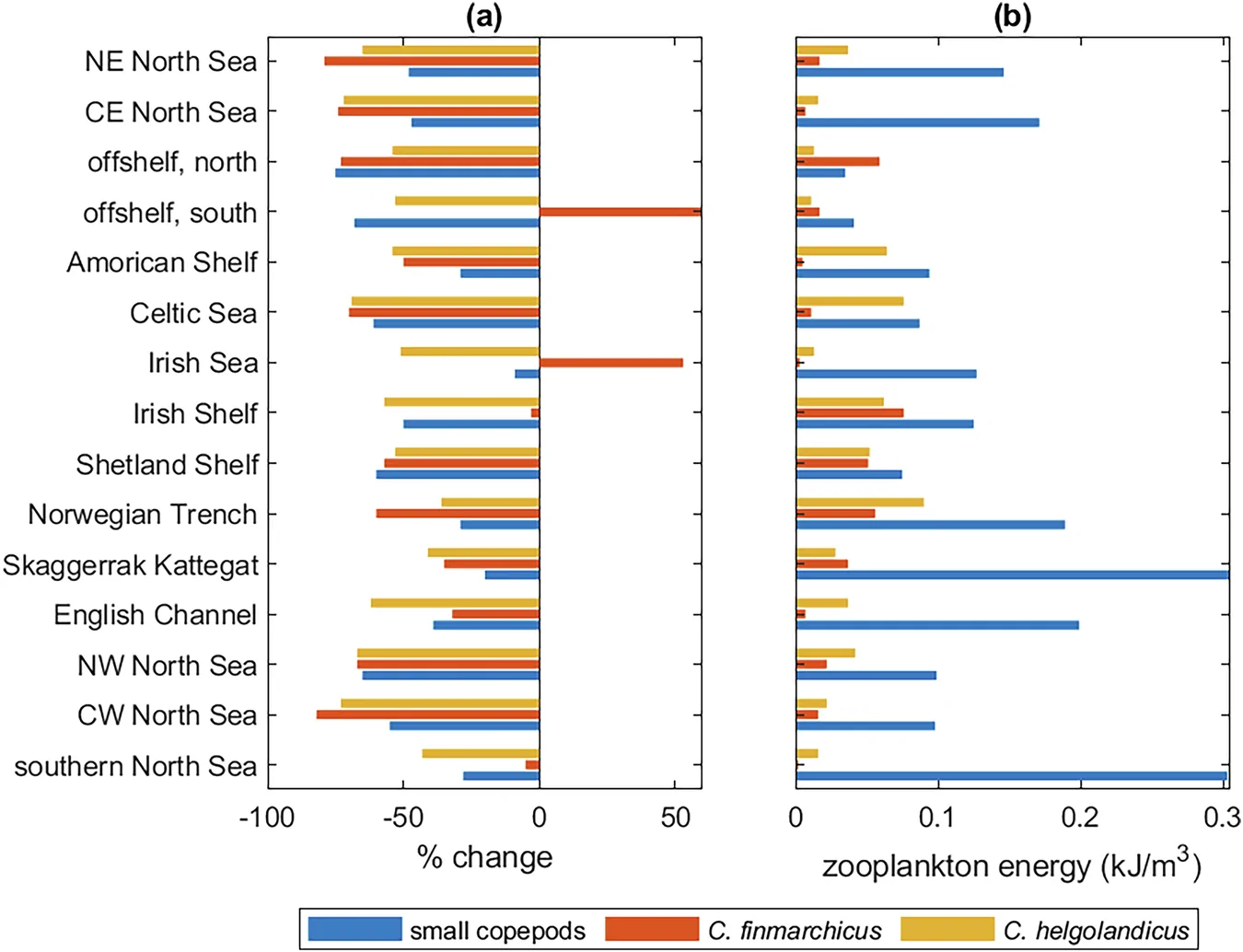
Regional projected zooplankton abundance. Projected near future (2040 s) ensemble mean **a** percentage change and **b** absolute zooplankton energy by taxa and Northwest European Shelf region. (Note that the *C. finmarchicus* increase in the offshelf south region (800%) extends beyond the upper limit of the percentage change axis, which is curtailed to allow interpretation for the other regions. The reason for this apparently large relative change is that historical *C. finmarchicus* abundance in this region is negligible, since the species is associated with subarctic water masses further north (see Fig. S4). Declining future salinity in that region causes the model to project a small absolute increase in *C. finmarchicus* abundance, which results numerically in a large percentage change even though the resulting projected densities are still low in comparison with historical densities in the core regions for this species (see panel **b** and Fig. S4).

The varying physical–biogeochemical climate projections combined with the differential influence of each environmental predictor on zooplankton density translated into a range of zooplankton climate projections (Figs. 6 and 7 and Table 2). However, zooplankton density was projected to decline for all zooplankton classes over most of their current range. The ensemble mean decline averaged over the Northwest European Shelf was 58% for small copepods, 72% for *C. finmarchicus* and 64% for *C. helgolandicus* by the near future, and 84% for small copepods, 93% for *C. finmarchicus* and 84% for *C. helgolandicus* by the far future.

**Table 2:** Projected percentage change from the recent past by zooplankton class and climate projection model. Mean is the climate projection ensemble mean

Zooplankton class	Region	Near future (2040–2050)				Far future (2090–2100)			
		HADGEM	IPSL	GFDL	Mean	HADGEM	IPSL	GFDL	Mean
Small copepods	Off-shelf	−84	−87	−50	−74	−90	−90	−87	−89
	Shelf	−60	−72	−23	−52	−88	−81	−76	−82
	Full domain	−67	−76	−31	−58	−89	−84	−79	−84
*C. finmarchicus*	Off-shelf	−63	−90	−66	−73	−92	−95	−95	−94
	Shelf	−70	−75	−44	−63	−96	−83	−84	−87
	Full domain	−64	−88	−63	−72	−93	−93	−94	−93
*C. helgolandicus*	Off-shelf	−70	−68	−16	−51	−69	−47	−50	−56
	Shelf	−80	−90	−23	−64	−92	−90	−79	−87
	Full domain	−79	−88	−23	−63	−89	−86	−77	−84

Into the near future, the rate and magnitude of decline varied by physical–biogeochemical climate projection, zooplankton class and region (Figs. 6 and 7 and Table 2). The GFDL projection gave the weakest declines in zooplankton density; HADGEM and especially IPSL resulted in stronger declines. For example, GFDL projected a decline in small copepod density of 24% on-shelf and 49% off-shelf by the near future, while the HADGEM (59% on-shelf and 84% off-shelf) and IPSL (71% on-shelf and 87% off-shelf) projections were in closer agreement with one another. In contrast, IPSL projected a 90% decline in near-future off-shelf *C. finmarchicus* abundance, while GFDL and HADGEM gave similar declines of 65% and 63%, respectively. Although seemingly stark, Continuous Plankton Recorder (CPR) observations suggest these projected declines are comparable with recent trends for small copepods and *C. finmarchicus* (Fig. 6).

By the far future, there were major declines in zooplankton density for all models for most areas. The projections were similar between models for small copepod (declines of 88% to 91% off-shelf and 76% to 88% on-shelf) and *C. finmarchicus* (declines of 92% to 95% off-shelf and 83% to 96% on-shelf) density, but more divergent for *C. helgolandicus* (declines of 53% to 69% off-shelf and 80% to 92% on-shelf). Overall, the HADGEM projection gave the most pessimistic results by far in the future.

There was no consistent pattern in whether declines were highest on or off the shelf. Overall, *C. finmarchicus* had the largest projected declines out of the modelled zooplankton groups. The HADGEM and IPSL models indicated a timing shift in peak small copepod and *C. helgolandicus*, but not *C. finmarchicus*, abundance from spring to autumn; otherwise, there were no obvious changes in the zooplankton seasonality at the monthly scale analysed here (Fig. S5).

At the regional scale (Fig. 1), there were projections that diverged from the shelf/off-shelf scale trends (Fig. 7). There were regions such as the English Channel, the southern North Sea, the Celtic Sea, the Irish Sea and the Norwegian Trench where declines in zooplankton were less severe for some or all modelled species. In addition, all models projected increases in *C. finmarchicus* density in the southern half of the off-shelf region. However, the resulting densities were still low in comparison with historical densities within the core range for this species. Species distribution modelling typically has lower skill in areas outside the main distribution of a species, where prevalence is low and historical observations are highly variable^55^. This divergence of trends suggests the potential for regional refugia where zooplankton density may be sufficiently maintained to support existing food chains. However, this does not change the overall projection of large declines in copepod abundance across most of the Northwest European Shelf.

### Summary of results

The machine learning models reproduced expected spatial distributions and relationships with predictor variables for each zooplankton group and explained approximately 45% of the variability in historical abundance. The strongest predictors were yearday, surface salinity and temperature, nitrate concentration and prevailing water mass. In this study, the declines in copepod zooplankton were driven by the projected changes in temperature, salinity and nutrient concentrations (Fig. 5). Temperatures increased above the preferred range for each zooplankton group, leading to lower projected densities in previous core areas. Salinity was projected to decline to levels below those of the Atlantic and mixed Atlantic-shelf waters historically associated with high densities of *C. helgolandicus* and small copepods, whilst favouring *C. finmarchicus*. Nutrient concentrations declined to values historically associated with low zooplankton density, presumably because these broadly indicate low primary productivity and depressed food availability for zooplankton, i.e. bottom-up control. For *C. finmarchicus*, the primary driver was temperature: although projected salinities were within the preferred range, future warming moved the temperature contours north until by the far future the area within its optimal range (<9 °C) had shrunk to a small region north of the Faroe Islands, effectively excluding the species from the model domain. For *C. helgolandicus* and small copepods, the primary driver was freshening of water masses and reduced circulation: the salinity contours shifted south and out of the North Sea so that only the southern-most part of the domain was within the preferred salinity range (<35).

In summary, a high-emissions scenario resulted in large declines in abundance for all copepod zooplankton groups and all climate models. *C. finmarchicus* was the worst-affected taxon. HADGEM gave the largest declines. This was the model with the largest future reduction in Atlantic circulation, resulting in a North Sea with reduced oceanic input, slowed circulation and increasingly estuarine conditions, as seen in several regional climate projections^37,39^. The model with the least change in circulation and sea surface salinity, GFDL, resulted in the smallest zooplankton declines, as would be expected.

There was regional variation in projected zooplankton abundance, with the smallest declines in the English Channel, the southern North Sea, the Celtic Sea, the Irish Sea and the Norwegian Trench. These are the areas with the lowest connection to the Atlantic Ocean inflows to the North Sea, where environmental conditions are least affected by projected changes in ocean water mass properties and circulation^40^. They also coincide with the areas that have not had recent declines in copepod zooplankton^17^.

### Comparison with other projections

The zooplankton declines in this study are large in comparison with global projections. Heneghan^1^ reported a 7% to 16% decline in total zooplankton by 2080–2100 under a range of emissions scenarios and models. For their zooplankton group and emissions scenario closest to those used in this study (omnivorous zooplankton under SSP5–8.5), global declines were around 14–22%, and the mapped results indicate that declines on the Northwest European Shelf were larger than elsewhere. CMIP6 ESMs project global change in (predominantly copepod) zooplankton of approximately +5 to −18% by 2051–2100 under RCP8.5^28^. However, these projections show large regional variation, and the mean change for the Northwest European Shelf varied from −7% to −64% by 2051–2100 (data supplied by C. M. Petrik; analysis presented here in Supplementary Information). Clearly, there is the possibility for changes in zooplankton biomass to be much larger on the Northwest European Shelf than at the global scale, due to temperatures rising faster than the global mean^42^ and the particular sensitivity of this region to a reduced AMOC^44,68,69^.

There are limited regional studies with which to compare our results. In general, North Atlantic copepod zooplankton distributions have shifted north with warming seas as species track their preferred temperature ranges, e.g.^2,3^. Theory and modelling suggest zooplankton range shifts are likely to continue with climate change^70–72^. However, habitat availability and physical barriers such as currents may limit a species’ ability to adjust its distribution, leading to eventual range squeeze and population decline, e.g. as observed for krill in the North Atlantic^4^. Environmental niche modelling shows *C. finmarchicus* occurring with such low probability as to be “functionally extirpated” (i.e. the population is so depleted that it no longer plays a significant role in the ecosystem, even if individuals still exist) in Northwest European Shelf seas by the end of the century as a result of changes in SST alone^34^. In the Northeast Atlantic, *C. finmarchicus* is expected to expand north and west into the Nordic Seas and decline in the North Sea, the southern Norwegian Sea and southern regions^71^. On the other side of the Atlantic, the northeast United States continental shelf *C. finmarchicus* abundance has been projected to decline by 50% by 2100 under RCP8.5^33^. Since the 1960s, warming seas have caused *C. helgolandicus* to expand north and replace *C. finmarchicus* in the North Sea^27,49^. Future expansion depends on whether *C. helgolandicus* is limited to the continental shelf^50^. This species typically overwinters in the relatively shallow shelf seas, which remain favourable for survival throughout the year, rather than entering diapause (dormancy) as required by oceanic species. Nutrient-poor open ocean environments lack this overwintering habitat. Further, reduced circulation between the North Sea and Atlantic Ocean may restrict dispersal of *C. helgolandicus* into potential new ocean habitats, even if surface water temperatures are within the preferred range. If *C. helgolandicus* is restricted to shelf seas, then ongoing climate change would eventually lead to range contraction and abundance decline.

### How likely is this future?

Assessing the plausibility of these projected declines in copepod zooplankton can be divided into several questions: how likely is the projected physical–biogeochemical future, how likely is the projected zooplankton response to this future environment, and how much uncertainty is inherent in the machine learning modelling approach?

The overall physical projections for the Northwest European Shelf, of warming, freshening, reduced circulation and increased water column stratification, are in line with global and regional projections^39,40,43,73^. The ensemble spread is within the range of other projections for this region, but both IPSL and HADGEM have circulation and salinity changes towards the high end of projected response; the projected zooplankton declines are also therefore expected to be at the high end of what would be seen for a broader ensemble of climate projections. The biogeochemical projections are consistent with this physical response and observations of recent trends^15,37,41,74^.

The RCP8.5 represents a continued, unmitigated rise in emissions over the century and, as such, is now considered an overly pessimistic driving scenario for climate change impact assessment, with higher emissions than the “high end” scenario in CMIP7^51–53^. It is, therefore, important that the projected declines in zooplankton are interpreted in process terms, and in comparison with global declines, rather than as quantitative values for specific time periods. RCP8.5 is used here because the strong forcing gives a clear physical and biological response, which results in an interpretable signal of change in the zooplankton^40^. Our study is intended to illustrate the trajectory, rather than the exact magnitude, of change^75^. No regionally downscaled projections were available for a more moderate emissions pathway. Under more moderate emissions scenarios, the rate of decline may be slower than projected here; for example, under RCP4.5, the near-future physical changes projected by RCP8.5 would be delayed until the end of the century^43^. The use of RCP8.5 gives valuable information on a high-impact, low likelihood ecological response that may yet be plausible under future climate policies and uncertain climate feedbacks or tipping points^76^. Because other emissions scenarios project a similar direction of physical change, it is likely that the direction of change in copepod zooplankton abundance is robust even if the magnitude and speed are pessimistic.

Note that no regionally downscaled projections were available for the more recent CMIP6 models. This means that the zooplankton projections here are based on the CMIP5 RCP8.5 rather than the CMIP6 Shared Socioeconomic Pathway SSP5–8.5. RCP8.5 and SSP5–8.5 have similar levels of total radiative forcing, but CMIP6 models have stronger climate sensitivity, projecting stronger warming than CMIP5 models^77,78^. Therefore, the projected zooplankton changes under RCP8.5 are likely to be a lower bound on SSP5–8.5.

Given this physical–biogeochemical future, the modelled zooplankton projections make ecological sense if the species continue to track their historical environmental niches. The projected declines are in line with long-term and recent trends in observed copepod zooplankton abundance and range shifts^3,17,21,25,27,79^ and modelling studies (see above). Extrapolation of statistical spatiotemporal distribution models in space, time and environmental predictor space, as necessitated by climate change impact studies, is challenging^55,80–82^. Projections may be less certain if the relationships between zooplankton abundance and environmental predictors fail to persist. This could happen for several reasons.

First, the changing environment may force zooplankton adaptation and new biotic interactions. Thus far, zooplankton populations appear to have responded to warming oceans by shifting their timing and distributions to remain within their environmental niches^5,8,34,47,49^. However, organisms can also cope with rapid environmental change through phenotypic plasticity (acclimation through shifts in body physiology or morphology within the lifetime of the organism) or evolution (adaptation through genetic selection of traits over several generations). This can buffer populations from declines under environmental stress. Both plasticity, e.g.^83^. and adaptation, e.g.^84–86^ have been demonstrated in copepod zooplankton, leading to rapid but incomplete recovery of population fitness under environmental stress. The extent of recovery can be limited by fitness trade-offs, especially under the action of multiple environmental stressors, as is the case with climate change^85,87^. By not explicitly representing adaptive mechanisms, which would be computationally expensive and difficult to parameterise due to a lack of data, the model may be overestimating the copepod population declines to some extent.

Second, relationships captured by the model may be associative rather than functional^80^. To address this, we selected environmental variables likely to have a functional relationship with zooplankton abundance, avoided proxy variables such as latitude and longitude, confronted the model with existing ecological knowledge and checked for stability of the model structure across the time span of the data. However, the model fit did decline with temporal cross-validation. By the near future, small areas of the domain (Bay of Biscay, Norwegian Trench and parts of the central and northern North Sea) shifted into novel environmental predictor space and by the far future, increasing SST took the entire domain into novel predictor space (Supplementary Information). Therefore, the far future projections should be considered particularly uncertain.

### Other caveats

In this study, we make the assumption of predominantly bottom-up control of copepod zooplankton, justified given that zooplankton track environmental variation, especially over long time scales, e.g.^9,62^. This will not always be the case—for example, larval fish exert significant predation pressure on zooplankton^18,88^, but observations of larval fish abundance are not available at sufficient resolution to include within our study framework. We do not explicitly represent any species interactions, such as competition or predation by other copepod zooplankton. We neglect any species-specific responses to climate change within the aggregated “small copepod” group; disaggregating this group would significantly reduce the number of observations available for model fitting.

The model region, the Northwest European Shelf, does not include the full main distribution of *C. finmarchicus*, which extends north and west into the Northeast Atlantic^47^. It is not possible to expand this study to include the more northerly distribution because the regional projections have a northern boundary of 64°N, and CPR coverage in the Norwegian Sea is extremely limited spatially and temporally, e.g.^27^. This means that from our study, we cannot infer the future *C. finmarchicus* distribution over the full Northeast Atlantic and whether, for example, the species will shift north into Arctic waters currently dominated by *C. glacialis* and *C. hyperboreus*. However, this does not affect the conclusions of the present study, which shows that even the most northerly regions of the Northwest European Shelf domain may become unsuitable for *C. finmarchicus* under future climate change.

### Food web consequences of projected declines

Copepod zooplankton are a key component of the diet of many planktivorous fish, including adults or larval stages of commercially important species, e.g. blue whiting, herring and sandeel (*Ammodytes marinus*)^25,89–91^. Variation in copepod zooplankton abundance has been linked with fish recruitment, abundance and marine survival^25,27,92–95^. Modelling studies suggest that reduced copepod zooplankton abundance results in a lower “carrying capacity” for planktivorous fish production^9,28^. In turn, reduced forage fish abundance has been negatively associated with diet composition, survival, growth and breeding success of higher predators such as seabirds^70,96–100^, Atlantic salmon (*Salmo salar*)^27,101–104^ and other predatory fish^105^, and marine mammals^106,107^. A major climate-driven decline in copepod zooplankton could therefore have negative consequences for forage fish, higher predators and stocks of commercial fisheries.

Our study focused on copepod zooplankton, which form one, albeit sizeable and carbon-rich, component of forage fish diet. Other zooplankton observed in forage fish diets include euphausiids, amphipods, polychaetes and Appendicularia (larvaceans)^25,108–110^. Some of these have also declined in abundance over the past decades, while others have opposite or unknown trends^4,11,16,27^. Climate change is expected to have differential impacts on different zooplankton taxa, e.g.^12,110^. The wider ecological impact of a restructured zooplankton assemblage resulting from climate change would depend on whether the prevailing species could support existing food webs, in terms of energy transfer and predator foraging adaptability. For example, climate change is expected to favour Appendicularia, a group of gelatinous zooplankton, which is important prey for many fish species^12,111^; Appendicularia have fast growth rates and a carbon content similar to copepods, so they have the potential to buffer existing food webs from declines in the currently dominant zooplankton^110^. However, Appendicularia may not be a suitable alternative prey: they are patchily distributed, less lipid-rich and harder to handle than copepods, and their high predator to prey size feeding ratio and rapid turnover of mucus feeding “houses” can lead to high carbon export instead of efficient transfer of energy to fish and higher trophic levels^7,111^.

## Conclusions

This study shows the potential for declines in copepod zooplankton abundance on the Northwest European Shelf with future climate change that exceed those of global projections under an extreme climate-forcing scenario. Such declines could reduce the robustness of ecosystems supporting higher predators, such as seabirds, predatory fish and cetaceans, as well as many commercial fisheries. Against the backdrop of this “shifting baseline”, this area is subject to expanding anthropogenic pressures (e.g. increasing offshore wind, BEIS, 2022). Increasingly, there is a need to ensure that marine spatial planning and consenting are resilient to climate change^98,112^. The results of this study imply that environmental impact assessment and conservation measures will need to be robust to the potential negative effects of declining zooplankton on the wider Northwest European Shelf ecosystem.

## Methods

Analysis was carried out using R^113^, implemented through R Studio^114^, and the MATLAB numerical computing environment^115^. Maps were generated using the MATLAB packages *cmocean*^116^ and *M_Map*^117^.

### Model building

Predictive models of zooplankton variability were generated here using Random Forest (RF) ensemble machine learning^118^. RF generates predictions by aggregating a “forest” of decision trees, each trained on a random subset of the data and using a random subset of the explanatory variables (hereafter, variables) at each tree node. RF is non-parametric, i.e. no assumption is made about the shape of the relationship between variables and response. RF is widely used because it has high predictive power, flexibility to data types, avoids overfitting and provides a ranking of variable importance^119^. Variable importance is calculated as the mean increase in predictive error resulting from the random permutation of each variable. Model prediction error is calculated as the mean out-of-bag error, i.e. predicting on observations outside the training subset for each tree.

Modelling was conducted using the R package *randomForest*^113,120^. Projecting distributions of plankton, which are patchily distributed and interact with a dynamic and changing physical and biological environment, has been shown to be inherently challenging^55,121^. With the aim of gaining ecological insight and building models with high predictive capacity, variables were selected that were likely to be proximal, functional influences on zooplankton variability^80^. Latitude and longitude were excluded because strongly geographical variables can result in a model with low error but poor predictive capacity for unsampled areas^81^. Although a proxy variable, yearday was included in the model to represent internally and externally driven copepod phenology not governed by the available seasonally varying predictors, such as the dynamics of diapause strategy and the timing of arrival of *C. finmarchicus* advected into the study region^122^. Including yearday allows the model to capture the response of zooplankton to both changing seasonality (e.g. warmer springs) and fixed seasonality (e.g. light availability) under future climate change. Variables were checked for pairwise collinearity using a threshold of Pearson’s correlation of 0.7 prior to inclusion in model fitting.

The RF method of averaging over many predictions tends to produce systematic bias. This manifests as a sum of errors near to zero but with high response values underestimated and low values overestimated, i.e. the slope of predicted on observed values is less than one^123^. In our case, this could give, for example, overestimated winter zooplankton abundance and reduced amplitude of spring blooms. We corrected for this by fitting a standard linear regression of observations on predictions and using the coefficients to calculate adjusted (tilted) predictions for each zooplankton class^124,125^.

The model was checked for sensitivity to the RF input parameters. The number of trees in the ensemble was set to 500, by which the model error was found to stabilise for all zooplankton groups, indicating a sufficiently large forest. The number of variables to use at each tree node, *mtry*, was varied from 1 to *n*_p_, where *n*_p_ = 12 was the total number of variables; models were subsequently run using the value that minimised model error: 4, 7 and 8 for small copepods, *C. finmarchicus* and *C. helgolandicus*, respectively. Ten replicates of the RF models were run for each zooplankton class to check the sensitivity of the resulting variable importance ranking.

The models were tested for their ability to extrapolate in space and time. This was required for two reasons. First, because of the temporally and spatially patchy coverage of the CPR sampling. Second, because projecting a model trained on historical conditions is likely to involve predicting on novel variable ranges, combinations or relationships with the response^121^. Following best practice for spatiotemporal ecological modelling, we used a block cross-validation approach^82^. This splits the data into *k* spatial or temporal blocks, trains the RF models *k* times using the data from all but one block, and then assesses the ability of the model to predict on the left-out block. Strategic blocking is preferable to random subsetting because autocorrelation prevents such subsets from being statistically independent and can lead to an overestimation of extrapolation ability^81,82,121^. Here, we divided the domain into eight contiguous spatial blocks and three temporal blocks^126^ (see Supplementary Information). The predictive accuracy of the models on the withheld data was measured using root-mean-square error (RMSE) and the coefficient of determination (*R*^2^). Additionally, the variable importance ranking was compared between time periods to check the temporal stability of their influence on zooplankton energy.

We calculated which regions of the model domain would become “novel predictor space” under each climate projection and, therefore, require extrapolation by the machine learning models. This was calculated univariately and globally, i.e. by comparing the projections for each environmental variable at each grid-cell with the training data range for the whole model domain. These were combined over environmental variables to obtain a map showing where at least one variable would be outside the training range under each climate projection (Supplementary Information).

To assess the ecological relevance of the trained models, we compared the modelled historical spatial distributions and the relationships between each predictor and zooplankton class (partial distributions) with existing ecological knowledge.

### Past zooplankton variability

Models were developed for small copepods (*Centropages* spp., *Acartia* spp., *Oithona* spp., *Para-Pseudocalanus* spp., *Temora longicornis* and juvenile *Calanus* spp.) modelled in aggregate, and the dominant large calanoid copepod species *C. finmarchicus* and *C. helgolandicus*, modelled separately because of their distinct spatial and ecological niches.

Abundance of each species was derived from CPR sampling for 1998–2019 over the area 20°W to 12°E and 40°N to 65°N to match the temporal and spatial coverage of the ocean model data. The CPR survey is a large-scale, long-term marine sampling programme collecting plankton on reels of silk towed at approximately 10 m water depth by ships of opportunity^127^. Employing the method developed by Olin et al.^25^, sample counts were corrected for the CPR’s variability in sampling efficiency for different taxa^128,129^. The correction factors were calculated by comparing with independent plankton time series from Stonehaven on the Scottish east coast^130^ and the L4 station on the English south coast^131^. Diel vertical migration was accounted for by applying separate factors for samples collected during day and night. CPR counts were converted to energy density (kJ/m^3^) using taxon-specific wet weight and energy density values. No correction was attempted for variation in energy content with season or year. See Olin et al.^25^ for full details.

### Predictors of past variability in zooplankton

Predictors were derived from the Atlantic Margin Model 3D coupled physical–biogeochemical ocean model reanalysis (1993–2023) for the Northwest European Shelf (AMM7)^132^. AMM7 has ~7 km horizontal resolution. Each CPR sample was matched with predictors from the closest horizontal grid cell and time step.

Predictors included in the models were yearday, bathymetric depth (m), daily sea surface temperature (SST, °C), chlorophyll integrated over 0–100 m water depth (mg/m^3^), mixed layer depth (m) and sea surface salinity, monthly SST anomaly with respect to the 1993–2023 climatology (°C), monthly nitrate anomaly over 0–100 m depth with respect to the same period (mmol/m^3^) and annual mean winter nitrate concentration (January–March, i.e. prior to the phytoplankton spring bloom, mmol/m^3^).

Both SST and SST anomaly have been included in the model because they capture different ecological processes acting at different timescales. Daily SST represents absolute thermal conditions experienced by the zooplankton; the impact of daily SST will be a direct physiological response as well as a marker of habitat suitability (thermal preference) for different species. Monthly SST anomaly, on the other hand, represents changes in conditions relative to the climatology, e.g. heatwaves, cold spells or the climate warming signal. The two variables are not collinear (correlation coefficient of 0.16).

In addition, we included a proxy for the sub-polar gyre (SPG) strength: annual mean upper ocean (50–500 m) salinity anomaly at the model grid-cell having strongest historical correlation between upper ocean salinity and the SPG index time series^133^ (Pearson’s *R* = −0.7 at 12.1°W 54.1°N over (1993–2018), see Supplementary Information). The reason for including this proxy, rather than the SPG index itself, was to retain SPG strength when applying the models of zooplankton variability into the future, because although projections of the SPG strength do not exist, this same measure could be readily calculated in the climate projections. Including the SPG strength is important because it regulates the relative influences of subpolar and subtropical water masses and exerts bottom-up control on zooplankton assemblages in the Northeast Atlantic, e.g.^62^.

### Climate projections

Projections of future ocean conditions were taken from an ensemble of three coupled hydrodynamic-ecosystem simulations. Each simulation implemented NEMO-ERSEM on the AMM7 domain under the Intergovernmental Panel on Climate Change (IPCC) Representative Concentration Pathway “high emissions” scenario RCP8.5. The simulations differed in vertical resolution, NEMO version, ERSEM parameterisation, temporal resolution of output and which of the IPCC’s Coupled Model Intercomparison Project Phase 5 (CMIP5) global Earth System Model (ESM) was used for atmospheric and oceanic boundary forcing^134^ (Table 1).

The reanalysis and projections each used a different definition of mixed-layer depth. To ensure the predictors were equivalent in the training and projection models, mixed layer depth was therefore recalculated in the projections according to the definition used in the reanalysis: the depth at which the density increase compared to density at 3 m depth corresponds to a temperature decrease of 0.2 °C in surface conditions^132^.

Model output was at daily and monthly resolution. ROAM-HADGEM2 biogeochemical output was only available at a monthly resolution, preventing the calculation of phytoplankton bloom phenology for this model. Therefore, “reduced” RF models, excluding phytoplankton bloom start and duration, were used for zooplankton projections under this climate projection.

### Bias correction of climate projections

These long-term simulations project future trends in climate change; the timings of their internal variations are not expected to match observational records or future events^134^. In addition, all free-running climate projections exhibit bias, whereby they systematically deviate from mean historical observations due to imperfect representation of initial conditions, forcings and internal processes^135,136^. It is, therefore, standard practice to apply a bias correction before using the output^135,137,138^.

Here we used the “Delta change” method, which calculates the difference (Delta) in mean conditions between a reference historical and future period(s) in a projection and then applies Delta as a perturbation to a recent reference period in the reanalysis output, e.g.^136^. This treats the reanalysis as a proxy for observations, which is reasonable because the model has been validated against observations and assimilates satellite ocean colour and SST^139,140^. Deltas are calculated multiplicatively for predictors that can only take positive values (e.g. chlorophyll concentration) to avoid negative bias-corrected values and additively otherwise, e.g.^137^. Since the climate change signal will vary seasonally and regionally, e.g.^138^, we calculate the Delta change values by month (except for annual metrics such as bloom phenology), and by model grid-cell. The monthly Deltas are then interpolated to daily resolution and applied as a “climate change perturbation” to each year of the reanalysis reference period for the recent past (2000–2019). This obtains a bias-corrected future time series that preserves the climate change signal and realistic variability over a range of scales^141^. We apply this method for two future periods: 2040–2049 (near future) and 2090–2099 (far future). The RF models are then predicted on these time series to obtain zooplankton future projections for each zooplankton class and climate projection model.

## Supplementary information

Transparent Peer Review file

SUPPLEMENTARY INFORMATION

## Data Availability

The Continuous Plankton Recorder data are available from the Marine Biological Association at https://doi.org/10.17031/1673^142^ and https://doi.org/10.17031/64f6e4b80fb5c^143^. The regional ocean physical–biogeochemical model reanalysis data are available through Copernicus Marine Service (https://data.marine.copernicus.eu), products NWSHELF_MULTIYEAR_PHY_004_009 (https://doi.org/10.48670/moi-00059)^144^ and NWSHELF_MULTIYEAR_BGC_004_011 (https://doi.org/10.48670/moi-00058)^145^. The regional physical–biogeochemical model projection data are available through the British Oceanographic Data Centre at https://doi.org/10.5285/07877700-0e22-5fb5-e063-6c86abc03058^146^ and https://doi.org/10.5285/0786d770-ba54-0e57-e063-6c86abc09fdf^147^, and through Zendo at https://zenodo.org/records/3953801^148^.

## Code availability

The code developed for this analysis is available in PURE at https://doi.org/10.15129/6a0a0bf9-1407-438e-adcf-65edb92fc27a.
