# Supplementary material for: Projected declines in zooplankton energy supporting Northwest European Shelf ecosystems
Source: Commun Earth Environ. 2026 Jul 23;7(1):897. doi: 10.1038/s43247-026-03840-1 (PMC13759333; doi:10.1038/s43247-026-03840-1)
Supplement: Supplementary file 1 [file 43247_2026_3840_MOESM1_ESM.pdf]

nature portfolio

Peer Review File

Projected declines in zooplankton energy supporting Northwest European Shelf ecosystems

Corresponding Author: Dr Emma Tyldesley

This file contains all reviewer reports in order by version, followed by all author rebuttals in order by version.

Attachments originally included by the reviewers as part of their assessment can be found at the end of this file.

Version 0:

Reviewer comments:

Reviewer #1

(Remarks to the Author)
The manuscript by Tyldesley et al. presents future projections of key zooplankton taxa in the Northeast Atlantic under a high emissions climate scenario. The authors use a machine-learning approach based on regional downscaling of climate model output and continuous plankton recorder data. The models predict strong declines in these zooplankton taxa, up to 93 % for C. finmarchicus by the end of the century, primarily due to warming, freshening, and reduced nutrient concentrations. The declines are much stronger than what has been found in previous global studies. The use of regional downscaling and machine-learning implementing observational data is fairly novel, and the results are highly relevant, considering the importance of these zooplankton taxa as prey for higher trophic levels – as is well described in the Discussion. The paper is likely to be of interest for a larger audience, and to spark both debate and future investigations using alternative approaches or data.

The manuscript is clearly and concisely written, with proper reference to relevant literature. The figures are also clear and informative. The result section is written as a mix of results and a discussion, with comparison to other studies throughout. This is unusual (as there is a separate discussion section), but it makes the results easier to follow and reduces the need to repeat details from the results in the discussion section.

The statistical analyses are sound and fit for purpose, and a solid effort has been made to test the models' ability to extrapolate to new areas/time periods, thereby quantifying the uncertainty, and to bias-correct future projections.

Below are a few comments that the authors should address:
- The manuscript is framed as covering the Northeast Atlantic, but in essence it covers the Celtic Seas, Greater North Sea and Faroes (i.e. the distribution of CPR data and mainly shelf seas). Large parts of the main distribution area of C. finmarchicus in the NE Atlantic to the north and west of the study region are not included. This should be made clear, also in the abstract of the paper.
- On line 212, Figure 8, Figure 9 are referred to, but they are not included in the manuscript, as far as I can see. I suppose they have been moved to the supplementary material (4 and 5?). Also, Table 2 is referred to here, but does not contain information about projected changes.
- The random forest models explain ~42-46 % of the observed variability in abundance. This is further reduced when the data is split into blocks for cross validation (Table 1). Such (low) values are not unusual in SDMs of patchy zooplankton data, as discussed in the cited study by Brun et al. (2016, Global Change Biology). Although the general challenge of extrapolation beyond the range of the data is discussed (lines 344-346), a direct discussion and acknowledgement of the uncertainty in the results due to this low predictive power should be included. It is possible that more flexible techniques such as Boosted Regression Trees or ensemble models combining different techniques such as RF, BRTs and GAMs, could have improved the predictive power. Some arguments for why RF was preferred over these could have been included.
- It is not clearly explained why "yearday" was included as a variable in the statistical analysis, and how this was treated in the creation of predictions. Yearday was the most important predictor for small copepods and C. helgolandicus, and could capture some of the variation related to more ecologically meaningful variables (e.g. SST, chlorophyll, as mentioned on line 124), and will restrict the models' ability to predict future changes.
- On lines 171-175 the response to SST anomalies is described as hard to interpret, but that "the model predicts higher C.

finmarchicus and lower small copepod and C. helgolandicus abundance during unusually cold periods." But the plot also shows increased C. finmarchicus and C. helgolandicus during unusually warm periods, which is not commented on. Thus, although both species show a negative response to the highest SSTs (not anomaly), with a lower optimum for C. finmarchicus, which could be largely driven by the spatial distribution pattern of the species, they seem to respond positively to higher-than-usual temperatures (and lower than usual for C. finmarchicus) at any given site? What was the collinearity between SST and SST anomalies, is it justified to include both variables? Could you add some discussion on whether there is an ecologically plausible explanation for this bell-shaped response, or is it a model artefact? A few approaches that could help discern this would be to 1) test alternative models with only one of the variables included at the time and inspecting the response curves, 2) plotting the interaction between the two variables, 3) adding rugs to the x-axis of Fig. 4 to show the distribution of data (are there much data at the extremes of the anomalies?).
- The climate projections used in this study are based on CMIP5 forcing, instead of the more recent CMIP6 generation. Could you add a statement on why this was done (I assume because of availability of regional downscaling?) and comment on how the use of this older-generation model output may influence the results relative to using CMIP6-based projections?

Detailed comments:

• Figure 1 is referred to later than the other figures in the text.
• Consider using commas more often, many sentences are very long and should be split (for example, in front of "especially" on line 115, and "while" on line 217).
• L. 115: The seas should have "the" in front of the names.
• L. 132: "which may partly relate to SPG strength influence on water temperatures." – this is unclear, what do you mean? And what was the collinearity of these variables?
• L. 137-139: "We can also compare…": This sentence is hard to read and a bit unclear.
• L. 152: and vice versa what?
• L. 329: Grammatically incorrect sentence
• L. 396-397: This sentence is unclear, should it be taxa instead of classes? Or size classes? And add "different" in front of zooplankton.
• L. 443: "Variables were checked for pairwise collinearity prior to inclusion in model fitting." – which threshold did you use for excluding collinear variables?
• Some of the sentences in the methods section are very long, making this section a bit hard to read, and could be split for readability. For example lines 444-446.
• There are some instances of extra spacing around figure references in the text, please check this, e.g. (Figure 2) on line 111.

Reviewer #2

(Remarks to the Author)
Review of "Projected declines in zooplankton energy supporting Northeast Atlantic ecosystems" by Tyldseley et al

General Comments

The manuscript by Tyldesley and co-authors aims to model future distributions of key copepod species in the Northeast Atlantic under projected climatic and biogeochemical conditions. Specifically, the model focuses on localized changes within the North Sea and surrounding shelf habitats around Great Britain and Ireland, including the Armorican Shelf. The model applied a random forest approach that trained on 30 years to Continuous Plankton Recorder observations and predicted copepod abundances in the near and far future under RCP8.5 climatic scenarios. The authors report the main finding of large declines in two large calanoid copepods (Calanus finmarchicus and C. helgolandicus) and a suite of small copepods, with reductions of 58%–72% by 2050 and 84%–93% by 2100. The model attributes these declines to warming, freshening, and reduced nutrient availability.
The research question is timely and addresses a critically important aspect of predicting how future climate change may alter marine food webs. In particular, the study focuses on an ocean region of high relevance to international fisheries that has, to date, lacked specific downscaled modelling needed to support copepod-focused climate impact projections. Overall, I found the manuscript well written and easy to follow. However, parts of the Introduction and Discussion are quite terse and would benefit from editing to improve readability and flow. I am not an expert in the modelling approach used here, so my comments are primarily focused on interpretation and presentation of the results.

Major comments

My main concern relates to the decision to rely exclusively on climate projections under the RCP8.5 pathway. As the authors note, RCP8.5 "has been criticised as an overly pessimistic driving scenario for climate change impact assessment." I understand the rationale for using RCP8.5 as the strong climatic forcings produce clean and identifiable changes in ocean variables and therefore copepod responses. The authors note that this may help identify thresholds or potential tipping points which can be framed as a conservative worst-case-scenario of biological responses. Furthermore, many comparable studies cited in the discussion use the same worst-case-scenario climate projections. As written, the Abstract and Discussion emphasize substantial declines in copepod abundance with potentially far-reaching ecological consequences, but a section of the Discussion devotes several paragraphs to caveats that this scenario may be unlikely. This gives the impression that while the model may have performed well, the output is not very meaningful or informative. Furthermore, the

authors report that many other similar studies have worst-case-scenario pathways to model future copepods responses, and that the present study closely matches the directionality of the response. I think that this undermines the overall novelty of the study (i.e., what is ultimately different about this work vs. other regional modeling studies focused on calanoids that use RCP8.5).

I recommend that the authors address this in one of the following ways: (i) strengthen and clarify the justification for using only RCP8.5 given the stated aims (e.g., explicitly positioning the work as a worst-case-scenario), ensure the abstract reflects that framing that this is under RCP8.5, and the projected % declines are not necessarily predictive; and/or (ii) include at least one additional, more plausible pathway (i.e., RCP4.5) to quantify the magnitude of projected changes and reduce the risk of over-interpreting results tied to a single extreme scenario (see Burgess et al 2023; https://doi.org/10.1093/icesjms/fsad045). This could also be addressed through a targeted sensitivity analysis on a subset of the model experiments. I understand the later approach may necessitate a large amount of new work, so the authors should carefully consider how to best proceed to make their contribution most valuable.

Specific comments

1. Line 66-69: “However, global projections probably underestimate regional declines in zooplankton (Eddy et al., 2025; Grieve et al., 2017; Heneghan et al., 2023; Petrik et al., 2022; Reygondeau & Beaugrand, 2011) because of differences in how regions are responding to climate change and in how well they are represented by global ESMs.”

Just curious, do these models always underestimate declines?

2. Lines 246-248: “The machine learning models reproduced expected spatial distributions and relationships with predictor variables for each zooplankton group and explained approximately 45% of variability in historical abundance”

It would be helpful to include the relative performance. How does this compare to similar studies?

3. Lines 256-257: “Nutrient concentrations declined to values historically associated with low zooplankton density, presumably because these indicate a poor feeding environment such as low primary productivity or an unsuitable phytoplankton size or species composition.”

Could the authors provide more support or clarification for this inference? A reduction in primary productivity should broadly depress food availability for zooplankton taxa, whereas a shift in phytoplankton community structure could differentially affect groups and potentially favor different zooplankton species (including potential newcomers to the region).

4. Lines 293-296: “Theory and modelling suggest this is likely to continue with future climate change (Frederiksen et al., 2013; Kjesbu et al., 2022; Villarino et al., 2015) although there may be a limit to species’ ability to adapt (Edwards et al., 2021).”

This sentence is confusing. The first half indicates that copepod populations will continue to shift tracking their favored thermal conditions (i.e., acclimatization through range shifts). The second clause indicates that there is limit to adaptation (which should invoke genetic adaptation), but the citation discusses range shifts. Please clarify what is means by ‘limit to species’ ability to adapt’.

5. Lines 326-338. “326 The RCP8.5 represents a continued, unmitigated rise in emissions over the century and as such has been criticised as an overly pessimistic driving scenario for climate change impact assessment (e.g. Hausfather & Peters, 2020). RCP8.5 is used here because the strong forcing gives a clear physical and biological response which results in an interpretable signal of change in the zooplankton (Tinker et al., 2024); our study is intended to illustrate the trajectory rather than exact magnitude of change (Shepherd et al., 2018). Under more moderate emissions scenarios, the rate of decline may be slower than projected here; for example, under RCP4.5 the NF physical changes projected by RCP8.5 would be delayed until the end of century (IPCC, 2013). The use of RCP8.5 gives valuable information on a high impact-low likelihood ecological response that may yet be plausible under future climate policies and uncertain climate feedbacks or tipping points (Sarofim et al., 2024). Because other emissions scenarios project a similar direction of physical change, it is likely that the direction of change in copepod zooplankton abundance is robust even if the magnitude and speed are pessimistic.”

In this section, the authors attempt to justify their use of RCP8.5 to drive the models. They argue that RCP8.5 serves as a stress test that will most clearly show the direction of the biological response, and that the directionality of change in their model is consistent with other studies. If that is the primary aim, however, it is unclear what novel insight the present study adds beyond reinforcing an expected decline in copepod populations. The work would be more informative if it also explored a more realistic emissions pathway (or provided a sensitivity analysis) to better constrain the likely magnitude and timing of copepod declines in this region.

6. Lines 350-354: “Thus far, zooplankton populations appear to have responded to warming oceans by shifting their timing and distributions to remain with their environmental niches, rather than adapting their physiology to maintain their timing and distributions (Choquet et al., 2017; Chust et al., 2014; Hinder et al., 2014; Ji et al., 2010b; Reygondeau & Beaugrand, 2011).”

This statement seems too strong as written. There is evidence that copepod populations can undergo genetic adaptation to changing environmental conditions within the NF domain of this study (e.g., see the PNAS study:

https://www.pnas.org/doi/10.1073/pnas.2201521119). This could potentially buffer populations to persist locally for longer or slow the pace of range shifts. I recognize that modeling adaptive processes are difficult to represent and are usually not parameterized due to limited data. However, it would strengthen the discussion to acknowledge this explicitly and briefly outline how the absence of evolutionary adaptation (as well as other forms on multi-generational plasticity) in the model influence model certainty.

7. Lines 358-362: “However, model fit did decline with temporal cross-validation. By the NF, small areas of the domain (Bay of Biscay, Norwegian Trench and parts of the central and northern North Sea) shifted into novel environmental predictor space and by the FF increasing SST took the entire domain into novel predictor space (Supplementary Information). Therefore, the FF projections should be considered particularly uncertain.”

It is good that the authors acknowledge that the FF conditions under RCP8.5 put the domain into a novel predictor space, and this undermines the model. I think that this strengthens the case for including a more moderate forcing pathway, which may keep a larger fraction of the domain within (or closer to) the historical predictor space. This will provide projections with stronger support from the fitted relationships and a more informative framing of expected changes.

Reviewer #3

(Remarks to the Author)
The manuscript 'Projected declines in zooplankton energy supporting Northeast Atlantic ecosystems' by Tyldesley, Banas, Wakelin, Johns, Jardine, Searle, Daunt, Bull, Krivtsov, Heath and Speirs is investigating the future perspective of crustacean zooplankton in the North Atlantic under different future climate scenarios. Applying a newly developed 'high-resolution machine-learning model' for the key crustacean taxa Calanus finmarchicus, C. helgolandicus and small copepods, as well as investigating past and modelling future population developments by using Continuous Plankton Recorder observations as basis, the authors find remarkable changes in the future distribution pattern and abundances of the investigated groups. The author´s results indicate that crustacean zooplankton abundances will be dramatically impacted by anticipated future environmental conditions, with abundance declines in the Northeast Atlantic of of >60 to 90% for the near future (2050) and far future (2100), respectively. Not all investigated groups show the same magnitude of change. Irrespectively, those changes can be expected to have wide ranging consequences for food-web structure and functioning, as discussed in the manuscript.
I congratulate the authors to this excellent piece of work. I only have some minor comments that are outlined in the attached pdf. The only question and remark that needs special attention is the extraordinary high Calanus finmarchicus abundance in the last observation year, which is orders of magnitude higher than the previous observations (see Fig. 6 left panel) and come out in the analyses with an 800% higher off-shelf south density of this species (Fig. 7). As an ecologist, I feel that this might be an entry error in the underlying ecological dataset and should be double checked with the data provider to avoid that this observation point is pulling the analyses into a direction that might not be correct.
Once this is checked, substantiated and noted in the revised manuscript, I am looking forward seeing this manuscript being published with the highest priority as it is an important contribution to the literature which will be of value not only for the biological, ecological and modelling community, but also policy makers and stake-holders.

Version 1:

Reviewer comments:

Reviewer #1

(Remarks to the Author)
All my comments have been well addressed in the new version of the manuscript, and I congratulate the authors on this solid work and nicely written manuscript.

As noted by Reviewer 2 in the previous round, the manuscript would have benefited greatly from including a lower-emission scenario in addition to RCP8.5. And since the first review round in early 2026, the RCP8.5 scenarios has now been officially “retired”. Therefore, the relevance of the results from this study is questionable, a timing which is very unfortunate for the authors. As they note in the introduction of the manuscript, this was the only scenario for which regionally downscaled projections were available, which allowed them to conduct a more detailed and locally relevant analysis than what would have been possible using the latest global model outputs.
It is noted in the introduction that “This is a higher emission scenario than is currently considered likely given developments in global climate policy and industry (Burgess et al., 2023; Hausfather & Peters, 2020).” (This statement should be further emphasized with the recent development). The authors also clearly state in the introduction and the discussion that the results should be considered as qualitative/directional rather than quantitative predictions of zooplankton biomass for a particular time. In other words, the projected declines are likely to be realistic with continued climate change, but the magnitude cannot be directly trusted.

Considering that the authors clearly state these limitations, I think this manuscript is acceptable. However, there is the risk of the reported numbers of projected % decline in being directly used and re-cited without this context, and the study can easily by criticized for being outdated. The ideal solution would be to re-run the analyses with updated and more realistic regionally downscaled climate projections, but I understand that this might not be feasible at this time.

Reviewer #2

(Remarks to the Author)
Comments
I appreciate the authors' work in responding to my original comments, particularly the revisions made to clarify the rationale for using RCP8.5. It is helpful that the authors have now more explicitly framed the projections as a high-emissions scenario and have added language to the Abstract, Introduction, and Discussion to reduce the risk that the results are interpreted as precise forecasts. The explanation that comparable regionally downscaled projections were not available under a more moderate pathway is a reasonable practical constraint, and the authors have justified the value of using a strong-forcing scenario to identify the direction and spatial structure of potential biological responses.
My only further suggestion is that the authors amend the Conclusion section to include this important caveat, since this is the final framing of the study's implications that many readers will go to. For example, the sentence:
"This study shows the potential for declines in copepod zooplankton abundance on the Northwest European Shelf with future climate change that exceed those of global projections"
could be amended to:
"This study shows the potential for declines in copepod zooplankton abundance on the Northwest European Shelf with future climate change that exceed those of global projections under an extreme climate-forcing scenario."

Open Access This Peer Review File is licensed under a Creative Commons Attribution 4.0 International License, which permits use, sharing, adaptation, distribution and reproduction in any medium or format, as long as you give appropriate credit to the original author(s) and the source, provide a link to the Creative Commons license, and indicate if changes were made.
In cases where reviewers are anonymous, credit should be given to 'Anonymous Referee' and the source.
The images or other third party material in this Peer Review File are included in the article's Creative Commons license, unless indicated otherwise in a credit line to the material. If material is not included in the article's Creative Commons license and your intended use is not permitted by statutory regulation or exceeds the permitted use, you will need to obtain permission directly from the copyright holder.
To view a copy of this license, visit https://creativecommons.org/licenses/by/4.0/

Projected declines in zooplankton energy supporting Northwest European Shelf ecosystems

Tyldesley, Banas, Wakelin, Johns, Jardine, Searle, Daunt, Bull, Krivtsov, Heath & Speirs

Response to initial review

6 May 2026

We are grateful for these constructive and thorough reviews, which have allowed us to improve the content and clarity of the manuscript. We have addressed all the issues raised. Below, we outline our responses and the respective changes to the manuscript. The review comments are italicized, and our responses are in red.

Reviewer 1

Below are a few comments that the authors should address:
- The manuscript is framed as covering the Northeast Atlantic, but in essence it covers the Celtic Seas, Greater North Sea and Faroes (i.e. the distribution of CPR data and mainly shelf seas). Large parts of the main distribution area of C. finmarchicus in the NE Atlantic to the north and west of the study region are not included. This should be made clear, also in the abstract of the paper.

The author is correct. We have amended the manuscript throughout to refer to the study region more accurately as the “Northwest European shelf”. This is the standard name for this area (e.g. Tinker et al., 2024; Tonani & Ascione, 2021; Wakelin et al., 2020) and includes the North Sea, the Irish Sea, the English Channel and the immediately adjacent waters.

It is an important point that this region does not include the full main distribution of *C. finmarchicus* (Choquet et al., 2017). It is not possible to expand this study to include the more northerly distribution because the regional projections have a northern boundary of 64°N and CPR coverage in the Norwegian Sea is extremely limited spatially and temporally (see e.g. Tyldesley et al., 2024). This means that from our study we cannot infer the future *C. finmarchicus* distribution over the full Northeast Atlantic and whether, for example, the species will shift north into Arctic waters currently dominated by *C. glacialis* and *C. hyperboreus*. However, this does not affect the conclusions of the present study, which shows that even the most northerly regions of the Northwest European Shelf domain may become unsuitable for *C. finmarchicus* under future climate change. This has now been made clear in the manuscript (Discussion, Other caveats).

- On line 212, Figure 8, Figure 9 are referred to, but they are not included in the manuscript, as far as I can see. I suppose they have been moved to the supplementary material (4 and 5?). Also, Table 2 is referred to here, but does not contain information about projected changes.

Thank you for noticing this. This has been checked throughout, and all figure and table references should now be correct.

- The random forest models explain ~42-46 % of the observed variability in abundance. This is further reduced when the data is split into blocks for cross validation (Table 1). Such (low) values are not unusual in SDMs of patchy zooplankton data, as discussed in the cited study by Brun et al. (2016, Global Change Biology). Although the general challenge of extrapolation beyond the range of the data is discussed (lines 344-346), a direct discussion and acknowledgement of the uncertainty in the results due to this low predictive power should be included. It is possible that more flexible techniques such as Boosted Regression Trees or ensemble models combining different techniques such as RF, BRTs and GAMs, could have improved the predictive power. Some arguments for why RF was preferred over these could have been included.

We agree that, although the relevant analysis had been carried out, the uncertainty due to model skill had not been fully acknowledged in the paper. We have expanded the first paragraph of the Results as follows:

"The trained random forest models explained 43%, 46% and 42% of the observed variability in abundance for small copepods, *C. finmarchicus* and *C. helgolandicus*, respectively. The unexplained variance may include missing predictors and ecological processes, measurement error and non-stationary processes. This is a typical level of model skill for statistical plankton spatial distribution models (Brun et al., 2016). The explanatory power declined when testing the ability of the model to predict on areas of space or periods of time not included within the model training data using block cross-validation (Supplementary Information). Again, this is typical for statistical plankton models (Brun et al., 2016). The unexplained variance will weaken the model's ability to make precise forecasts; the zooplankton projections should instead be interpreted in terms of broad trends, differences due to the sensitivity of the climate models, species differences and relative spatial patterns."

RF was considered an appropriate statistical technique for this study, for reasons outlined in the manuscript: high predictive power, flexibility to data types, avoidance of over-fitting and insensitivity to outliers and noise. It outperformed other statistical techniques in a review of plankton SDMs (Brun et al. 2016). Since RF is non-parametric and data-driven, it naturally captures complex interactions between predictors without these needing to be known or specified *a priori*. This is an advantage over techniques such as GAMs.

We selected RF over a BRT or ensemble approach to achieve an appropriate balance of robustness and predictive power within the resource constraints of the project. RF is a relatively simple approach with minimal necessary parameterisation. This allowed us to conduct sensitivity analysis involving repeated training of the model. This was especially important given the other computationally expensive aspects of the project such as distilling the ocean model reanalysis and ensemble of climate projections (9TB of data each) into relevant predictor variables.

- It is not clearly explained why "yearday" was included as a variable in the statistical analysis,

and how this was treated in the creation of predictions. Yearday was the most important predictor for small copepods and C. helgolandicus, and could capture some of the variation related to more ecologically meaningful variables (e.g. SST, chlorophyll, as mentioned on line 124), and will restrict the models' ability to predict future changes.

This is a valid point, thank you. We agree that the use of yearday needed better explanation, given our focus on using ecologically meaningful variables. The reason for including yearday was to capture internally and externally driven copepod phenology not governed by the available seasonally varying predictors such as SST and chlorophyll, or better able to be represented by a mechanistic rather than statistical model. Such aspects will include:

- Lagged effects, e.g. dependence on a previous year's environmental conditions such as energy availability prior to diapause.
- Advection, e.g. dependence on conditions outside the model domain such as *C. finmarchicus* overwintering areas and the timing of arrival of *advected C. finmarchicus* into the North Sea.
- Integrated conditions, e.g. dependence on chlorophyll accumulated over a certain time period rather than daily standing value.
- Internally programmed phenology, e.g. partial dependence of breeding timing on day length.

In our model, therefore, yearday is intended to allow the RF model to capture some of the complex seasonality exhibited by copepods that is not governed directly by the available environmental variables. Yearday also acts as a proxy for day length, which is invariant under climate change. It was considered useful to retain yearday in the model to capture the response of zooplankton to both changing seasonality (e.g. warmer springs) and fixed seasonality (e.g. light availability). We acknowledge that inclusion of yearday may cause the model to underestimate the extent of phenological shift under climate change. Although yearday emerges as a strong predictor for small copepods and *C. helgolandicus* abundance, however, it is not completely dominant, and other variables also have a strong influence. Therefore, the RF model can (and does – see paragraph 4 of the section "Zooplankton climate projections") respond to those variables such as SST that vary with climate change.

The following statement has been added to Model building section: "Although a proxy variable, yearday was included in the model to represent internally and externally driven copepod phenology not governed by the available seasonally varying predictors, such as the partial dependence of breeding timing on day length and the timing of arrival of *C. finmarchicus* advected into the study region. Including yearday allows the model to capture the response of zooplankton to both changing seasonality (e.g. warmer springs) and fixed seasonality (e.g. light availability) under future climate change."

- On lines 171-175 the response to SST anomalies is described as hard to interpret, but that "the model predicts higher C. finmarchicus and lower small copepod and C. helgolandicus abundance during unusually cold periods." But the plot also shows increased C. finmarchicus and C. helgolandicus during unusually warm periods, which is not commented on. Thus, although both species show a negative response to the highest SSTs (not anomaly), with a lower

optimum for C. finmarchicus, which could be largely driven by the spatial distribution pattern of the species, they seem to respond positively to higher-than-usual temperatures (and lower than usual for C. finmarchicus) at any given site? What was the collinearity between SST and SST anomalies, is it justified to include both variables? Could you add some discussion on whether there is an ecologically plausible explanation for this bell-shaped response, or is it a model artefact? A few approaches that could help discern this would be to 1) test alternative models with only one of the variables included at the time and inspecting the response curves, 2) plotting the interaction between the two variables, 3) adding rugs to the x-axis of Fig. 4 to show the distribution of data (are there much data at the extremes of the anomalies?).

This is an important point that we hadn't adequately dealt with in the paper.

SST and monthly SST anomaly have low collinearity: the correlation coefficient is 0.16. Both also have low collinearity with the SPG proxy, the other variable likely to encode temperature signals (see response to L 132 comment below). The monthly SST anomaly is normally distributed. The reason for including both variables is that they capture different ecological processes acting at different timescales. Daily SST represents absolute thermal conditions experienced by the zooplankton. The impact of daily SST will be direct physiological response as well as a marker of habitat suitability (thermal preference) for different species. Monthly SST anomaly, on the other hand, represents changes in conditions relative to the climatology, e.g. heatwaves, cold spells or the climate warming signal.

We have added this justification to the Methods section "Predictors of past variability in zooplankton" paragraph 3.

The SST anomaly response curve is difficult to interpret for several reasons. The curve integrates the zooplankton response over season (month), space, and – in the case of the small copepods – also species. It is not straightforward to disaggregate these signals. Zooplankton abundance will respond differently to the same SST anomaly depending on the region and time of year. For example, a positive SST anomaly could result in *Calanus finmarchicus* abundance increasing in northern regions and decreasing in the North Sea. This would explain why the SST anomaly curve is bell shaped for both Calanus species. Warmer than normal spring months might lead to a longer growing season and higher zooplankton abundance, but warmer than normal water masses might be associated with lower productivity. In addition, the global warming signal of increasingly warm months might be associated with declining abundance.

We have amended lines 171-175 in line with the discussion above.

- The climate projections used in this study are based on CMIP5 forcing, instead of the more recent CMIP6 generation. Could you add a statement on why this was done (I assume because of availability of regional downscaling?) and comment on how the use of this older-generation model output may influence the results relative to using CMIP6-based projections?

This is an important point and the reviewer is correct – no regionally downscaled projections for CMIP6 forcing were available for this project. This means that our zooplankton projections are based on CMIP5 RCP8.5 rather than CMIP6 SSP5-8.5. The RCP8.5 and SSP5-8.5 scenarios have

similar levels of total radiative forcing, but CMIP6 models have higher climate sensitivity and project stronger warming that CMIP5 models (Tebaldi et al., 2021; Zelinka et al., 2020). Therefore, the projected zooplankton changes under RCP8.5 are likely to be a lower bound on SSP5-8.5.

This explanation has been added to the discussion section.

Detailed comments:

• Figure 1 is referred to later than the other figures in the text.

This has been checked and Figure 1 is the first figure referred to. Figures and tables in the Supplementary Information have been renamed with an S suffix to avoid confusion.

• Consider using commas more often, many sentences are very long and should be split (for example, in front of "especially" on line 115, and "while" on line 217).

We agree. We have amended the document throughout to use more commas and split sentences to improve readability.

• L. 115: The seas should have "the" in front of the names.

Amended here and throughout the manuscript.

• L. 132: "which may partly relate to SPG strength influence on water temperatures." – this is unclear, what do you mean? And what was the collinearity of these variables?

This statement was misleading and has been removed – the SPG strength proxy is not collinear with SST (correlation coefficient R=0.065) or monthly SST anomaly (R=0.0015), although the SPG does influence temperatures in some parts of the model domain (mainly to the north and west).

• L. 137-139: "We can also compare…": This sentence is hard to read and a bit unclear.

This sentence was unclear and has been revised: "We can also compare the predicted relationships with existing ecological knowledge. These can sometimes be difficult to interpret if a variable has multiple, or spatially and temporally varying, impacts on zooplankton abundance."

• L. 152: and vice versa what?

This was unclear. Amended to read: "As expected, a strong sub-polar gyre was associated with increased *C. finmarchicus* and reduced *C. helgolandicus*, and vice versa"

• L. 329: Grammatically incorrect sentence

We have broken this sentence into two parts and added commas to improve readability.

• *L. 396-397: This sentence is unclear, should it be taxa instead of classes? Or size classes? And add "different" in front of zooplankton.*

Yes, this was unclear. We have rewritten: "Climate change is expected to have differential impacts on different zooplankton taxa"

• *L. 443: "Variables were checked for pairwise collinearity prior to inclusion in model fitting." – which threshold did you use for excluding collinear variables?*

We used a threshold of Pearson's correlation = 0.7. This has now been added to the manuscript.

In checking this value, we also noticed an inaccuracy where we state that "A complimentary index of relative Arctic water influences in the Norwegian Sea (Skagseth et al., 2022) was initially included in the model but subsequently removed due to high collinearity with the SPG proxy." In fact, this index was omitted not because of collinearity, but because it is not available into the future and so could not be used in the final models. This sentence has now been removed.

• *Some of the sentences in the methods section are very long, making this section a bit hard to read, and could be split for readability. For example lines 444-446.*

We have revised the whole manuscript (including the lines 444-446 mentioned above) to improve readability by adding commas and splitting sentences. We hope there is now a good balance of brevity and clarity.

• *There are some instances of extra spacing around figure references in the text, please check this, e.g. (Figure 2) on line 111.*

This was a formatting error and has been corrected throughout.

Reviewer 2

However, parts of the Introduction and Discussion are quite terse and would benefit from editing to improve readability and flow.

This was also noted by Reviewer 1, and we have now edited the whole manuscript to add commas, simplify and break up over-long sentences. We hope this has improved the readability and flow.

Major comments

My main concern relates to the decision to rely exclusively on climate projections under the

RCP8.5 pathway. As the authors note, RCP8.5 "has been criticised as an overly pessimistic driving scenario for climate change impact assessment." I understand the rationale for using RCP8.5 as the strong climatic forcings produce clean and identifiable changes in ocean variables and therefore copepod responses. The authors note that this may help identify thresholds or potential tipping points which can be framed as a conservative worst-case-scenario of biological responses. Furthermore, many comparable studies cited in the discussion use the same worst-case-scenario climate projections. As written, the Abstract and Discussion emphasize substantial declines in copepod abundance with potentially far-reaching ecological consequences, but a section of the Discussion devotes several paragraphs to caveats that this scenario may be unlikely. This gives the impression that while the model may have performed well, the output is not very meaningful or informative. Furthermore, the authors report that many other similar studies have worst-case-scenario pathways to model future copepods responses, and that the present study closely matches the directionality of the response. I think that this undermines the overall novelty of the study (i.e., what is ultimately different about this work vs. other regional modeling studies focused on calanoids that use RCP8.5).

I recommend that the authors address this in one of the following ways: (i) strengthen and clarify the justification for using only RCP8.5 given the stated aims (e.g., explicitly positioning the work as a worst-case-scenario), ensure the abstract reflects that framing that this is under RCP8.5, and the projected % declines are not necessarily predictive; and/or (ii) include at least one additional, more plausible pathway (i.e., RCP4.5) to quantify the magnitude of projected changes and reduce the risk of over-interpreting results tied to a single extreme scenario (see Burgess et al 2023; https://doi.org/10.1093/icesjms/fsad045). This could also be addressed through a targeted sensitivity analysis on a subset of the model experiments. I understand the later approach may necessitate a large amount of new work, so the authors should carefully consider how to best proceed to make their contribution most valuable.

Thank you to the reviewer for expanding our view of this debate around use of RCP8.5 and the consequences for communication of these results to the oceanographic community and beyond. We agree that the justification and consequences of using a high-emissions scenario needed to be strengthened to avoid misinterpretation of the results.

We have used RCP8.5 for several reasons.

First, availability: there were no comparable, regionally downscaled projections available under a more moderate pathway.

Second, the use of a single emissions scenario has allowed us to use a projection ensemble rather than single model, which allows clear illustration of the impact of model climate sensitivity on the regional zooplankton projections. We have demonstrated a clear link between parent (global forcing) model climate sensitivity and copepod response. The models we use span almost the full range of CMIP5 climate sensitivities: they have an Equivalent Climate Sensitivity, defined as the degrees of warming resulting from an instantaneous doubling of CO_2,

of 2.39-4.59 K (2.08-4.67 for the full CMIP5 ensemble) (Andrews et al., 2012). The zooplankton projections, therefore, will also span the range of uncertainty for climate projections.

Third, there is much uncertainty inherent in climate projections. The use of RCP8.5 gives valuable information on a "high impact-low likelihood" ecological response that may yet be plausible under future climate policies and uncertain climate feedbacks or tipping points.

Fourth, we consider the use of RCP8.5 to be appropriate because the primary aim of the study was to generate regional projections where currently only global projections exist, and because the strong forcing gives a clear response which results in an interpretable signal of change in the zooplankton. Given the smooth response of the zooplankton abundance to the climate models, i.e. with no tipping points or abrupt extinctions, we expect that the results under a more plausible pathway such as RCP4.5 would differ by degree and timing rather than sign.

Much of the intensive work of this study has been in the development of the novel method (conversion of CPR data, separation of crustacean zooplankton into separately modelled groups, development of sub-polar gyre proxy, derivation of predictors, etc). When downscaled projections become available for more moderate RCPs or SSPs, this new framework could be applied to a range of emissions pathways and/or ensembles of models to provide more quantitative projections.

However, we agree with the reviewer that this was not appropriately communicated in the manuscript, leaving the possibility of misinterpretation by researchers and potentially the media. We have, therefore, revised the text to clearly state the rationale for using a high-emissions scenario within the aims of the project and clarify that the projections indicate qualitative change rather than absolute predictions.

Specific changes to the text are as follows:

- The last paragraph of the introduction has been expanded to "The RCP8.5 represents a continued, unmitigated rise in emissions over the century and, as such, has been criticised as an overly pessimistic driving scenario for climate change impact assessment (Burgess et al., 2023; e.g. Hausfather & Peters, 2020). It is, therefore, important that the projected declines in zooplankton be interpreted qualitatively, and in comparison with global declines, rather than as quantitative values for specific time periods. RCP8.5 is used here because the strong forcing gives a clear physical and biological response, which results in an interpretable signal of change in the zooplankton (Tinker et al., 2024). Our study is intended to illustrate the trajectory, rather than exact magnitude, of change (Shepherd et al., 2018). No regionally downscaled projections were available for a more moderate emissions pathway. Under more moderate emissions scenarios, the rate of decline may be slower than projected here; for example, under RCP4.5, the near future physical changes projected by RCP8.5 would be delayed until the end of the century (IPCC, 2013). The use of RCP8.5 gives valuable information on a high impact, low likelihood ecological response that may yet be plausible under future climate policies and uncertain climate feedbacks or tipping points (Sarofim et al., 2024). Because other emissions scenarios project a similar

direction of physical change, it is likely that the direction of change in copepod zooplankton abundance is robust even if the magnitude and speed are pessimistic.

- Second paragraph of discussion expanded to: "Application of the models to projected future environmental conditions under a high-emissions scenario resulted in large declines in abundance for all copepod zooplankton groups and all climate models."
- Addition to section How likely is this future? Paragraph 3 "No regionally downscaled projections were available for a more moderate emissions pathway."
- The Abstract has been amended to include "We applied it to an ensemble of regional climate projections for the Northwest European Shelf under the high emissions scenario RCP8.5 to enable a clear signal of climate-driven change in zooplankton abundance."

Regarding the reviewer's comment that "this undermines the overall novelty of the study (i.e., what is ultimately different about this work vs. other regional modeling studies focused on calanoids that use RCP8.5).", we emphasize that this is the first zooplankton projection for this area that uses regionally-downscaled climate models.

Specific comments

1. Line 66-69: "However, global projections probably underestimate regional declines in zooplankton (Eddy et al., 2025; Grieve et al., 2017; Heneghan et al., 2023; Petrik et al., 2022; Reygondeau & Beaugrand, 2011) because of differences in how regions are responding to climate change and in how well they are represented by global ESMs."

Just curious, do these models always underestimate declines?

This is a good point – global models must overestimate change for some regions. These lines have now been edited to say "...global projections probably underestimate some regional declines in zooplankton...".

2. Lines 246-248: "The machine learning models reproduced expected spatial distributions and relationships with predictor variables for each zooplankton group and explained approximately 45% of variability in historical abundance"

It would be helpful to include the relative performance. How does this compare to similar studies?

Although there are few studies with which to directly compare the performance of our model, Brun et al (2016) show that this is a typical model skill for statistical plankton distribution models.

3. Lines 256-257: "Nutrient concentrations declined to values historically associated with low zooplankton density, presumably because these indicate a poor feeding environment such as

low primary productivity or an unsuitable phytoplankton size or species composition."

Could the authors provide more support or clarification for this inference? A reduction in primary productivity should broadly depress food availability for zooplankton taxa, whereas a shift in phytoplankton community structure could differentially affect groups and potentially favor different zooplankton species (including potential newcomers to the region).

The reviewer is correct. This paragraph was poorly worded and conflates several mechanisms – nutrient concentrations, phytoplankton species composition and plankton size spectrum. While all of these are likely to influence zooplankton abundance, only nutrient concentration is included in the model. An earlier incarnation of the model included phytoplankton size fractions, inspired by studies suggesting that zooplankton declines are due to a shift to smaller phytoplankton dominance under low nutrient conditions (Schmidt et al., 2020). The phytoplankton size fractions consistently ranked of lowest importance and so were excluded from further modelling.

Lines 256-257 have been reworded accordingly:

"Nutrient concentrations declined to values historically associated with low zooplankton density, presumably because these broadly indicate low primary productivity and depressed food availability for zooplankton, i.e. bottom-up control."

4. Lines 293-296: "Theory and modelling suggest this is likely to continue with future climate change (Frederiksen et al., 2013; Kjesbu et al., 2022; Villarino et al., 2015) although there may be a limit to species' ability to adapt (Edwards et al., 2021)."

This sentence is confusing. The first half indicates that copepod populations will continue to shift tracking their favored thermal conditions (i.e., acclimatization through range shifts). The second clause indicates that there is limit to adaptation (which should invoke genetic adaptation), but the citation discusses range shifts. Please clarify what is means by 'limit to species' ability to adapt'.

We agree that this was confusing, and in danger of conflating range shifts with other types of adaptation. We have rewritten this paragraph as follows:

"In general, North Atlantic copepod zooplankton distributions have shifted north with warming seas as species track their preferred temperature ranges (e.g. Beaugrand et al., 2009; Ratnarajah et al., 2023). Theory and modelling suggest zooplankton range shifts are likely to continue with climate change (Frederiksen et al., 2013; Kjesbu et al., 2022; Villarino et al., 2015). However, habitat availability and physical barriers such as currents may limit a species' ability to adjust its distribution, leading to eventual range squeeze and population decline, e.g. as observed for krill in the North Atlantic (Edwards et al., 2021)."

In addition, we have added a discussion on phenological plasticity and genetic adaptation by copepod zooplankton – see response to Comment 6 below.

5. Lines 326-338. "326 The RCP8.5 represents a continued, unmitigated rise in emissions over the century and as such has been criticised as an overly pessimistic driving scenario for climate change impact assessment (e.g. Hausfather & Peters, 2020). RCP8.5 is used here because the strong forcing gives a clear physical and biological response which results in an interpretable signal of change in the zooplankton (Tinker et al., 2024); our study is intended to illustrate the trajectory rather than exact magnitude of change (Shepherd et al., 2018). Under more moderate emissions scenarios, the rate of decline may be slower than projected here; for example, under RCP4.5 the NF physical changes projected by RCP8.5 would be delayed until the end of century (IPCC, 2013). The use of RCP8.5 gives valuable information on a high impact-low likelihood ecological response that may yet be plausible under future climate policies and uncertain climate feedbacks or tipping points (Sarofim et al., 2024). Because other emissions scenarios project a similar direction of physical change, it is likely that the direction of change in copepod zooplankton abundance is robust even if the magnitude and speed are pessimistic."

In this section, the authors attempt to justify their use of RCP8.5 to drive the models. They argue that RCP8.5 serves as a stress test that will most clearly show the direction of the biological response, and that the directionality of change in their model is consistent with other studies. If that is the primary aim, however, it is unclear what novel insight the present study adds beyond reinforcing an expected decline in copepod populations. The work would be more informative if it also explored a more realistic emissions pathway (or provided a sensitivity analysis) to better constrain the likely magnitude and timing of copepod declines in this region.

Please see response above to the reviewer's main concern. We would further emphasise that the novelty of this study lies in the generation of regional projections where only global projections currently exist, in the development of the novel modelling framework and the disaggregation of copepods into key species.

6. Lines 350-354: "Thus far, zooplankton populations appear to have responded to warming oceans by shifting their timing and distributions to remain with their environmental niches, rather than adapting their physiology to maintain their timing and distributions (Choquet et al., 2017; Chust et al., 2014; Hinder et al., 2014; Ji et al., 2010b; Reygondeau & Beaugrand, 2011)."

This statement seems too strong as written. There is evidence that copepod populations can undergo genetic adaptation to changing environmental conditions within the NF domain of this study (e.g., see the PNAS study: https://www.pnas.org/doi/10.1073/pnas.2201521119). This could potentially buffer populations to persist locally for longer or slow the pace of range shifts. I recognize that modeling adaptive processes are difficult to represent and are usually not parameterized due to limited data. However, it would strengthen the discussion to acknowledge this explicitly and briefly outline how the absence of evolutionary adaptation (as well as other forms on multi-generational plasticity) in the model influence model certainty.

We agree with the reviewer and have expanded the paragraph:

“First, the changing environment may force zooplankton adaptation and new biotic interactions. Thus far, zooplankton populations appear to have responded to warming oceans by shifting their timing and distributions to remain with their environmental niches (Choquet et al., 2017; Chust et al., 2014; Hinder et al., 2014; Ji et al., 2010b; Reygondeau & Beaugrand, 2011). However, organisms can also cope with rapid environmental change through phenotypic plasticity (acclimation through shifts in body physiology or morphology within the lifetime of the organism) or evolution (adaptation through genetic selection of traits over several generations). This can buffer populations from declines under environmental stress. Both plasticity (e.g. Hahn & Brennan, 2024) and adaption (e.g. Brennan et al., 2022; Dam et al., 2021; Kelly et al., 2017) have been demonstrated in copepod zooplankton, leading to rapid but incomplete recovery of population fitness under environmental stress. The extent of recovery can be limited by fitness trade-offs, especially under the action of multiple environmental stressors such as is the case with climate change (Dam et al., 2021; deMayo et al., 2025). By not explicitly representing adaptive mechanisms, which would be computationally expensive and difficult to parameterise due to lack of data, the model may be overestimating the copepod population declines to some extent.”

7. Lines 358-362: “However, model fit did decline with temporal cross-validation. By the NF, small areas of the domain (Bay of Biscay, Norwegian Trench and parts of the central and northern North Sea) shifted into novel environmental predictor space and by the FF increasing SST took the entire domain into novel predictor space (Supplementary Information). Therefore, the FF projections should be considered particularly uncertain.”

It is good that the authors acknowledge that the FF conditions under RCP8.5 put the domain into a novel predictor space, and this undermines the model. I think that this strengthens the case for including a more moderate forcing pathway, which may keep a larger fraction of the domain within (or closer to) the historical predictor space. This will provide projections with stronger support from the fitted relationships and a more informative framing of expected changes.

Please see response above to the reviewer’s main concern and to Reviewer 1’s third comment regarding uncertainty in the future projections.

Reviewer 3

I congratulate the authors to this excellent piece of work. I only have some minor comments that are outlined in the attached pdf. The only question and remark that needs special attention is the extraordinary high Calanus finmarchicus abundance in the last observation year, which is orders of magnitude higher than the previous observations (see Fig. 6 left panel) and come out in the analyses with an 800% higher off-shelf south density of this species (Fig. 7). As an ecologist, I feel that this might be an entry error in the underlying ecological dataset and should be double checked with the data provider to avoid that this observation point is pulling the

analyses into a direction that might not be correct.
Once this is checked, substantiated and noted in the revised manuscript, I am looking forward seeing this manuscript being published with the highest priority as it is an important contribution to the literature which will be of value not only for the biological, ecological and modelling community, but also policy makers and stake-holders.

We are grateful for these encouraging remarks.

Line 29: wide ranging

Amended as suggested.

Line 49: Crustacean zooplankton, especially copepods

Amended as suggested.

Line 52: already and reference that is 13 years old does not fit, either change references to include newer ones of remove already

We agree that the use of this word was jarring and have amended as suggested.

Line 90: can be misunderstood as invasive species. It has always been part of the North Sea ecosystem, but they now contribute more to mesozooplankton biomass across larger areas of the North Sea, see Bonnett et al. 2005 Progress in Oceanography volume 65, issue 1, April 2005. Pages 1-53, that might be worth citing as well

We agree that this was misleading and that Bonnet et al. 2005 is the appropriate reference. We have rewritten lines 90-94 as follows:

"*C. helgolandicus* is a temperate species occurring mainly on the continental shelf from the Mediterranean Sea to the North Sea (Bonnet et al., 2005). Since the 1960s, the core range of *C. finmarchicus* has shifted north with warming oceans and the core range of *C. helgolandicus* has expanded in all directions (Chust et al., 2014). In the North Sea, *C. finmarchicus* abundance has declined and *C. helgolandicus* has increased, so that it is now dominant where the two species overlap (Chust et al., 2014; R. Wilson et al., 2016)."

Line 191: I would write NF = near future out, it is not much longer and avoids confusion. Especially as you have a very large number of abbreviations that are used throughout the manuscript. Abbreviations demand a high level of alertness from the reader. So please re-consider abbreviations here and throughout the manuscript. You will provide a service to the reader and ensure that this manuscript is read across a large readership.

We agree that this would help readability and have expanded the acronyms NF and FF throughout the text and figures.

Line 192: Far future (provide your time horizon for this as well)

Amended as suggested.

Lines 206-210: spell out far future and provide a time horizon what this means in this study.

Amended as suggested and time horizons provided in lines 192-196.

Line 270: exactly, so the strongest perturbations and deviations from current conditions is also expected and predicted here to lead to the most dramatic change. I would ass as expected. Please keep in mind that is all given the current environmental envelopes of species. This can change as adaption and evolution is known to act on contemporary time scales and can rescue populations – even though it might not rescue in response to multiple pressures to undisturbed conditions. You might want to read and include into the discussion this recently published review on this issue: Recognizing adaptation costs in the Anthropocene (2025) J. A. deMayo, R. S. Brennan., M. Pespeni, et al. Trends in Ecology and Evolution DOI: https://doi.org/10.1016/j.tree.2025.09.020.

We agree and have amended Line 270 to include "as would be expected". Reviewer 2 also suggested including a discussion around plasticity and adaptation by copepod zooplankton under environmental stress. We have added this to the Discussion, including reference to deMayo et al 2025. Please see response to Reviewer 2 comment 6 above.

Line 287: is this a proper citation? Can the data be added in the supplement? Please check and modify accordingly.

We agree that this is a useful and proper addition, and have now included the details of extracting the Petrik et al. 2022 global projections for the domain under consideration in this study (Northwest European Shelf). The method was to calculate the area-weighted mean of grid-cells of the global models that fell within our model domain. The results are now shown here as colour plots and a summary table. Please see new section "Supplementary Information: Petrik et al. (2022) global zooplankton climate projections in the Northwest European Shelf domain".

Line 289: super important point that might be highlighted up front in the discussion section?!

We agree that the differential impact of warming and AMOC decline on the northeast Atlantic is a crucial point that we had not stressed sufficiently in the manuscript. We have strengthened Line 289 to include the most relevant references: "...due to temperatures rising faster than the

global mean (von Schuckmann et al., 2024) and the particular sensitivity of this region to a reduced AMOC (Jackson et al., 2015; Liu et al., 2020; Rahmstorf, 2024)." We have also highlighted this upfront in paragraph 4 of the introduction.

Line 295: Add TREE review as outlined above deMayo et al. 2025

Thank you for this very useful and thought-provoking reference. A discussion on adaptation, including reference to deMayo et al. 2025, is now included in the Discussion in the section "How likely is this future?" Please see also the response to Reviewer 2 comment 6.

Line 297: explain what you mean by this term instead of citing it here without context. I do not know what exactly you mean but this. So be precise and do not leave it to the reader to infer what you mean.

We agree this was unclear and have added the following definition: "the population is so depleted that it no longer plays a significant role in the ecosystem, even if individuals still exist".

Line 387: references are in bold, but should not be highlighted

We are unable to see any bold or highlighted references so am unsure what this comment refers to. Hopefully it was a formatting error that is now resolved, but please let us know if this is still a problem.

Line 393: please add after appendicularians "(larvaceans)" and include the reference Jaspers et al 2023 that you cite later on

Amended as suggested.

Line 401: I would also add Jaspers et al 2023 in here, as the issue of being favoured by climate change has extensively been reviewed in the paper

Reference added as suggested.

Line 497: please add reference here. I assume all goes back to Olin et al. 2022? Anyhow, good to be explicit here! Thanks!!

Reference added as suggested.

Line 988: ??

Typing error amended.

Line 1010: it would be useful to have this figure in terms of abundance in the supporting material

Since this plot already shows spatial abundance, we interpret this comment to mean that the reviewer would like to see the *future* spatial abundance in the Supplementary Material. We agree that this is a useful addition and have added those plots – see Figure S6.

Line 1014: same comment as below, species names should be provided in italics, please change here and throughout the manuscript.

The whole manuscript has now been checked and amended where necessary. Species names are now Italicized in Figures 2, 3, 4, S2 and S5.

Line 1019: plot with continental shelf break is missing a box around, as done in all other figures

Thank you for noticing this. Now amended to include missing box.

Line 1021: Very interesting Fig. Is this new information that has been compiled for this study or been downloaded from other sources. Would be good to indicate in the Fig. legend. Also recent past need a time horizon and the abbreviations should be explained shortly in the Fig. legend.

These are the downscaled projections obtained from the British Oceanographic Data Centre and the National Oceanography Centre (see Table 1) and bias corrected as part of this study (see Methods section "Climate projections".

We have amended this figure to add recent past horizon period, expand acronyms, tidy up by moving colour bars to the end of each row, add units, and provide more explanation in the legend: "Bias corrected far future (2090-2100) mean decadal sea surface temperature, salinity and winter nitrate concentration for each climate projection model. The past ocean conditions are taken from the Northwest European Shelf model reanalysis provided by the Copernicus Marine Service. ROAM-HAGEM2, RECICLE-IPSL and RECICLE-GFDL are the regionally downscaled climate projection models that have been biased corrected for this study. See Table 1."

Line 1028: But this is driven by one extreme observation (annual mean) in the recent past. As an ecologist, I would say that this is an outlier that needs confirmation. Can you trace if this is a database error and check the underlying data if it makes sense?! This can drive your analysis even though the certainty of being a wrong observation is quite high. So I would call for double checking and ground truthing of the data for that year.

If we understand correctly, the reviewer is relating the large increase in *C. finmarchicus* in the "offshelf, south" region seen in Figure 7(a) to the recent high *C. finmarchicus* abundance seen the middle-left panel of Figure 6. We are glad that this has been raised because it is a point of

potential confusion that we have tried to address in the Figure caption but clearly not adequately.

The recent high off-shelf *C. finmarchicus* abundance seen in Figure 6 extends over several years (approximately 2013-2019), not just one outlier year, and is therefore likely to be real and not a data error. We could suggest that it is related to the strong sub-polar gyre over those years (see Figure S1). Recent relatively high *C. finmarchicus* abundance can also be seen for the shelf region (Figure 6 middle-right panel).

The apparently large percentage increase in *C. finmarchicus* in Figure 7 is for the southern offshelf region. Historically, this region has almost zero abundance of *C. finmarchicus*, a species associated with low-salinity subarctic water masses further north. The climate projections give reduced future salinity in the southern offshelf region. This causes the RF model to project a small absolute future increase in *C. finmarchicus* in the southern offshelf region. Because percentage change is calculated as the change in abundance divided by the past abundance, and because the past abundance is near zero, this results in a very large *percentage change* for *C. finmarchicus* in that region, even though the resulting *absolute abundance* is still small compared with the core areas for *C. finmarchicus*. This can be seen in Figure S4, which is why we have taken care in that figure to plot all regions on the same y-axis scale. This is also why in Figure 7 we have plotted both percentage change (panel a) and absolute abundance (panel b).

We included an explanation of this in the caption of Figure 7. We have strengthened the text as follows:

"Note that the *C. finmarchicus* increase in the offshelf south region (800%) extends beyond the upper limit of the percentage change axis, which is curtailed to allow interpretation for the other regions. The reason for this apparently large relative change is that historical *C. finmarchicus* abundance in this region is negligible, since the species is associated with subarctic water masses further north (see Figure S4). Declining future salinity in that region causes the model to projection a small absolute increase in *C. finmarchicus* abundance, which results numerically in a large percentage change even though the resulting projected densities are still low in comparison with historical densities in the core regions for this species (see panel b and Figure S4)."

Line 1077: Species names should be provided in italic, please change

Amended as requested.

Line 1080: I would add a cross link to your table 1 in the main manuscript, where you explain the different models and name that these are different projections or similar

We agree this will be helpful to the reader and have added a link to Table 1 to the figure caption.

Line 1085: Y-axis units are missing. Please add here and throughout the manuscript. One might also exchange long text on the left-hand side with images from the main text Fig. and relate to the images in the Fig. legend

Figure amended to include y-axis units and legend expanded to give more detail. Since this figure is in the supplement, we have decided to keep the long text on the left-hand side rather than replace with images so that this figure is self-contained. We have also amended Figure 6 to include the zooplankton units.

Line 1100: I would add a sentence on the consequences of this analysis to provide perspective for the non-expert reader. Also it might be good to modify some of the predictors to more standardised units, e.g. what is the meaning of mo. Chl should be Chl a ug L-1? This can be incorporated into the legend.

We agree, and the issue of the consequences of this analysis was also raised by Reviewer 1. We have expanded the first paragraph of the Results section to make this explicit.

Figure S6 and its legend have been amended to expand the acronyms. This figure does not require units because the colours show regions where each variable is outside the range of the training data, rather than showing the values of the variables. We have checked all other figures to make sure units are included and acronyms are avoided to keep the figures self-contained. The units of Chl a in the model are mg/m^3.

Projected declines in zooplankton energy supporting Northwest European Shelf ecosystems

Tyldesley, Banas, Wakelin, Johns, Jardine, Searle, Daunt, Bull, Krivtsov, Heath & Speirs

Response to final review

29 June 2026

We are grateful to the two reviewers for taking the time to re-review our manuscript. Their thoughtful comments have been extremely useful.

Reviewers' comments:
Reviewer #1 (Remarks to the Author):

All my comments have been well addressed in the new version of the manuscript, and I congratulate the authors on this solid work and nicely written manuscript.

As noted by Reviewer 2 in the previous round, the manuscript would have benefited greatly from including a lower-emission scenario in addition to RCP8.5. And since the first review round in early 2026, the RCP8.5 scenarios has now been officially "retired". Therefore, the relevance of the results from this study is questionable, a timing which is very unfortunate for the authors. As they note in the introduction of the manuscript, this was the only scenario for which regionally downscaled projections were available, which allowed them to conduct a more detailed and locally relevant analysis than what would have been possible using the latest global model outputs.
It is noted in the introduction that "This is a higher emission scenario than is currently considered likely given developments in global climate policy and industry (Burgess et al., 2023; Hausfather & Peters, 2020)." (This statement should be further emphasized with the recent development). The authors also clearly state in the introduction and the discussion that the results should be considered as qualitative/directional rather than quantitative predictions of zooplankton biomass for a particular time. In other words, the projected declines are likely to be realistic with continued climate change, but the magnitude cannot be directly trusted.

Considering that the authors clearly state these limitations, I think this manuscript is acceptable. However, there is the risk of the reported numbers of projected % decline in being directly used and re-cited without this context, and the study can easily by criticized for being outdated. The ideal solution would be to re-run the analyses with updated and more realistic regionally downscaled climate projections, but I understand that this might not be feasible at this time.

Thank you for these helpful comments. We agree and have rephrased the sentence in the introduction to "This is a higher emission scenario than is currently considered plausible given developments in global climate policy and industry" and cited Van Vuuren, D. P., O'Neill, B. C., Tebaldi, C., Sanderson, B. M., Chini, L. P., Friedlingstein, P., Hasegawa, T., Riahi, K.,

Govindasamy, B., Bauer, N., Eyring, V., Fall, C. M. N., Frieler, K., Gidden, M. J., Gohar, L. K., Högner, A., Jones, A. D., Kikstra, J., King, A., ... Ziehn, T. (2026). The Scenario Model Intercomparison Project for CMIP7 (ScenarioMIP-CMIP7). Geoscientific Model Development, 19(7), 2627–2656. https://doi.org/10.5194/gmd-19-2627-2026.

We have reiterated this in the Discussion: "The RCP8.5 represents a continued, unmitigated rise in emissions over the century and, as such, is now considered an overly pessimistic driving scenario for climate change impact assessment, with higher emissions than the "high end" scenario in CMIP7".

Reviewer #2 (Remarks to the Author):

Comments

I appreciate the authors' work in responding to my original comments, particularly the revisions made to clarify the rationale for using RCP8.5. It is helpful that the authors have now more explicitly framed the projections as a high-emissions scenario and have added language to the Abstract, Introduction, and Discussion to reduce the risk that the results are interpreted as precise forecasts. The explanation that comparable regionally downscaled projections were not available under a more moderate pathway is a reasonable practical constraint, and the authors have justified the value of using a strong-forcing scenario to identify the direction and spatial structure of potential biological responses.

My only further suggestion is that the authors amend the Conclusion section to include this important caveat, since this is the final framing of the study's implications that many readers will go to. For example, the sentence:

"This study shows the potential for declines in copepod zooplankton abundance on the Northwest European Shelf with future climate change that exceed those of global projections" could be amended to:

"This study shows the potential for declines in copepod zooplankton abundance on the Northwest European Shelf with future climate change that exceed those of global projections under an extreme climate-forcing scenario."

We agree and have reworded the first sentence of the conclusions as suggested.

Projected declines in zooplankton energy supporting Northeast Atlantic ecosystems

Tyldesley, EJ[1,*], Banas, NS[1,2], Wakelin, S[3], Johns, DG[4], Jardine, JE[3], Searle, K[5], Daunt, F[5], Bull, C[2,6], Krivtsov, V[1], Heath, MR[1], Speirs, DC[1]

[1] Department of Mathematics and Statistics, University of Strathclyde, 26 Richmond Street, Glasgow, G1 1XH, United Kingdom

[2] Atlantic Salmon Trust, Orchard House, Kilgraston Walled Garden, Bridge of Earn, PH2 9HN, United Kingdom

[3] National Oceanography Centre Liverpool, Joseph Proudman Building, 6 Brownlow Street, Liverpool, L3 5DA, United Kingdom

[4] The Marine Biological Association, The Laboratory, Citadel Hill, Plymouth, PL1 2PB

[5] UK Centre for Ecology & Hydrology, Bush Estate, Penicuik, Midlothian, EH26 0QB, United Kingdom

[6] Biological and Environmental Sciences, University of Stirling, Stirling, United Kingdom

* Corresponding author. E-mail: emma.tyldesley@strath.ac.uk

ABSTRACT

The abundances of zooplankton species supporting Northeast Atlantic food webs have declined over the past 60 years, and their future is uncertain given continuing climate change. We developed a new high-resolution machine-learning model of the key taxa, driven by past ocean conditions and trained on Continuous Plankton Recorder observations. We applied it to an ensemble of regional climate projections under a high emissions scenario. The results projected large declines in Northeast Atlantic abundance: ensemble mean of 58%-72% by 2050 and 84%-93% by 2100 for small copepods and large *Calanus*. However, the projections were not geographically uniform, with evidence of regional refugia. The future declines were primarily driven by increasing temperature and decreasing salinity and nutrient concentrations, which shifted conditions away from the species' environmental niches and water mass associations. Impacts on food webs could be severe unless alternative zooplankton assemblages emerge that can support existing planktivorous fish and the predators that depend on them.

MAIN

Climate change is driving huge restructuring of global zooplankton assemblages (Heneghan et al., 2023; Ratnarajah et al., 2023). General responses of zooplankton to warming oceans are range shifts, reduced body size and altered phenology (Ratnarajah et al., 2023). Zooplankton species are moving polewards to maintain their temperate niches (e.g. Beaugrand et al., 2009; Edwards et al., 2021; Hinder et al., 2014). Warming can reduce zooplankton community mean body size, either directly through altered metabolic rates or indirectly through an environment that favours smaller species (Daufresne et al., 2009; Schmidt et al., 2020). Warming of surface ocean layers increases water column stability, reducing nutrient supply and primary productivity. Altered seasonality of temperature and phytoplankton dynamics in turn affect zooplankton phenology and productivity (Ji et al., 2010a; Richardson, 2008). In addition, climate change is altering ocean circulation, currents, winds and river run-off, all of which affect environmental conditions and dispersal patterns for zooplankton (Edwards et al., 2013). In combination, these pressures are resulting in altered zooplankton community compositions, size distributions,

seasonality and abundance (e.g. Beaugrand et al., 2009; Bedford et al., 2020; Edwards et al., 2021; Heneghan et al., 2020; Richardson, 2008; Schmidt et al., 2020). These changes are highly regional (Edwards et al., 2013).

The aim of this study was to model future changes in the key zooplankton supporting Northeast Atlantic food webs. The Northeast Atlantic is a productive, biodiverse and economically important region that is subject to multiple and expanding anthropogenic stressors (BEIS, 2022; OSPAR, 2023b). In the Northeast Atlantic, the dominant zooplankton group, copepods, is in a long term decline which is expected to continue (Holland et al., 2023; OSPAR, 2023a). Copepod zooplankton are crucial in the efficient transfer of energy from phytoplankton to higher trophic levels such as fish, seabirds and mammals (Fauchald et al., 2011; Frederiksen et al., 2006; Lynam et al., 2017). Evidence suggests that climate-driven changes in zooplankton are already affecting higher predators (Beaugrand & Reid, 2013; Dalpadado et al., 2024; MacDonald et al., 2015, 2019; Olin et al., 2020, 2022; Tyldesley et al., 2024).

How ongoing climate change will affect zooplankton is highly uncertain. The current generation of global Coupled Model Intercomparison Project (CMIP) Earth System Models (ESMs) project declining zooplankton abundance (Petrik et al., 2022). However, accurate representation of zooplankton is not a focus of these models; the zooplankton terms are a large source of uncertainty that may be causing biases in climate projections (Rohr et al., 2023). An alternative approach to projecting zooplankton under climate change is to couple the physical and lower trophic level output of global climate models with zooplankton-focused statistical and mechanistic models (Banas, Møller, et al., 2016; Banas, Wilson, et al., 2016; Ji et al., 2010b; Ratnarajah et al., 2023; Richardson, 2008).

A global trait-based zooplankton model driven by an ensemble of CMIP6 climate projections showed 7%-16% declines in global mean zooplankton by the end of the century under a range of emissions scenarios (Heneghan et al., 2023). However, global projections probably underestimate regional declines in zooplankton (Eddy et al., 2025; Grieve et al., 2017; Heneghan et al., 2023; Petrik et al., 2022; Reygondeau & Beaugrand, 2011) because of differences in how regions are responding to climate change and in how well they are represented by global ESMs. Coastal and shelf seas, such as those of the Northeast Atlantic, are poorly represented by global models because of coarse spatial resolution and a lack of coastal ocean processes (Holt et al., 2017). Climate change impact studies for this area therefore require a regional downscaling approach (Drenkard et al., 2021; Holt et al., 2018; Tinker et al., 2015, 2016, 2024; Wakelin et al., 2020a). In the Northeast Atlantic, climate change is expected to result in warming, freshening, reduced circulation, reduced nutrients and increased water column stratification, but there is much uncertainty around the degree and trajectory of change (Tinker et al., 2016, 2024).

In this study, we generated high resolution climate projections for the Northeast Atlantic (Figure 1) for the key copepod zooplankton supporting planktivorous fishes and their predators. Models were developed for small copepod species modelled in aggregate, and the dominant large calanoid copepod species *Calanus finmarchicus* and *Calanus helgolandicus,* which were modelled separately because of their distinct spatial and ecological niches. Calanus zooplankton are lipid-rich, abundant zooplankton providing the key link between primary production and higher predators in the Northeast Atlantic (Melle et al., 2014; Record et al., 2018). This is the main prey for many planktivorous fish such as sandeel, sprat, herring and mackerel and the larvae of piscivorous species such as salmon and blue whiting. *C. finmarchicus* is a boreal species dominating mesozooplankton biomass across the North Atlantic. In Arctic seas, it is replaced by the larger, polar congener species *C. glacialis* and *C. hyperboreus* (Choquet et al., 2017). *C. helgolandicus* is a temperate species occurring mainly on the continental shelf from

the Mediterranean Sea to the North Sea. Since the 1960s, the core range of *C. finmarchicus* has shifted north with warming oceans and *C. helgolandicus* has expanded into the North Sea, where it is now dominant where the two species overlap (Chust et al., 2014).

Random Forest machine learning algorithms were used to model historical variability in copepod zooplankton abundance, derived from Continuous Plankton Recorder sampling, as a function of physical and biogeochemical predictor variables from a regional ocean model reanalysis for the period 1998 to 2023. Predictors included in the models were yearday, bathymetric depth, daily sea surface temperature (SST), chlorophyll, mixed layer depth and sea surface salinity, monthly SST anomaly and upper ocean nitrate anomaly, annual winter nitrate concentration and an annual salinity-based proxy for the sub-polar gyre (SPG) strength. We applied the zooplankton models to the same set of predictors from an ensemble of regionally downscaled bias-corrected ocean model CMIP5 climate projections to 2100 under the Representative Concentration Pathways RCP8.5 emissions scenario (Table 1). Zooplankton projections were extracted for the near future (NF, 2040s; +2.0°C global surface temperature anomaly compared with pre-industrial conditions) and far future (FF, 2090s; +3.7°C) (IPCC, 2013)

RESULTS

Model structure and comparison with ecological knowledge

The trained random forest models explained 43%, 46% and 42% of the observed variability in abundance for small copepod, *C. finmarchicus* and *C. helgolandicus* respectively. The model predictions for present day (mean over 2010-2020) spatial distributions of each zooplankton class matched existing knowledge, with the warm temperate species *C. helgolandicus* confined primarily to the shelf and shelf-edge, the sub-polar species *C. finmarchicus* occupying deeper colder waters, and some overlap in the northern North Sea (Figure 2) (Choquet et al., 2017; Continuous Plankton Recorder Survey Team, 2004; Helaouët & Beaugrand, 2007; Wilson et al., 2015). It is harder to make a direct comparison for our small copepod class, but published distributions for each individual species indicate that in aggregate the abundance should be as predicted by our model: concentrated on the shelf especially North Sea and Celtic Sea and extending off-shelf especially to the northwest (Continuous Plankton Recorder Survey Team, 2004).

Random Forest models calculate a measure of “variable importance”, i.e. how strongly each explanatory variable influences the model predictions. The importance ranking for each zooplankton group was stable, with only minor reordering of similarly ranked variables with each repetition of model training (Figure 3). The strongest influence on the abundance of small copepods and the temperate shelf species *C. helgolandicus* was yearday, reflecting strong seasonality – zooplankton abundance in high latitude shelf seas is highly seasonal due to the strong variation in light, temperature, water column stratification and nutrient availability, which drives seasonal variation in phytoplankton as well as directly affecting zooplankton metabolism (Simpson & Sharples, 2012). For small copepods, the next strongest influences were salinity, temperature, sub-polar gyre strength, mixed layer depth, SST anomaly and nitrate anomaly, each having similar ranking. For *C. helgolandicus*, the SPG strength, SST, salinity and depth formed the next most influential group of predictors, reflecting the importance of water mass variability, seasonality and shelf habitat for this species.

The most important predictor for *C. finmarchicus* abundance was the SPG strength, followed by SST anomaly, which may partly relate to SPG strength influence on water temperatures. The next group of most influential predictors were nitrate anomaly, SST and winter nitrate.

Figure 4 shows the marginal effect (partial dependency) of each variable on the predicted zooplankton abundance for each species. This shows how the variables have affected zooplankton abundance historically and, in combination with future projections of each variable, how zooplankton abundance may vary into the future. We can also compare the predicted relationships with existing ecological knowledge, although they are not always easy to interpret if there are multiple or spatially and temporally varying processes. The partial dependencies indicate that the model successfully captures expected seasonal cycles, bathymetric, temperature and salinity niches, and water mass associations of the different zooplankton classes. The model correctly predicts a steep rise in zooplankton abundance from April/May and gradual decline over September-November (Fanjul et al., 2018). *C. helgolandicus* peaks in both spring and autumn in the Northeast Atlantic while *C. finmarchicus* and small copepods have one peak the timing of which varies regionally (Planque & Fromentin, 1996; Wilson, 2015).

The model captures the transition from small copepod and *C. helgolandicus* dominance on the continental shelf (< 200 m water depth) to *C. finmarchicus* dominance in deeper water (Helaouët & Beaugrand, 2007). The model predicts peak *C. finmarchicus* abundance at 0-9°C SST and below 35 salinity, and peak *C. helgolandicus* abundance at 13-17°C and above 35 salinity (Bonnet et al., 2005; Helaouët & Beaugrand, 2007; Melle et al., 2014; Reygondeau & Beaugrand, 2011; Wilson, 2015). As expected, a strong sub-polar gyre was associated with increased *C. finmarchicus* and vice versa (Hátún et al., 2009, 2016). The model also predicted a switch from small copepods and *C. helgolandicus* to *C. finmarchicus* dominance with increasing winter nitrate levels as found previously (Helaouët & Beaugrand, 2007).

The model shows a saturation numerical (population) response with chlorophyll concentration, which is a proxy for the concentration of zooplankton's phytoplankton prey. This threshold response is likely to result from the interplay of the zooplankton ingestion functional response, losses from metabolic costs and predation, and varying suitability of the phytoplankton community composition (Daewel et al., 2014; Gentleman et al., 2003; Mitra et al., 2014). The increase in zooplankton abundance at mixed layer depth less than 100 m is in line with the increase in phytoplankton productivity that results from seasonal stratification (Simpson & Sharples, 2012); the relationship with *C. finmarchicus* abundance appears to be more complicated and possibly reflects bathymetric influences or a mixed layer definition that is less appropriate off-shelf (Kara et al., 2000).

The model results show that small copepod and *C. helgolandicus* abundance peaked when phytoplankton blooms started April-May, whereas *C. finmarchicus* was more abundant for May-June blooms and decreased for longer bloom duration. This is probably related to the tendency for a later, shorter productive season with increasing latitude (Friedland et al., 2016), favouring taxa like *C. finmarchicus* that specialise in high energy storage and winter dormancy (Banas, Møller, et al., 2016; Melle et al., 2014; Wilson et al., 2016).

The partial dependencies for winter nitrate and monthly anomaly in SST and nitrate are harder to interpret and probably combine latitudinal, water mass, seasonal and climate change signals. In general, the model predicts higher *C. finmarchicus* and lower small copepod and *C. helgolandicus* abundance during unusually cold periods and a decline in small copepod abundance with warming.

Physical-biogeochemical climate projections

The direction, magnitude and regionality of change of each of the predictor variables differs between the regional climate projections, despite all of them incorporating the same carbon emissions pathway (FF bias corrected SST, salinity and nutrients illustrated in Figure 5; NF and

FF change in all variables given in SI Figure 3). This is as expected from the range of physical projections made by their global forcing models (IPCC, 2013). A key difference among the global projections is the degree to which climate forcing is projected to weaken the Atlantic Meridional Overturning Circulation (AMOC), which affects water mass transport and circulation patterns on the Northwest European shelf (IPCC, 2013). All three regional simulations used in this study project reduced Atlantic water salinity and circulation, seen as water mass freshening in all regions, but the change is minor for GFDL, dramatic but slow for HADGEM, and dramatic and rapid for IPSL (SI Figure 3). The shutdown of exchange between the Atlantic and North Sea and reduced circulation, salinity and nutrient concentrations in the North Sea has been described as a "novel potential future state for the North Sea" (Holt et al., 2018, using the HADGEM simulation).

As a result of the rapid weakening of circulation, IPSL projects cooling into the NF (by up to 0.4°C) while the other models project warming. All simulations project significant warming by the FF. All simulations project an increase in stratification (reduction in mean annual mixed layer depth) in all regions, as expected (Tinker et al., 2024).

The variation in physical projections affects the biogeochemical projections. Chlorophyll is projected to increase in the NF in the north off-shelf and shelf regions, particularly for IPSL, and decrease in the south off-shelf region. By the FF, it increases for all simulations in all regions except in the southern off-shelf region for IPSL. Both nitrate measures decrease into the NF and FF, except for HADGEM which shows an FF increase for the shelf and south off-shelf regions. All simulations project a shift in phytoplankton bloom phenology: earlier blooms in most regions, longer blooms in the south off-shelf and shelf regions, and shorter blooms for the north off-shelf region.

Zooplankton climate projections

The varying physical-biogeochemical climate projections combined with the differential influence of each environmental predictor on zooplankton density translated into a range of zooplankton climate projections (Figure 6, Figure 7 and Table 2). However, zooplankton density was projected to decline for all zooplankton classes over most of their current range. The ensemble mean decline averaged over the Northeast Atlantic was 58% for small copepods, 72% for *C. finmarchicus* and 64% for *C. helgolandicus* by the NF and 84% for small copepods, 93% for *C. finmarchicus* and 84% for *C. helgolandicus* by the FF.

Into the NF, the rate and magnitude of decline varied by physical-biogeochemical climate projection, zooplankton class and region (Figure 8, Figure 9 and Table 2). The GFDL projection gave the weakest declines in zooplankton density; HADGEM and especially IPSL resulted in stronger declines. For example, GFDL projected a decline in small copepod density of 24% on-shelf and 49% off-shelf by the NF, while HADGEM (59% on-shelf and 84% off-shelf) and IPSL (71% on-shelf and 87% off-shelf) projections were in closer agreement. In contrast, IPSL projected a 90% decline in NF off-shelf *C. finmarchicus* abundance while GFDL and HADGEM gave similar declines of 65% and 63% respectively. Although seemingly stark, comparison with CPR observations suggests these projected declines are comparable with recent trends for small copepods and *C. finmarchicus* (Figure 6).

By the FF there were major declines in zooplankton density for all models for most areas. The projections were similar between models for small copepod (declines of 88% to 91% off-shelf and 76% to 88% on-shelf) and *C. finmarchicus* (declines of 92% to 95% off-shelf and 83% to 96% on-shelf) density but more divergent for *C. helgolandicus* (declines of 53% to 69% off-shelf and

80% to 92% on-shelf). Overall, the HADGEM projection gave the most pessimistic results by the FF.

There was no consistent pattern in whether declines were highest on or off-shelf. Overall, *C. finmarchicus* had the largest projected declines out of the modelled zooplankton groups into the NF and FF. The HADGEM and IPSL models indicated a timing shift in peak small copepod and *C. helgolandicus*, but not *C. finmarchicus*, abundance from spring to autumn; otherwise, there were no obvious changes in the zooplankton seasonality at the monthly scale analysed here (SI Figure 5).

At regional scale (Figure 1), there were projections that diverged from the shelf/off-shelf scale trends (Figure 7). There were regions such as the English Channel, southern North Sea, Celtic Sea, Irish Sea and Norwegian Trench where declines in zooplankton were less severe for some or all modelled species. In addition, all models projected increases in *C. finmarchicus* density in the southern half of the off-shelf region. However, the resulting densities were still low in comparison with historical densities within the core range for this species. Species distribution modelling typically has lower skill in areas outside the main distribution of a species where prevalence is low and historical observations are highly variable (Brun et al., 2016). This divergence of trends suggests the potential for regional refugia where zooplankton density may be sufficiently maintained to support existing food chains but does not change the overall projection of large declines in copepod abundance across most of the Northeast Atlantic.

DISCUSSION

Projected zooplankton declines

The machine learning models reproduced expected spatial distributions and relationships with predictor variables for each zooplankton group and explained approximately 45% of variability in historical abundance. The strongest predictors were yearday, surface salinity and temperature, nitrate concentration and prevailing water mass. In this study, the declines in copepod zooplankton were driven by the projected changes in temperature, salinity and nutrient concentrations (Figure 5). Northeast Atlantic temperatures increased above the preferred range for each zooplankton group, leading to lower projected densities in previous core areas. Salinity was projected to decline to levels below those of the Atlantic and mixed Atlantic-shelf waters historically associated with high densities of *C. helgolandicus* and small copepods, whilst favouring *C. finmarchicus*. Nutrient concentrations declined to values historically associated with low zooplankton density, presumably because these indicate a poor feeding environment such as low primary productivity or an unsuitable phytoplankton size or species composition. For *C. finmarchicus*, the primary driver was temperature: although projected salinities were within the preferred range, future warming shrunk the temperature contours north until by the FF the area within its optimal range (<9 °C) had shrunk to a small region north of the Faroe Islands, effectively excluding the species from the model domain. For *C. helgolandicus* and small copepods, the primary driver was freshening of water masses and reduced circulation: the salinity contours shift south and out of the North Sea so that only the southern-most part of the domain is within the preferred salinity range (<35).

Application of the models to projected future environmental conditions resulted in large declines in abundance for all copepod zooplankton groups and all climate models. *C. finmarchicus* was the worst affected taxa. HADGEM gave the largest declines. This was the model with the largest future reduction in Atlantic circulation, resulting in a North Sea with reduced oceanic input, slowed circulation and increasingly estuarine conditions, as seen in several regional climate

projections (Holt et al., 2018; Tinker et al., 2016). The model with the least change in circulation and sea surface salinity, GFDL, resulted in the smallest zooplankton declines.

There was regional variation in projected zooplankton abundance, with the smallest declines in the English Channel, southern North Sea, Celtic Sea, Irish Sea and Norwegian Trench. These are the areas with lowest connection to the Atlantic ocean inflows to the North Sea where environmental conditions are least affected by projected changes in ocean water mass properties and circulation (Tinker et al., 2024). They also coincide with the areas that have not had recent declines in copepod zooplankton (OSPAR, 2023a).

Comparison with other projections

The zooplankton declines in this study are large in comparison with global projections. Heneghan (2023) reported 7% to 16% decline in total zooplankton under a range of emissions scenarios and models. For their zooplankton group and emissions scenario closest to those used in this study (omnivorous zooplankton under SSP5-8.5), global declines were around 14% to 22% and the mapped results indicate that declines in the Northeast Atlantic were larger than elsewhere. CMIP6 ESMS project global change in (predominantly copepod) zooplankton of approximately +5 to -18% by 2051-2100 under RCP8.5 (Petrik et al., 2022). However, these projections show large regional variation (Petrik et al. 2022 Supplementary Information) and the mean change for the Northeast Atlantic varies from -7% to -64% by 2051-2100 (data supplied by Petrik). Clearly there is the possibility for changes in zooplankton biomass to be much larger in the Northeast Atlantic than at global scale due to temperatures rising faster than the global mean and the particular sensitivity of this region to a reduced AMOC.

There are limited regional studies with which to compare our results. In general, North Atlantic copepod zooplankton distributions have shifted north with warming seas as species track their preferred temperature ranges (e.g. Beaugrand et al., 2009; Ratnarajah et al., 2023). Theory and modelling suggest this is likely to continue with future climate change (Frederiksen et al., 2013; Kjesbu et al., 2022; Villarino et al., 2015) although there may be a limit to species' ability to adapt (Edwards et al., 2021). Environmental niche modelling shows *C. finmarchicus* occurring with such low probability as to be "functionally extirpated" in northwest European shelf seas by the end of the century as a result of changes in SST alone (Reygondeau & Beaugrand, 2011). In the Northeast Atlantic, *C. finmarchicus* is expected to expand north and west into Nordic Seas and decline in the North Sea, southern Norwegian Sea and southern regions (Kjesbu et al., 2022). On the other side of the Atlantic, northeast United States continental shelf *C. finmarchicus* abundance has been projected to decline by 50% by 2100 under RCP8.5 (Grieve et al., 2017). Since the 1960s, warming seas have caused *C. helgolandicus* to expand north and replace *C. finmarchicus* in the North Sea (Chust et al., 2014; Tyldesley et al., 2024). Future expansion depends on whether *C. helgolandicus* is limited to the continental shelf (Wilson, 2015). This species typically overwinters at depth in the relatively shallow shelf seas, which remain favourable for survival throughout the year, rather than entering diapause (dormancy) as required by oceanic species. Nutrient-poor open ocean environments lack this overwintering habitat. Further, reduced circulation between the North Sea and Atlantic Ocean may restrict dispersal of *C. helgolandicus* into potential new ocean habitat even if surface water temperatures are within the preferred range. If *C. helgolandicus* is restricted to shelf seas, then ongoing climate change would eventually lead to range contraction and abundance decline.

How likely is this future?

Assessing the plausibility of these projected declines in copepod zooplankton can be divided into several questions: how likely is the projected physical-biogeochemical future, how likely is the

projected zooplankton response to this future environment, and how much uncertainty is inherent in the machine learning modelling approach?

The overall physical projections for the Northeast Atlantic of warming, freshening, reduced circulation and increased water column stratification are in line with global and regional projections (IPCC, 2013, 2021; Tinker et al., 2016, 2024). The ensemble spread is within the range of other projections for this region but both IPSL and HADGEM have circulation and salinity changes towards the high end of projected response; the projected zooplankton declines are also therefore expected to be at the high end of what would be seen for a broader ensemble of climate projections. The biogeochemical projections are consistent with this physical response and observations of recent trends (Holt et al., 2018; OSPAR, 2023b; Wakelin et al., 2020a).

The RCP8.5 represents a continued, unmitigated rise in emissions over the century and as such has been criticised as an overly pessimistic driving scenario for climate change impact assessment (e.g. Hausfather & Peters, 2020). RCP8.5 is used here because the strong forcing gives a clear physical and biological response which results in an interpretable signal of change in the zooplankton (Tinker et al., 2024); our study is intended to illustrate the trajectory rather than exact magnitude of change (Shepherd et al., 2018). Under more moderate emissions scenarios, the rate of decline may be slower than projected here; for example, under RCP4.5 the NF physical changes projected by RCP8.5 would be delayed until the end of century (IPCC, 2013). The use of RCP8.5 gives valuable information on a high impact-low likelihood ecological response that may yet be plausible under future climate policies and uncertain climate feedbacks or tipping points (Sarofim et al., 2024). Because other emissions scenarios project a similar direction of physical change, it is likely that the direction of change in copepod zooplankton abundance is robust even if the magnitude and speed are pessimistic.

Given this physical-biogeochemical future, the modelled zooplankton projections make ecological sense if the species continue to track their historical environmental niches. The projected declines are in line with long-term and recent trends in observed copepod zooplankton abundance and range shifts (Beaugrand et al., 2009; Beaugrand & Reid, 2013; Corona et al., 2024; Olin et al., 2022; OSPAR, 2023a; Tyldesley et al., 2024) and modelling studies (see above). Extrapolation of statistical spatiotemporal distribution models in space, time and environmental predictor space, as necessitated by climate change impact studies, is challenging (Brun et al., 2016; Elith & Leathwick, 2009; Meyer et al., 2019; Roberts et al., 2017). Projections may be less certain if the relationships between zooplankton abundance and environmental predictors fail to persist – either because the changing environment forces zooplankton adaptation and new biotic interactions or because the relationships captured by the model are associative rather than functional (Elith & Leathwick, 2009). Thus far, zooplankton populations appear to have responded to warming oceans by shifting their timing and distributions to remain with their environmental niches, rather than adapting their physiology to maintain their timing and distributions (Choquet et al., 2017; Chust et al., 2014; Hinder et al., 2014; Ji et al., 2010b; Reygondeau & Beaugrand, 2011). To address the second issue, we selected environmental variables likely to have a functional relationship with zooplankton abundance, avoided proxy variables such as latitude and longitude, confronted the model with existing ecological knowledge and checked for stability of the model structure across the time span of the data. However, model fit did decline with temporal cross-validation. By the NF, small areas of the domain (Bay of Biscay, Norwegian Trench and parts of the central and northern North Sea) shifted into novel environmental predictor space and by the FF increasing SST took the entire domain into novel predictor space (Supplementary Information). Therefore, the FF projections should be considered particularly uncertain.

Other caveats

In this study we make the assumption of predominantly bottom-up control of copepod zooplankton, justified given that zooplankton track environmental variation especially over long time scales (e.g. Hátún et al., 2016; Richardson, 2008). This will not always be the case – for example, larval fish exert significant predation pressure on zooplankton (Fauchald et al., 2011; Heath, 2007), but observations of larval fish abundance are not available at sufficient resolution to include within our study framework. We do not explicitly represent any species interactions such as competition or predation by other copepod zooplankton. We neglect any species-specific responses to climate change within the aggregated "small copepod" group; disaggregating this group would significantly reduce the number of observations available for model fitting.

Food web consequences of projected declines

Copepod zooplankton are a key component of the diet of many planktivorous fish, including adults or larval stages of commercially important species, e.g. blue whiting (*Micromesistius poutassou*), herring (*Clupea harengus*) and lesser sandeel (*Ammodytes marinus*) (Conway, 1980; Hillgruber & Kloppmann, 1999; Olin et al., 2022; Prokopchuk, 2009). Variation in copepod zooplankton abundance has been linked with fish recruitment, abundance and marine survival (e.g. Beaugrand et al., 2003; Beaugrand & Reid, 2003; MacDonald et al., 2018; Mills et al., 2013; Olin et al., 2022; Tyldesley et al., 2024). Modelling studies suggest that reduced copepod zooplankton abundance results in a lower "carrying capacity" for planktivorous fish production (Petrik et al., 2022; Richardson, 2008). In turn, reduced forage fish abundance has been negatively associated with diet composition, survival, growth and breeding success of higher predators such as seabirds (Frederiksen et al., 2013; Furness & Tasker, 2000; Régnier et al., 2024; Searle et al., 2023; Wanless et al., 2005, 2018), Atlantic salmon (*Salmo salar*) (Hellenbrecht et al., 2020; Tyldesley et al., 2024; Utne et al., 2021, 2022; Vollset et al., 2022) and other predatory fish (Engelhard et al., 2013), and marine mammals (MacLeod et al., 2007; Trites, 2021). A major climate-driven decline in copepod zooplankton could therefore have negative consequences for forage fish, higher predators and stocks of commercial fisheries.

Our study focused on copepod zooplankton, which forms one albeit sizeable and carbon-rich component of forage fish diet. Other zooplankton observed in forage fish diets include euphausiids, amphipods, polychaetes and Appendicularia (e.g. Dalpadado, 2000; Gómez García et al., 2012; Olin et al., 2022). Some of these have also declined in abundance over past decades while others have opposite or unknown trends (Bedford et al., 2020; Edwards et al., 2021; Holland et al., 2023; Tyldesley et al., 2024). Climate change is expected to have differential impacts on zooplankton classes (e.g. Heneghan et al., 2020). The wider ecological impact of a restructured zooplankton assemblage resulting from climate change would depend on whether the prevailing species could support existing food webs, in terms of energy transfer and predator foraging adaptability. For example, climate change is expected to favour Appendicularia, a group of gelatinous zooplankton, which is important prey for many fish species (Heneghan et al., 2020; Winder et al., 2017); Appendicularia have fast growth rates and a carbon content similar to copepods so have the potential to buffer existing food webs from declines in the currently dominant zooplankton (Jaspers et al., 2023). However, Appendicularia may not be suitable alternative prey: they are patchily distributed, less lipid-rich and harder to handle than copepods, and their high predator to prey size feeding ratio and rapid turnover of mucus feeding "houses" can lead to high carbon export instead of efficient transfer of energy to fish and higher trophic levels (Schmidt et al., 2020; Winder et al., 2017).

Conclusions

This study shows the potential for dramatic declines in copepod zooplankton abundance in the Northeast Atlantic with future climate change. Such declines could reduce the robustness of ecosystems supporting higher predators such as seabirds, predatory fish and cetaceans as well as many commercial fisheries. Against the backdrop of this "shifting baseline", the Northeast Atlantic is subject to expanding anthropogenic pressures (e.g. increasing offshore wind, BEIS, 2022). Increasingly, there is a need to ensure that marine spatial planning and consenting is resilient to climate change (Queirós et al., 2023; Searle et al., 2023). The results of this study imply that environmental impact assessment and conservation measures will need to be robust to potential negative effects of declining zooplankton on the wider Northeast Atlantic ecosystem.

METHODS

Analysis was carried out using R (R Core Team, 2023), implemented through R Studio (Posit Team, 2025), and the MATLAB numerical computing environment (The Mathworks Inc, 2021). Maps were generated using the MATLAB packages *cmocean* (Thyng et al., 2016) and *M_Map* (Pawlowicz, 2020).

Model building

Predictive models of zooplankton variability were generated here using Random Forest (RF) ensemble machine learning (Breiman, 2001). RF generates predictions by aggregating a "forest" of decision trees each trained on a random subset of the data and using a random subset of the explanatory variables (hereafter, variables) at each tree node. RF is non-parametric, i.e. no assumption is made about the shape of the relationship between variables and response. RF is widely used because it has high predictive power, flexibility to data types, avoids overfitting and provides a ranking of variable importance (Genuer & Poggi, 2020). Variable importance is calculated as the mean increase in predictive error resulting from random permutation of each variable. Model prediction error is calculated as the mean out-of-bag error, i.e. predicting on observations outside the training subset for each tree.

Modelling was conducted using the R package *randomForest* (Liaw & Wiener, 2002; R Core Team, 2023). Projecting distributions of plankton, which are patchily distributed and interact with a dynamic and changing physical and biological environment, has been shown to be inherently challenging (Brun et al., 2016; Soley-Guardia et al., 2024). With the aim of gaining ecological insight and building models with high predictive capacity, variables were selected that were likely to be proximal, functional influences on zooplankton variability (Elith & Leathwick, 2009). Latitude and longitude were excluded because strongly geographical variables can result in a model with low error but poor predictive capacity for unsampled areas (Meyer et al., 2019). Variables were checked for pairwise collinearity prior to inclusion in model fitting.

The RF method of averaging over many predictions tends to produce systematic bias whereby the sum of errors is near zero but high response values are underestimated and low values are overestimated, i.e. the slope of predicted on observed values is less than one (Zhang & Lu, 2012). In our case, this could give, for example, overestimated winter zooplankton abundance and reduced amplitude spring blooms. We corrected for this by fitting a standard linear regression of observations on predictions and using the coefficients to calculate adjusted (tilted) predictions for each zooplankton class (Belitz & Stackelberg, 2021; Song, 2015).

The model was checked for sensitivity to the RF input parameters. The number of trees in the ensemble was set to 500, by which the model error was found to stabilise for all zooplankton groups indicating a sufficiently large forest. The number of variables to use at each tree node,

mtry, was varied from 1 to n_p where n_p=12 was the total number of variables; models were subsequently run using the value of that minimised model error: 4, 7 and 8 for small copepods, *C. finmarchicus* and *C. helgolandicus*, respectively. Ten replicates of the RF models were run for each zooplankton class to check the sensitivity of the resulting variable importance ranking.

The models were tested for their ability to extrapolate in space and time. This was required because of the temporally and spatially patchy coverage of the CPR sampling and because projecting a model trained on historical conditions is likely to involve predicting on novel variable ranges, combinations or relationships with the response (Soley-Guardia et al., 2024). Following best practice for spatiotemporal ecological modelling, we used a block cross-validation approach (Roberts et al., 2017). This splits the data into *k* spatial or temporal blocks, trains the RF models *k* times using the data from all but one block and then assesses the ability of the model to predict on the left-out block. Strategic blocking is preferable to random subsetting because autocorrelation prevents such subsets from being statistically independent and can lead to an overestimation of extrapolation ability (Meyer et al., 2019; Roberts et al., 2017; Soley-Guardia et al., 2024). Here we divided the domain into eight contiguous spatial blocks and three temporal blocks (Hastie et al., 2009) (see Supplementary Information). The predictive accuracy of the models on the withheld data was measured using root-mean-square error (RMSE) and the coefficient of determination (R^2). Additionally, the variable importance ranking was compared between time periods to check the temporal stability of their influence on zooplankton energy.

We calculated which regions of the model domain would become "novel predictor space" under each climate projection and therefore require extrapolation by the machine learning models. This was calculated univariately and globally, i.e. by comparing the projections for each environmental variable at each grid-cell with the training data range for the whole model domain. These were combined over environmental variables to obtain a map showing where at least one variable would be outside the training range under each climate projection (Supplementary Information).

To assess the ecological relevance of the trained models, we compared the modelled historical spatial distributions and the relationships between each predictor and zooplankton class (partial distributions) with existing ecological knowledge.

Past zooplankton variability

Models were developed for small copepods (*Centropages* spp., *Acartia* spp., *Oithona* spp., *Para-Pseudocalanus* spp., *Temora longicornis* and juvenile *Calanus* spp.) modelled in aggregate, and the dominant large calanoid copepod species *C. finmarchicus* and *C. helgolandicus,* modelled separately because of their distinct spatial and ecological niches.

Abundance of each species was derived from Continuous Plankton Recorder (CPR) sampling from 1998-2019 over the area 20°W to 3°E and 40°N to 65°N to match the temporal and spatial coverage of the ocean model data. The CPR survey is a large-scale, long-term marine sampling program collecting plankton on reels of silk towed at approximately 10 m water depth by ships of opportunity (Richardson 2006). Sample counts were corrected for the CPR's variability in sampling efficiency for different taxa (Clark et al., 2001; Kane, 2009) by comparing with independent plankton time series from Stonehaven on the Scottish east coast (Bresnan et al., 2015) and the L4 station on the English south coast (Atkinson et al., 2019). Diel vertical migration was accounted for by applying separate factors for samples collected during day and night. Abundance (count) was converted to energy density ($kJ.m^{-3}$) using taxon-specific wet weight and energy density. No correction was attempted for variation in energy content with season and year. See Olin et al. (2022) for full details.

Predictors of past variability in zooplankton

Predictors were derived from the Atlantic Margin Model 3D coupled physical–biogeochemical ocean model reanalysis (1993–2023) for the northwest European shelf at ~7 km horizontal resolution (AMM7) (Copernicus Marine Service; Tonani and Ascione 2021). Each CPR sample was matched with predictors from the closest horizontal grid-cell and time step.

Predictors included in the models were yearday, bathymetric depth (m), daily sea surface temperature (SST), chlorophyll integrated over 0-100 m water depth, mixed layer depth (m) and sea surface salinity, monthly SST anomaly with respect to the 1993-2023 climatology, monthly nitrate anomaly over 0-100 m depth with respect to the same period and annual mean winter nitrate concentration (January-March, i.e. prior to the phytoplankton spring bloom).

In addition, we included a measure of sub-polar gyre (SPG) strength: annual mean upper ocean (50-500 m) salinity anomaly at the model grid-cell having strongest historical correlation between upper ocean salinity and an SPG index time series (Chafik 2019; Pearson's R=-0.7 at 12.1W 54.1N over 1993-2018) (see Supplementary Information). The reason for including this proxy rather than the SPG index itself was to retain SPG strength when applying the models of zooplankton variability into the future, because although projections of the SPG strength do not exist, this same measure could be readily calculated in the climate projections. Including the SPG strength is important because it regulates the relative influences of subpolar and subtropical water masses and exerts bottom-up control on zooplankton assemblages in the Northeast Atlantic (e.g. Hátún et al., 2016). A complimentary index of relative Arctic water influences in the Norwegian Sea (Skagseth et al., 2022) was initially included in the model but subsequently removed due to high collinearity with the SPG proxy.

Climate projections

Projections of future ocean conditions were taken from an ensemble of three coupled hydrodynamic-ecosystem simulations. Each simulation implemented NEMO-ERSEM on the AMM7 domain under the Intergovernmental Panel on Climate Change (IPCC) Representative Concentration Pathway "high emissions" scenario RCP8.5. The simulations differed in vertical resolution, NEMO version, ERSEM parameterisation, temporal resolution of output and which of the IPCC's Coupled Model Intercomparison Project Phase 5 (CMIP5) global Earth System Model (ESM) was used for atmospheric and oceanic boundary forcing (Taylor et al., 2012) (Table 1).

The reanalysis and projections each used a different definition of mixed layer depth. To ensure the predictors were equivalent in the training and projection models, MLD was therefore recalculated in the projections according to the definition used in the reanalysis: the depth at which the density increase compared to density at 3 m depth corresponds to a temperature decrease of 0.2°C in surface conditions (Tonani & Ascione, 2021).

Model output was at daily and monthly resolution. ROAM-HADGEM2 biogeochemical output was only available at monthly resolution, preventing calculation of phytoplankton bloom phenology for this model. Therefore, "reduced" RF models excluding bloom start and duration were used for zooplankton projections under this climate projection.

Bias correction of climate projections

These long-term simulations project future trends in climate change; the timings of their internal variations are not expected to match observational records or future events (Taylor et al., 2012). In addition, all free-running climate projections exhibit bias, whereby they systematically deviate from mean historical observations due to imperfect representation of initial conditions, forcings and internal processes (Fung, 2018; Maraun, 2016). It is therefore standard practice to apply a

bias correction before using the output (Fung, 2018; Navarro-Racines et al., 2020; Teutschbein & Seibert, 2012). Here we used the "Delta change" method, which calculates the difference (Delta) in mean conditions between a reference historical and future period(s) in each projection and then applies this as a perturbation to a recent reference period in the reanalysis output (e.g. Maraun, 2016). This treats the reanalysis as a proxy for observations, which is reasonable because the model has been validated and assimilates satellite ocean colour and SST (Kay et al., 2021; Renshaw et al., 2021). Deltas are calculated multiplicatively for predictors that can only take positive values (e.g. chlorophyll concentration) to avoid negative bias-corrected values, and additively otherwise (e.g. Navarro-Racines et al., 2020). Since the climate change signal will vary seasonally and regionally (e.g. Teutschbein & Seibert, 2012), we calculate the Delta change values by month (except for annual metrics such as bloom phenology), and by model grid-cell. The monthly Deltas are then interpolated to daily resolution and applied as a "climate change perturbation" to each year of the reanalysis reference period for the recent past (2000-2019). This obtains a bias-corrected future time series that preserves the climate change signal and realistic variability over a range of scales (Alexander et al., 2020). We apply this method for two future periods: 2040-2049 (near future) and 2090-2099 (far future). The RF models are then predicted on these time series to obtain zooplankton future projections for each zooplankton class and climate projection model.

DATA AVAILABILITY

The Continuous Plankton Recorder data are available from the Marine Biological Association at https://doi.dassh.ac.uk/data/1850 and https://doi.mba.ac.uk/data/3118.

The regional ocean physical-biogeochemical model reanalysis data are available through Copernicus Marine Service (https://data.marine.copernicus.eu), products NWSHELF_MULTIYEAR_PHY_004_009 and NWSHELF_MULTIYEAR_BGC_004_011.

The regional physical-biogeochemical model projection data are available through the British Oceanographic Data Centre at https://doi.org/10.5285/07877700-0e22-5fb5-e063-6c86abc03058 and https://doi.org/10.5285/0786d770-ba54-0e57-e063-6c86abc09fdf, and through Zendo at https://zenodo.org/records/3953801.

CODE AVAILABILITY

The code developed for this analysis is available at: https://github.com/EmmaTyldesley/zoo_projections_train_RF.

[Note: upon acceptance of the manuscript, the data in this GitHub repository would be transferred to a PURE Dataset and a DOI issued ahead of publication.]

REFERENCES

Alexander, M. A., Shin, S. I., Scott, J. D., Curchitser, E., & Stock, C. (2020). The response of the Northwest Atlantic Ocean to climate change. *Journal of Climate*, *33*(2), 405–428. https://doi.org/10.1175/JCLI-D-19-0117.1

Atkinson, A., McEvoy, A., & Lilley, M. (2019). Plymouth L4 time series (1988-2017) of zooplankton abundance, biomass and traits. *British Oceanographic Data Centre, National Oceanography Centre, National Environmental Research Council (NERC), UK*. https://doi.org/10.5285/827b5179-90a1-69d8-e053-6c86abc06f55

Banas, N. S., Møller, E. F., Nielsen, T. G., & Eisner, L. B. (2016). Copepod life strategy and population viability in response to prey timing and temperature: Testing a new model across latitude, time, and the size spectrum. *Frontiers in Marine Science*, *3*(NOV), 1–21. https://doi.org/10.3389/fmars.2016.00225

Banas, N. S., Wilson, R., Heath, M. R., & Speirs, D. C. (2016). Projected impacts of 21st century climate change on diapause in Calanus finmarchicus. *Global Change Biology*, *22*(10), 3332–3340. https://doi.org/10.1111/gcb.13282

Beaugrand, G., Brander, K. M., Lindley, J. A., Souissi, S., & Reid, P. C. (2003). Plankton effect on cod recruitment in the North Sea. *Nature*, *426*(6967), 661–664. https://doi.org/10.1038/nature02164

Beaugrand, G., Christophe, L., & Edwards, M. (2009). Rapid biogeographical plankton shifts in the North Atlantic Ocean. *Global Change Biology*, *15*(7), 1790–1803. https://doi.org/10.1111/j.1365-2486.2009.01848.x

Beaugrand, G., & Reid, P. C. (2003). Long-term changes in phytoplankton, zooplankton and salmon related to climate. *Global Change Biology*, *9*(6), 801–817. https://doi.org/10.1046/j.1365-2486.2003.00632.x

Beaugrand, G., & Reid, P. C. (2013). Relationships between North Atlantic salmon, plankton, and hydroclimatic change in the Northeast Atlantic. *ICES Journal of Marine Science*, *69*(10), 1549–1562. https://doi.org/10.1038/278097a0

Bedford, J., Ostle, C., Johns, D. G., Atkinson, A., Best, M., Bresnan, E., Machairopoulou, M., Graves, C. A., Devlin, M., Milligan, A., Pitois, S., Mellor, A., Tett, P., & McQuatters-Gollop, A. (2020). Lifeform indicators reveal large-scale shifts in plankton across the North-West European shelf. *Global Change Biology*, *26*(6), 3482–3497. https://doi.org/10.1111/gcb.15066

BEIS. (2022). *British energy security strategy* (Policy Paper). https://www.gov.uk/government/publications/british-energy-security-strategy/british-energy-security-strategy

Belitz, K., & Stackelberg, P. E. (2021). Evaluation of six methods for correcting bias in estimates from ensemble tree machine learning regression models. *Environmental Modelling and Software*, *139*(February), 105006–105006. https://doi.org/10.1016/j.envsoft.2021.105006

Bonnet, D., Richardson, A., Harris, R., Hirst, A., Beaugrand, G., Edwards, M., Ceballos, S., Diekman, R., López-Urrutia, A., Valdes, L., Carlotti, F., Molinero, J. C., Weikert, H., Greve, W., Lucic, D., Albaina, A., Yahia, N. D., Umani, S. F., Miranda, A., … Fernandez De Puelles, M. L. (2005). An overview of Calanus helgolandicus ecology in European waters. *Progress in Oceanography*, *65*(1), 1–53. https://doi.org/10.1016/j.pocean.2005.02.002

Breiman, L. (2001). Random Forests. *Machine Learning*, *45*(1), 5–32. https://doi.org/10.1023/A:1010933404324

Bresnan, E., Cook, K. B., Hughes, S. L., Hay, S. J., Smith, K., Walsham, P., & Webster, L. (2015). Seasonality of the plankton community at an east and west coast monitoring site in Scottish waters. *Journal of Sea Research*, *105*(June), 16–29. https://doi.org/10.1016/j.seares.2015.06.009

Brun, P., Kiørboe, T., Licandro, P., & Payne, M. R. (2016). The predictive skill of species distribution models for plankton in a changing climate. *Global Change Biology*, *22*(9), 3170–3181. https://doi.org/10.1111/gcb.13274

Chafik, L. (2019). North Atlantic subpolar gyre index. Dataset version 3.0. Https://doi.org/10.17043/chafik-2019-3 [Dataset]. In *Bolin Centre Database*. https://doi.org/10.17043/chafik-2019-3

Choquet, M., Hatlebakk, M., Dhanasiri, A. K. S., Kosobokova, K., Smolina, I., Søreide, J. E., Svensen, C., Melle, W., Kwaśniewski, S., Eiane, K., Daase, M., Tverberg, V., Skreslet, S., Bucklin, A., & Hoarau, G. (2017). Genetics redraws pelagic biogeography of Calanus. *Biology Letters*, *13*(12), 20170588. https://doi.org/10.1098/rsbl.2017.0588

Chust, G., Castellani, C., Licandro, P., Ibaibarriaga, L., Sagarminaga, Y., & Irigoien, X. (2014). Are Calanus spp. shifting poleward in the North Atlantic? A habitat modelling approach. *ICES Journal of Marine Science*, *71*(2), 241–253. https://doi.org/10.1093/icesjms/fst147

Clark, R. A., Frid, C. L. J., & Batten, S. (2001). A critical comparison of two long-term zooplankton time series from the central-west North Sea. *Journal of Plankton Research*, *23*(1), 27–39. https://doi.org/10.1093/plankt/23.1.27

Continuous Plankton Recorder Survey Team. (2004). Continuous Plankton Records: Plankton Atlas of the North Atlantic Ocean (1958–1999). II. Biogeographical charts. *Marine Ecology Progress Series*, 11–75.

Conway, D. V. P. (1980). The food of larval blue whiting, Micromesistius poutassou (Risso), in the Rockall area. *Journal of Fish Biology*, *16*(6), 709–723. https://doi.org/10.1111/j.1095-8649.1980.tb03750.x

Corona, S., Hirst, A. G., Atkinson, D., Renz, J., Boersma, M., & Atkinson, A. (2024). Long-term shifts in phenology, thermal niche, population size, and their interactions in marine pelagic copepods. *Limnology and Oceanography*, *69*(3), 482–497. https://doi.org/10.1002/lno.12499

Daewel, U., Hjøllo, S. S., Huret, M., Ji, R., Maar, M., Niiranen, S., Travers-Trolet, M., Peck, M. A., & van de Wolfshaar, K. E. (2014). Predation control of zooplankton dynamics: A review of observations and models. *ICES Journal of Marine Science*, *71*(2), 254–271. https://doi.org/10.1093/icesjms/fst125

Dalpadado, P. (2000). Food and feeding conditions of Norwegian spring-spawning herring (Clupea harengus) through its feeding migrations. *ICES Journal of Marine Science*, *57*(4), 843–857. https://doi.org/10.1006/jmsc.2000.0573

Dalpadado, P., Prokopchuk, I. P., Bogstad, B., Skaret, G., Ingvaldsen, R. B., Dolgov, A. V., Boyko, A. S., Rey, A., Ono, K., Bagøien, E., & Huse, G. (2024). Zooplankton link climate to capelin and polar cod in the Barents Sea. *Progress in Oceanography*, *226*, 103302. https://doi.org/10.1016/j.pocean.2024.103302

Daufresne, M., Lengfellner, K., & Sommer, U. (2009). Global warming benefits the small in aquatic ecosystems. *Proceedings of the National Academy of Sciences*, *106*(31), 12788–12793. https://doi.org/10.1073/pnas.0902080106

Drenkard, E. J., Stock, C., Ross, A. C., Dixon, K. W., Adcroft, A., Alexander, M., Balaji, V., Bograd, S. J., Butenschön, M., Cheng, W., Curchitser, E., Lorenzo, E. D., Dussin, R., Haynie, A. C., Harrison, M., Hermann, A., Hollowed, A., Holsman, K., Holt, J., … Wang, M. (2021). Next-generation regional ocean projections for living marine resource management in a changing climate. *ICES Journal of Marine Science*, *78*(6), 1969–1987. https://doi.org/10.1093/icesjms/fsab100

Eddy, T. D., Heneghan, R. F., Bryndum-Buchholz, A., Fulton, E. A., Harrison, C. S., Tittensor, D. P., Lotze, H. K., Ortega-Cisneros, K., Novaglio, C., Bianchi, D., Büchner, M., Bulman, C., Cheung, W. W. L., Christensen, V., Coll, M., Everett, J. D., Fierro-Arcos, D., Galbraith, E. D., Gascuel, D., … Blanchard, J. L. (2025). Global and Regional Marine Ecosystem Models Reveal Key Uncertainties in Climate Change Projections. *Earth's Future*, *13*(3), e2024EF005537. https://doi.org/10.1029/2024EF005537

Edwards, M., Bresnan, E., Cook, K., Heath, M., Helanaouet, P., Lynam, C., Raine, R., & Widdicombe, C. (2013). Impacts of climate change on plankton [Pdf]. *MCCIP Science Review 2013*, 15 pages. https://doi.org/10.14465/2013.ARC12.098-112

Edwards, M., Hélaouët, P., Goberville, E., Lindley, A., Tarling, G. A., Burrows, M. T., & Atkinson, A. (2021). North Atlantic warming over six decades drives decreases in krill abundance with no associated range shift. *Communications Biology*, *4*(1), 1–10. https://doi.org/10.1038/s42003-021-02159-1

Elith, J., & Leathwick, J. R. (2009). Species distribution models: Ecological explanation and prediction across space and time. *Annual Review of Ecology, Evolution, and Systematics*, *40*, 677–697. https://doi.org/10.1146/annurev.ecolsys.110308.120159

Engelhard, G. H., Blanchard, J. L., Pinnegar, J. K., van der Kooij, J., Bell, E. D., Mackinson, S., & Righton, D. A. (2013). Body condition of predatory fishes linked to the availability of sandeels. *Marine Biology*, *160*(2), 299–308. https://doi.org/10.1007/s00227-012-2088-1

E.U. Copernicus Marine Service Information (CMEMS), Marine Data Store (MDS). (2025a). *Atlantic- European North West Shelf- Ocean Biogeochemistry Reanalysis* [Dataset]. https://doi.org/10.48670/moi-00058

E.U. Copernicus Marine Service Information (CMEMS), Marine Data Store (MDS). (2025b). *Atlantic- European North West Shelf- Ocean Physics Reanalysis* [Dataset]. https://doi.org/10.48670/moi-00059

Fanjul, A., Iriarte, A., Villate, F., Uriarte, I., Atkinson, A., & Cook, K. (2018). Zooplankton seasonality across a latitudinal gradient in the Northeast Atlantic Shelves Province. *Continental Shelf Research*, *160*, 49–62. https://doi.org/10.1016/j.csr.2018.03.009

Fauchald, P., Skov, H., Skern-Mauritzen, M., Johns, D., & Tveraa, T. (2011). Wasp-Waist Interactions in the North Sea Ecosystem. *PLOS ONE*, *6*(7), e22729. https://doi.org/10.1371/journal.pone.0022729

Frederiksen, M., Anker-Nilssen, T., Beaugrand, G., & Wanless, S. (2013). Climate, copepods and seabirds in the boreal Northeast Atlantic—Current state and future outlook. *Global Change Biology*, *19*(2), 364–372. https://doi.org/10.1111/gcb.12072

Frederiksen, M., Edwards, M., Richardson, A. J., Halliday, N. C., & Wanless, S. (2006). From plankton to top predators: Bottom-up control of a marine food web across four trophic levels. *Journal of Animal Ecology*, *75*(6), 1259–1268. https://doi.org/10.1111/j.1365-2656.2006.01148.x

Friedland, K. D., Record, N. R., Asch, R. G., Kristiansen, T., Saba, V. S., Drinkwater, K. F., Henson, S., Leaf, R. T., Morse, R. E., Johns, D. G., Large, S. I., Hjøllo, S. S., Nye, J. A., Alexander, M. A., & Ji, R. (2016). Seasonal phytoplankton blooms in the North Atlantic linked to the overwintering strategies of copepods. *Elementa: Science of the Anthropocene*, *4*, 000099. https://doi.org/10.12952/journal.elementa.000099

Fung, F. (2018). *Bias Correction* (UKCP18 Guidance).

Furness, R. W., & Tasker, M. L. (2000). Seabird-fishery interactions: Quantifying the sensitivity of seabirds to reductions in sandeel abundance, and identification of key areas for sensitive seabirds in the North Sea. *Marine Ecology Progress Series*, *202*, 253–264. https://doi.org/10.3354/meps202253

Galli, G., Artioli, Y., Wakelin, S., Harle, J., & Holt, J. (2023a). *Northwest European Shelf daily and monthly physical-biogeochemical data (1990-2099) from NEMO-ERSEM coupled physics-biogeochemistry climate model forced by GFDL-ESM2G under scenario RCP8.5*. [Dataset]. https://doi.org/10.5285/07877700-0e22-5fb5-e063-6c86abc03058

Galli, G., Artioli, Y., Wakelin, S., Harle, J., & Holt, J. (2023b). *Northwest European Shelf daily and monthly physical-biogeochemical data (1990-2099) from NEMO-ERSEM coupled physics-biogeochemistry climate model forced by IPSL-CM5A-MR under scenario RCP8.5*. [Dataset]. NERC EDS British Oceanographic Data Centre NOC. https://doi.org/10.5285/0786d770-ba54-0e57-e063-6c86abc09fdf

Gentleman, W., Leising, A., Frost, B., Strom, S., & Murray, J. (2003). Functional responses for zooplankton feeding on multiple resources: A review of assumptions and biological dynamics. *Deep Sea Research Part II: Topical Studies in Oceanography*, *50*(22), 2847–2875. https://doi.org/10.1016/j.dsr2.2003.07.001

Genuer, R., & Poggi, J.-M. (2020). *Random Forests with R*. Springer International Publishing. https://doi.org/10.1007/978-3-030-56485-8

Gómez García, G., Demain, D., Drewery, H., & Gallego, A. (2012). *The Distribution of Zooplankton Prey of Forage Fish in the Firth of Forth Area, East Coast of Scotland* (No. Vol 3 No 4; Scottish Marine and Freshwater Science Report).

Grieve, B. D., Hare, J. A., & Saba, V. S. (2017). Projecting the effects of climate change on Calanus finmarchicus distribution within the U.S. Northeast Continental Shelf. *Scientific Reports*, *7*(1), 6264. https://doi.org/10.1038/s41598-017-06524-1

Hastie, T., Tibshirani, R., & Friedman, J. (2009). *The Elements of Statistical Learning*. Springer. https://doi.org/10.1007/978-0-387-84858-7

Hátún, H., & Chafik, L. (2018). On the Recent Ambiguity of the North Atlantic Subpolar Gyre Index. *Journal of Geophysical Research: Oceans*, *123*(8), 5072–5076. https://doi.org/10.1029/2018JC014101

Hátún, H., Lohmann, K., Matei, D., Jungclaus, J. H., Pacariz, S., Bersch, M., Gislason, A., Ólafsson, J., & Reid, P. C. (2016). An inflated subpolar gyre blows life toward the northeastern Atlantic. *Progress in Oceanography*, *147*, 49–66. https://doi.org/10.1016/j.pocean.2016.07.009

Hátún, H., Payne, M. R., Beaugrand, G., Reid, P. C., Sandø, A. B., Drange, H., Hansen, B., Jacobsen, J. A., & Bloch, D. (2009). Large bio-geographical shifts in the north-eastern Atlantic Ocean: From the subpolar gyre, via plankton, to blue whiting and pilot whales. *Progress in Oceanography*, *80*(3–4), 149–162. https://doi.org/10.1016/j.pocean.2009.03.001

Hausfather, Z., & Peters, G. P. (2020). Emissions – the 'business as usual' story is misleading. *Nature*, *577*(7792), 618–620. https://doi.org/10.1038/d41586-020-00177-3

Heath, M. R. (2007). The consumption of zooplankton by early life stages of fish in the North Sea. *ICES Journal of Marine Science*, *64*(9), 1650–1663. https://doi.org/10.1093/icesjms/fsm156

Helaouët, P., & Beaugrand, G. (2007). Macroecology of Calanus finmarchicus and C. helgolandicus in the North Atlantic Ocean and adjacent seas. *Marine Ecology Progress Series*, *345*, 147–165. https://doi.org/10.3354/meps06775

Hellenbrecht, L. M., Wennevik, V., Glover, K. A., & Utne, K. R. (2020). *Feeding of wild Atlantic salmon (Salmo salar) post-smolts in four Norwegian fjords*. *July*.

Heneghan, R. F., Everett, J. D., Blanchard, J. L., Sykes, P., & Richardson, A. J. (2023). Climate-driven zooplankton shifts cause large-scale declines in food quality for fish. *Nature Climate Change*, *13*(5), 470–477. https://doi.org/10.1038/s41558-023-01630-7

Heneghan, R. F., Everett, J. D., Sykes, P., Batten, S. D., Edwards, M., Takahashi, K., Suthers, I. M., Blanchard, J. L., & Richardson, A. J. (2020). A functional size-spectrum model of the global marine ecosystem that resolves zooplankton composition. *Ecological Modelling*, *435*, 109265. https://doi.org/10.1016/j.ecolmodel.2020.109265

Hillgruber, N., & Kloppmann, M. (1999). Distribution and feeding of blue whiting Micromesistius poutassou larvae in relation to different water masses in the Porcupine Bank area, west of Ireland. *Marine Ecology Progress Series*, *187*(1996), 213–225. https://doi.org/10.3354/meps187213

Hinder, S. L., Gravenor, M. B., Edwards, M., Ostle, C., Bodger, O. G., Lee, P. L. M., Walne, A. W., & Hays, G. C. (2014). Multi-decadal range changes vs. Thermal adaptation for north east Atlantic oceanic copepods in the face of climate change. *Global Change Biology*, *20*(1), 140–146. https://doi.org/10.1111/gcb.12387

Holland, M. M., Louchart, A., Artigas, L. F., Ostle, C., Atkinson, A., Rombouts, I., Graves, C. A., Devlin, M., Heyden, B., Machairopoulou, M., Bresnan, E., Schilder, J., Jakobsen, H. H., Llody-Hartley, H., Tett, P., Best, M., Goberville, E., & McQuatters-Gollop, A. (2023). Major declines in NE Atlantic plankton contrast with more stable populations in the rapidly warming North Sea. *Science of the Total Environment*, *898*(June), 165505–165505. https://doi.org/10.1016/j.scitotenv.2023.165505

Holt, J., Hyder, P., Ashworth, M., Harle, J., Hewitt, H. T., Liu, H., New, A. L., Pickles, S., Porter, A., Popova, E., Allen, J. I., Siddorn, J., & Wood, R. (2017). Prospects for improving the representation of coastal and shelf seas in global ocean models. *Geoscientific Model Development*, *10*(1), 499–523. https://doi.org/10.5194/gmd-10-499-2017

Holt, J., Polton, J., Huthnance, J., Wakelin, S., O'Dea, E., Harle, J., Yool, A., Artioli, Y., Blackford, J., Siddorn, J., & Inall, M. (2018). Climate-Driven Change in the North Atlantic and Arctic Oceans Can Greatly Reduce the Circulation of the North Sea. *Geophysical Research Letters*, *45*(21), 11,827-11,836. https://doi.org/10.1029/2018GL078878

IPCC. (2013). *Climate Change 2013: The Physical Science Basis. Contribution of Working Group I to the Fifth Assessment Report of the Intergovernmental Panel on Climate Change* (F. T. Stocker, D. Qin, G. K. Plattner, M. Tigmor, S. K. Allen, J. Boschung, A. Nauels, Y. Xia, V. Bex, & P. M. Midgley, Eds.). Cambridge University Press.

IPCC. (2021). *Climate Change 2021: The Physical Science Basis. Contribution of Working Group I to the Sixth Assessment Report of the Intergovernmental Panel on Climate Change* (V. Masson-Delmotte, A. Pirani, S. L. Connors, C. Péan, N. Caud, L. Goldfarb, M. I. Gomis, M. Huang, K. Leitzell, E. Lonnoy, J. B. R. Matthews, T. K. Maycock, T. Waterfield, Ö. Yelekçi, R. Yu, & B. Zhou, Eds.). Cambridge University Press. https://doi.org/10.1017/9781009157896

Jaspers, C., Hopcroft, R. R., Kiørboe, T., Lombard, F., López-Urrutia, Á., Everett, J. D., & Richardson, A. J. (2023). Gelatinous larvacean zooplankton can enhance trophic transfer and carbon sequestration. *Trends in Ecology & Evolution*, *38*(10), 980–993. https://doi.org/10.1016/j.tree.2023.05.005

Ji, R., Edwards, M., Mackas, D. L., Runge, J. A., & Thomas, A. C. (2010a). Marine plankton phenology and life history in a changing climate: Current research and future directions. *Journal of Plankton Research*, *32*(10), 1355–1368. https://doi.org/10.1093/plankt/fbq062

Ji, R., Edwards, M., Mackas, D. L., Runge, J. A., & Thomas, A. C. (2010b). Marine plankton phenology and life history in a changing climate: Current research and future directions. *Journal of Plankton Research*, *32*(10), 1355–1368. https://doi.org/10.1093/plankt/fbq062

Jones, C. D., Hughes, J. K., Bellouin, N., Hardiman, S. C., Jones, G. S., Knight, J., Liddicoat, S., O'Connor, F. M., Andres, R. J., Bell, C., Boo, K.-O., Bozzo, A., Butchart, N., Cadule, P., Corbin, K. D., Doutriaux-Boucher, M., Friedlingstein, P., Gornall, J., Gray, L., … Zerroukat, M. (2011). The HadGEM2-ES implementation of CMIP5 centennial simulations. *Geoscientific Model Development*, *4*(3), 543–570. https://doi.org/10.5194/gmd-4-543-2011

Kane, J. (2009). A comparison of two zooplankton time series data collected in the Gulf of Maine. *Journal of Plankton Research*, *31*(3), 249–259. https://doi.org/10.1093/plankt/fbn119

Kara, A. B., Rochford, P. A., & Hurlburt, H. E. (2000). An optimal definition for ocean mixed layer depth. *Journal of Geophysical Research: Oceans*, *105*(C7), 16803–16821. https://doi.org/10.1029/2000jc900072

Kay, S., McEwan, R., & Ford, D. (2021). *Quality Information Document for North West European Shelf Production Centre: NWSHELF_MULTIYEAR_BIO_004_011* (pp. 1–39).

Kjesbu, O. S., Sundby, S., Sandø, A. B., Alix, M., Hjøllo, S. S., Tiedemann, M., Skern-Mauritzen, M., Junge, C., Fossheim, M., Thorsen Broms, C., Søvik, G., Zimmermann, F., Nedreaas, K., Eriksen, E., Höffle, H., Hjelset, A. M., Kvamme, C., Reecht, Y., Knutsen, H., … Huse, G. (2022). Highly mixed impacts of near-future climate change on stock productivity proxies in the North East Atlantic. *Fish and Fisheries*, *23*(3), 601–615. https://doi.org/10.1111/faf.12635

Liaw, A., & Wiener, M. (2002). Classification and Regression by randomForest. *R News*, *2*(3), 18–22.

Lynam, C. P., Llope, M., Möllmann, C., Helaouët, P., Bayliss-Brown, G. A., & Stenseth, N. C. (2017). Interaction between top-down and bottom-up control in marine food webs. *Proceedings of the National Academy of Sciences*, *114*(8), 1952–1957. https://doi.org/10.1073/pnas.1621037114

MacDonald, A., Heath, M., Edwards, M., Furness, R., Pinnegar, J. K., Wanless, S., Speirs, D., & Greenstreet, S. (2015). Climate driven trophic cascades affecting seabirds around the British Isles. *Oceanography and Marine Biology - An Annual Review*, *53*, 55–79. https://doi.org/10.1201/b18733-3

MacDonald, A., Speirs, D. C., Greenstreet, S. P. R., Boulcott, P., & Heath, M. R. (2019). Trends in Sandeel Growth and Abundance off the East Coast of Scotland. *Frontiers in Marine Science*, *6*, 201. https://doi.org/10.3389/fmars.2019.00201

MacDonald, A., Speirs, D. C., Greenstreet, S. P. R., & Heath, M. R. (2018). Exploring the influence of food and temperature on North Sea Sandeels using a new dynamic energy budget model. *Frontiers in Marine Science*, *5*(SEP). https://doi.org/10.3389/fmars.2018.00339

MacLeod, C. D., Santos, M. B., Reid, R. J., Scott, B. E., & Pierce, G. J. (2007). Linking sandeel consumption and the likelihood of starvation in harbour porpoises in the Scottish North Sea: Could climate change mean more starving porpoises? *Biology Letters*, *3*(2), 185–188. https://doi.org/10.1098/rsbl.2006.0588

Maraun, D. (2016). Bias Correcting Climate Change Simulations—A Critical Review. *Current Climate Change Reports*, *2*(4), 211–220. https://doi.org/10.1007/s40641-016-0050-x

Melle, W., Runge, J., Head, E., Plourde, S., Castellani, C., Licandro, P., Pierson, J., Jonasdottir, S., Johnson, C., Broms, C., Debes, H., Falkenhaug, T., Gaard, E., Gislason, A., Heath, M., Niehoff, B., Nielsen, T. G., Pepin, P., Stenevik, E. K., & Chust, G. (2014). The North Atlantic Ocean as habitat for *Calanus finmarchicus*: Environmental factors and life history traits. *Progress in Oceanography*, *129*, 244–284. https://doi.org/10.1016/j.pocean.2014.04.026

Meyer, H., Reudenbach, C., Wöllauer, S., & Nauss, T. (2019). Importance of spatial predictor variable selection in machine learning applications – Moving from data reproduction to spatial prediction. *Ecological Modelling*, *411*, 108815. https://doi.org/10.1016/j.ecolmodel.2019.108815

Mills, K. E., Pershing, A. J., Sheehan, T. F., & Mountain, D. (2013). Climate and ecosystem linkages explain widespread declines in North American Atlantic salmon populations. *Global Change Biology*, *19*(10), 3046–3061. https://doi.org/10.1111/gcb.12298

Mitra, A., Castellani, C., Gentleman, W. C., Jónasdóttir, S. H., Flynn, K. J., Bode, A., Halsband, C., Kuhn, P., Licandro, P., Agersted, M. D., Calbet, A., Lindeque, P. K., Koppelmann, R., Møller, E. F., Gislason, A., Nielsen, T. G., & St. John, M. (2014). Bridging the gap between marine biogeochemical and fisheries sciences; configuring the zooplankton link. *Progress in Oceanography*, *129*, 176–199. https://doi.org/10.1016/j.pocean.2014.04.025

Navarro-Racines, C., Tarapues, J., Thornton, P., Jarvis, A., & Ramirez-Villegas, J. (2020). High-resolution and bias-corrected CMIP5 projections for climate change impact assessments. *Scientific Data*, *7*(1), 7. https://doi.org/10.1038/s41597-019-0343-8

Olin, A. B., Banas, N. S., Johns, D. G., Heath, M. R., Wright, P. J., & Nager, R. G. (2022). Spatio-temporal variation in the zooplankton prey of lesser sandeels: Species and community trait patterns from the Continuous Plankton Recorder. *ICES Journal of Marine Science*, *79*(5), 1649–1661. https://doi.org/10.1093/icesjms/fsac101

Olin, A. B., Banas, N. S., Wright, P. J., Heath, M. R., & Nager, R. G. (2020). Spatial synchrony of breeding success in the black-legged kittiwake Rissa tridactyla reflects the spatial dynamics of its sandeel prey. *Marine Ecology Progress Series*, *638*, 177–190. https://doi.org/10.3354/meps13252

OSPAR. (2023a). *Pelagic Habitats Thematic Assessment*. (Quality Status Report 2023.). https://oap.ospar.org/en/ospar-assessments/quality-status-reports/qsr-2023/

OSPAR. (2023b). *Quality Status Report 2023*. https://oap.ospar.org/en/ospar-assessments/quality-status-reports/qsr-2023/

Pawlowicz, R. (2020). *M_Map: A mapping package for MATLAB* (Version 1.4m) [Computer software]. www.eoas.ubc.ca/~rich/map.html

Petrik, C. M., Luo, J. Y., Heneghan, R. F., Everett, J. D., Harrison, C. S., & Richardson, A. J. (2022). Assessment and Constraint of Mesozooplankton in CMIP6 Earth System Models. *Global Biogeochemical Cycles*, *36*(11), 1–25. https://doi.org/10.1029/2022GB007367

Planque, B., & Fromentin, J.-M. (1996). Calanus and environment in the eastern North Atlantic. I. Spatial and temporal patterns of C. finmarchicus and C. helgolandicus. *Marine Ecology Progress Series*, *134*(1/3), 101–109.

Posit Team. (2025). *RStudio: Integrated Development Environment for R* [Computer software]. Posit Software, PBC. http://www.posit.co/

Prokopchuk, I. (2009). Feeding of the Norwegian spring spawning herring Clupea harengus (Linne) at the different stages of its life cycle. *Deep-Sea Research Part II: Topical Studies in Oceanography*, *56*(21–22), 2044–2053. https://doi.org/10.1016/j.dsr2.2008.11.015

Queirós, A. M., Kay, S., Sciberras, M., Talbot, E., Kaiser, M., Wilson, R. J., Sailley, S., Marra, S., Matear, L., Fernandes, J., Aldridge, J., McEwan, R., Morris, K., McNeil, C. L., Nunes, J., Woodcock, K., Duncombe-Smith, S., Smith, A., Lynam, C., … Somerfield, P. J. (2023). *Early-warning system: Climate-smart spatial management of UK fisheries, aquaculture and conservation* (A Report of the NERC/ESRC Marine Spatial Planning Addressing Climate Effects Project, p. 58). 10.14465/2023.msp02.tec

R Core Team. (2023). *R: A Language and Environment for Statistical Computing. R Foundation for Statistical Computing, Vienna, Austria. Https://www.r-project.org/* [Computer software]. https://www.r-project.org/

Ratnarajah, L., Abu-Alhaija, R., Atkinson, A., Batten, S., Bax, N. J., Bernard, K. S., Canonico, G., Cornils, A., Everett, J. D., Grigoratou, M., Ishak, N. H. A., Johns, D., Lombard, F., Muxagata, E., Ostle, C., Pitois, S., Richardson, A. J., Schmidt, K., Stemmann, L., … Yebra, L. (2023). Monitoring and modelling marine zooplankton in a changing climate. *Nature Communications*, *14*(1). https://doi.org/10.1038/s41467-023-36241-5

Record, N. R., Ji, R., Varpe, Ø., Runge, J., Petrik, C. M., & Johns, D. (2018). Copepod diapause and the biogeography of the marine lipidscape. *Journal of Biogeography*, *45*, 2238–2251. https://doi.org/10.1111/jbi.13414

Régnier, T., Wright, P. J., Harris, M. P., Gibb, F. M., Newell, M., Eerkes-Medrano, D., Daunt, F., & Wanless, S. (2024). Effect of timing and abundance of lesser sandeel on the breeding success of a North Sea seabird community. *Marine Ecology Progress Series*, *727*, 1–17. https://doi.org/10.3354/meps14520

Renshaw, R., Wakelin, S., Golbeck, I., & O'Dea, E. (2021). *Quality Information Document for North West European Shelf Production Centre NWSHELF_MULTIYEAR_PHY_004_009* (pp. 1–39). https://resources.marine.copernicus.eu/documents/QUID/CMEMS-NWS-QUID-004-009.pdf

Reygondeau, G., & Beaugrand, G. (2011). Future climate-driven shifts in distribution of Calanus finmarchicus. *Global Change Biology*, *17*(2), 756–766. https://doi.org/10.1111/j.1365-2486.2010.02310.x

Richardson, A. J. (2008). In hot water: Zooplankton and climate change. *ICES Journal of Marine Science*, *65*(3), 279–295. https://doi.org/10.1093/icesjms/fsn028

Richardson, A. J., Walne, A. W., John, A. W. G., Jonas, T. D., Lindley, J. A., Sims, D. W., Stevens, D., & Witt, M. (2006). Using continuous plankton recorder data. *Progress in Oceanography*, *68*(1), 27–74. https://doi.org/10.1016/j.pocean.2005.09.011

Roberts, D. R., Bahn, V., Ciuti, S., Boyce, M. S., Elith, J., Guillera-Arroita, G., Hauenstein, S., Lahoz-Monfort, J. J., Schröder, B., Thuiller, W., Warton, D. I., Wintle, B. A., Hartig, F., & Dormann, C. F. (2017). Cross-validation strategies for data with temporal, spatial, hierarchical, or phylogenetic structure. *Ecography*, *40*(8), 913–929. https://doi.org/10.1111/ecog.02881

Rohr, T., Richardson, A. J., Lenton, A., Chamberlain, M. A., & Shadwick, E. H. (2023). Zooplankton grazing is the largest source of uncertainty for marine carbon cycling in CMIP6 models. *Communications Earth & Environment*, *4*(1), 1–22. https://doi.org/10.1038/s43247-023-00871-w

Sarofim, M. C., Smith, C. J., Malek, P., McDuffie, E. E., Hartin, C. A., Lay, C. R., & McGrath, S. (2024). High radiative forcing climate scenario relevance analyzed with a ten-million-member ensemble. *Nature Communications*, *15*(1), 8185. https://doi.org/10.1038/s41467-024-52437-9

Schmidt, K., Birchill, A. J., Atkinson, A., Brewin, R. J. W., Clark, J. R., Hickman, A. E., Johns, D. G., Lohan, M. C., Milne, A., Pardo, S., Polimene, L., Smyth, T. J., Tarran, G. A., Widdicombe, C. E., Woodward, E. M. S., & Ussher, S. J. (2020). Increasing picocyanobacteria success in shelf waters contributes to long-term food web degradation. *Global Change Biology*, *26*(10), 5574–5587. https://doi.org/10.1111/gcb.15161

Searle, K. R., Regan, C. E., Perrow, M. R., Butler, A., Rindorf, A., Harris, M. P., Newell, M. A., Wanless, S., & Daunt, F. (2023). Effects of a fishery closure and prey abundance on seabird diet and breeding success: Implications for strategic fisheries management and seabird conservation. *Biological Conservation*, *281*(March), 109990–109990. https://doi.org/10.1016/j.biocon.2023.109990

Shepherd, T. G., Boyd, E., Calel, R. A., Chapman, S. C., Dessai, S., Dima-West, I. M., Fowler, H. J., James, R., Maraun, D., Martius, O., Senior, C. A., Sobel, A. H., Stainforth, D. A., Tett, S. F. B., Trenberth, K. E., van den Hurk, B. J. J. M., Watkins, N. W., Wilby, R. L., & Zenghelis, D. A. (2018). Storylines: An alternative approach to representing uncertainty in physical aspects of climate change. *Climatic Change*, *151*(3), 555–571. https://doi.org/10.1007/s10584-018-2317-9

Simpson, J. H., & Sharples, J. (2012). *Introduction to the physical and biological oceanography of shelf seas* (p. 424). Cambridge University Press.

Skagseth, Ø., Broms, C. T., Gundersen, K., Hátún, H., Kristiansen, I., Larsen, K. M. H., Mork, K. A., Petursdottir, H., & Søiland, H. (2022). Arctic and Atlantic Waters in the Norwegian Basin, between year variability and potential ecosystem implications. *Frontiers in Marine Science*, *9*, 831739–831739. https://doi.org/10.3389/fmars.2022.831739

Soley-Guardia, M., Alvarado-Serrano, D. F., & Anderson, R. P. (2024). Top ten hazards to avoid when modeling species distributions: A didactic guide of assumptions, problems, and recommendations. *Ecography*, 1–21. https://doi.org/10.1111/ecog.06852

Song, J. (2015). Bias corrections for Random Forest in regression using residual rotation. *Journal of the Korean Statistical Society*, *44*(2), 321–326. https://doi.org/10.1016/j.jkss.2015.01.003

Taylor, K. E., Stouffer, R. J., & Meehl, G. A. (2012). An Overview of CMIP5 and the Experiment Design. *Bulletin of the American Meteorological Society*, *93*(4), 485–498. https://doi.org/10.1175/BAMS-D-11-00094.1

Teutschbein, C., & Seibert, J. (2012). Bias correction of regional climate model simulations for hydrological climate-change impact studies: Review and evaluation of different methods. *Journal of Hydrology*, *456–457*, 12–29. https://doi.org/10.1016/j.jhydrol.2012.05.052

The Mathworks Inc. (2021). *MATLAB version 9.11.0.1769968 (R2023b), Natick, Massachusetts: The MathWorks Inc. Https://www.mathworks.com* [Computer software].

Thyng, K. M., Greene, C. A., Hetland, R. D., Zimmerle, H. M., & DiMarco, S. F. (2016). True Colors of Oceanography: Guidelines for Effective and Accurate Colormap Selection. *Oceanography*, *29*(3), 9–13. https://doi.org/10.5670/oceanog.2016.66

Tinker, J., Lowe, J., Holt, J., Pardaens, A., & Wiltshire, A. (2015). Validation of an ensemble modelling system for climate projections for the northwest European shelf seas. *Progress in Oceanography*, *138*(November), 211–237. https://doi.org/10.1016/j.pocean.2015.07.002

Tinker, J., Lowe, J., Pardaens, A., Holt, J., & Barciela, R. (2016). Uncertainty in climate projections for the 21st century northwest European shelf seas. *Progress in Oceanography*, *148*, 56–73. https://doi.org/10.1016/j.pocean.2016.09.003

Tinker, J., Palmer, M. D., Harrison, B. J., O'Dea, E., Sexton, D. M. H., Yamazaki, K., & Rostron, J. W. (2024). Twenty-first century marine climate projections for the NW European shelf seas based on a perturbed parameter ensemble. *Ocean Science*, *20*(3), 835–885. https://doi.org/10.5194/os-20-835-2024

Tonani, M., & Ascione, I. (2021). *Product User Manual: Ocean Physical and Biogeochemical reanalysis: NWSHELF_MULTIYEAR_PHY_004_009; NWSHELF_MULTIYEAR_BGC_004_011* (Issue March, pp. 1–25). https://catalogue.marine.copernicus.eu/documents/PUM/CMEMS-NWS-PUM-004-009-011.pdf

Trites, A. W. (2021). Behavioral Insights into the Decline and Natural History of Steller Sea Lions. In C. Campagna & R. Harcourt (Eds.), *Ethology and Behavioral Ecology of Otariids and the Odobenid* (pp. 489–519). Springer International Publishing. https://doi.org/10.1007/978-3-030-59184-7_23

Tyldesley, E., Banas, N. S., Diack, G., Kennedy, R., Gillson, J., Johns, D. G., & Bull, C. (2024). Patterns of declining zooplankton energy in the northeast Atlantic as an indicator for marine survival of Atlantic salmon. *ICES Journal of Marine Science*, fsae077. https://doi.org/10.1093/icesjms/fsae077

Utne, K. R., Pauli, B. D., Haugland, M., Jacobsen, J. A., Maoileidigh, N., Melle, W., Broms, C. T., Nøttestad, L., Holm, M., Thomas, K., & Wennevik, V. (2021). *Poor feeding opportunities and reduced condition factor for salmon post-smolts in the Northeast Atlantic Ocean*.

Utne, K. R., Skagseth, Ø., Wennevik, V., Broms, C. T., Melle, W., & Thorstad, E. B. (2022). Impacts of a changing ecosystem on the feeding and feeding conditions for Atlantic salmon during the first months at sea. *Frontiers in Marine Science*, *9*, 1–13. https://doi.org/10.3389/fmars.2022.824614

Villarino, E., Chust, G., Licandro, P., Butenschön, M., Ibaibarriaga, L., Larrañaga, A., & Irigoien, X. (2015). Modelling the future biogeography of North Atlantic zooplankton communities in response to climate change. *Marine Ecology Progress Series*, *531*, 121–142. https://doi.org/10.3354/meps11299

Vollset, K. W., Urdal, K., Utne, K., Thorstad, E. B., Sægrov, H., Raunsgard, A., Skagseth, Ø., Lennox, R. J., Østborg, G. M., Ugedal, O., Jensen, A. J., Bolstad, G. H., & Fiske, P. (2022). *Ecological regime shift in the Northeast Atlantic Ocean revealed from the unprecedented reduction in marine growth of Atlantic salmon*. *2542*(March).

Wakelin, S. L., Artioli, Y., Holt, J. T., Butenschön, M., & Blackford, J. (2020a). Controls on near-bed oxygen concentration on the Northwest European Continental Shelf under a potential future climate scenario. *Progress in Oceanography*, *187*(October 2019), 102400–102400. https://doi.org/10.1016/j.pocean.2020.102400

Wakelin, S. L., Artioli, Y., Holt, J. T., Butenschön, M., & Blackford, J. (2020b). *Northwest Europe NEMO-ERSEM ocean model hindcast and climate projection under RCP8.5* (Version 1.0) [Dataset]. Zenodo. https://doi.org/10.5281/zenodo.3953801

Wanless, S., Harris, M. P., Newell, M. A., Speakman, J. R., & Daunt, F. (2018). Community-wide decline in the occurrence of lesser sandeels Ammodytes marinus in seabird chick diets at a North Sea colony. *Marine Ecology Progress Series*, *600*, 193–206. https://doi.org/10.3354/meps12679

Wanless, S., Harris, M. P., Redman, P., & Speakman, J. R. (2005). Low energy values of fish as a probable cause of a major seabird breeding failure in the North Sea. *Marine Ecology Progress Series*, *294*, 1–8. https://doi.org/10.3354/meps294001

Wilson, R. (2015). *A unified population model of Calanus finmarchicus and C. helgolandicus in the North Atlantic*.

Wilson, R., Heath, M. R., & Speirs, D. C. (2016). Spatial modeling of Calanus finmarchicus and Calanus helgolandicus: Parameter differences explain differences in biogeography. *Frontiers in Marine Science*, *3*(SEP). https://doi.org/10.3389/fmars.2016.00157

Wilson, Speirs, D. C., & Heath, M. R. (2015). On the surprising lack of differences between two congeneric calanoid copepod species, Calanus finmarchicus and C. helgolandicus. *Progress in Oceanography*, *134*, 413–431. https://doi.org/10.1016/j.pocean.2014.12.008

Winder, M., Bouquet, J.-M., Bermúdez, J. R., Berger, S. A., Hansen, T., Brandes, J., Sazhin, A. F., Nejstgaard, J. C., Båmstedt, U., Jakobsen, H. H., Dutz, J., Frischer, M. E., Troedsson, C., & Thompson, E. M. (2017). *Increased Appendicularian zooplankton alter carbon cycling under warmer more acidified ocean conditions*. *62*, 1541–1551. https://doi.org/10.1002/lno.10516

Yool, A., Popova, E. E., & Coward, A. C. (2015). Future change in ocean productivity: Is the Arctic the new Atlantic? *Journal of Geophysical Research: Oceans*, *120*(12), 7771–7790. https://doi.org/10.1002/2015JC011167

Zhang, G., & Lu, Y. (2012). Bias-corrected random forests in regression. *Journal of Applied Statistics*, *39*(1), 151–160. https://doi.org/10.1080/02664763.2011.578621

ACKNOWLEDGEMENTS

This work was funded by the Natural Environment Research Council (NERC) EcoWind Ecosystem Change, Offshore Wind, Net Gain, and Seabirds (ECOWINGS) grant NE/X008983/1 and the United Kingdom Missing Salmon Alliance under the Likely Suspects Framework project.

The regional climate projections were generated under the NERC projects Resolving Climate Impacts on shelf and CoastaL sea Ecosystems (ReCICLE) lead by the National Oceanography Centre (NE/M003477/1) and Regional Ecosystem & Biogeochemical Impacts of Ocean Acidification (ROAM) lead by Plymouth Marine Laboratory (NE/H017372/1).

The Continuous Plankton Recorder sampling was supported by NERC Atlantic Climate and Environment Strategic Science – AtlantiS.

We thank Dr Colleen Petrik for useful discussions and kindly yhusupplying the gridded global zooplankton projections from Petrick et al. (2022) to enable us to calculate the projected percent change for the Northeast Atlantic.

AUTHOR CONTRIBUTIONS

All authors contributed to Writing – review and editing. E.T.: Methodology, Formal analysis, Investigation, Software, and Writing – original draft; N.B.: Conceptualization, Methodology, Formal analysis, and Supervision; D.S. and M.H.: Conceptualization, Methodology and Supervision; C.B.: Funding acquisition, and Supervision; S.W. and J.J.: Data curation and Method; D.G.: Data curation; V.K.: Method; K.S. and F.D.: Conceptualization and Funding acquisition.

COMPETING INTERESTS

The authors declare no competing interests.

TABLES

Table 1: Summary of model simulations used to derive past and future ocean conditions.

Simulation	Time period	Horizontal grid	Vertical grid	Forcing data		Output temporal resolution	References
				Atmospheric	Ocean boundary		
Reanalysis	1993-2023	Atlantic Margin Model 7 km resolution, 40° to 65°N, –20° to 13° E (AMM7)	Interpolated to 24 fixed depth layers.	ECMWF ERA5	GloSea5	Monthly and daily	(E.U. Copernicus Marine Service Information (CMEMS), Marine Data Store (MDS), 2025b, 2025a; Tonani & Ascione, 2021)
ROAM-HADGEM2	1980-2099		33 terrain-following depth layers	HadGEM2-ES	NEMO-MEDUSA	Physics daily, biogeochemistry monthly	(Holt et al., 2018; Jones et al., 2011; Wakelin et al., 2020a, 2020b; Yool et al., 2015)
RECICLE-IPSL			51 terrain-following depth layers	IPSL-CM5A-MR		Monthly and daily	(Galli et al., 2023b)
RECICLE-GFDL				GFDL-ESM2G		Monthly and daily	(Galli et al., 2023a)

Table 2: Projected percentage change from recent past by zooplankton class and climate projection model. Mean is climate projection ensemble mean.

Zooplankton class	Region	Near future (2040-2050)				Far future (2090-2100)			
		HADGEM	IPSL	GFDL	Mean	HADGEM	IPSL	GFDL	Mean
Small copepods	Off-shelf	-84	-87	-50	-74	-90	-90	-87	-89
	Shelf	-60	-72	-23	-52	-88	-81	-76	-82
	Northeast Atlantic	-67	-76	-31	-58	-89	-84	-79	-84
C. finmarchicus	Off-shelf	-63	-90	-66	-73	-92	-95	-95	-94
	Shelf	-70	-75	-44	-63	-96	-83	-84	-87
	Northeast Atlantic	-64	-88	-63	-72	-93	-93	-94	-93
C. helgolandicus	Off-shelf	-70	-68	-16	-51	-69	-47	-50	-56
	Shelf	-80	-90	-23	-64	-92	-90	-79	-87
	Northeast Atlantic	-79	-88	-23	-63	-89	-86	-77	-84

FIGURES

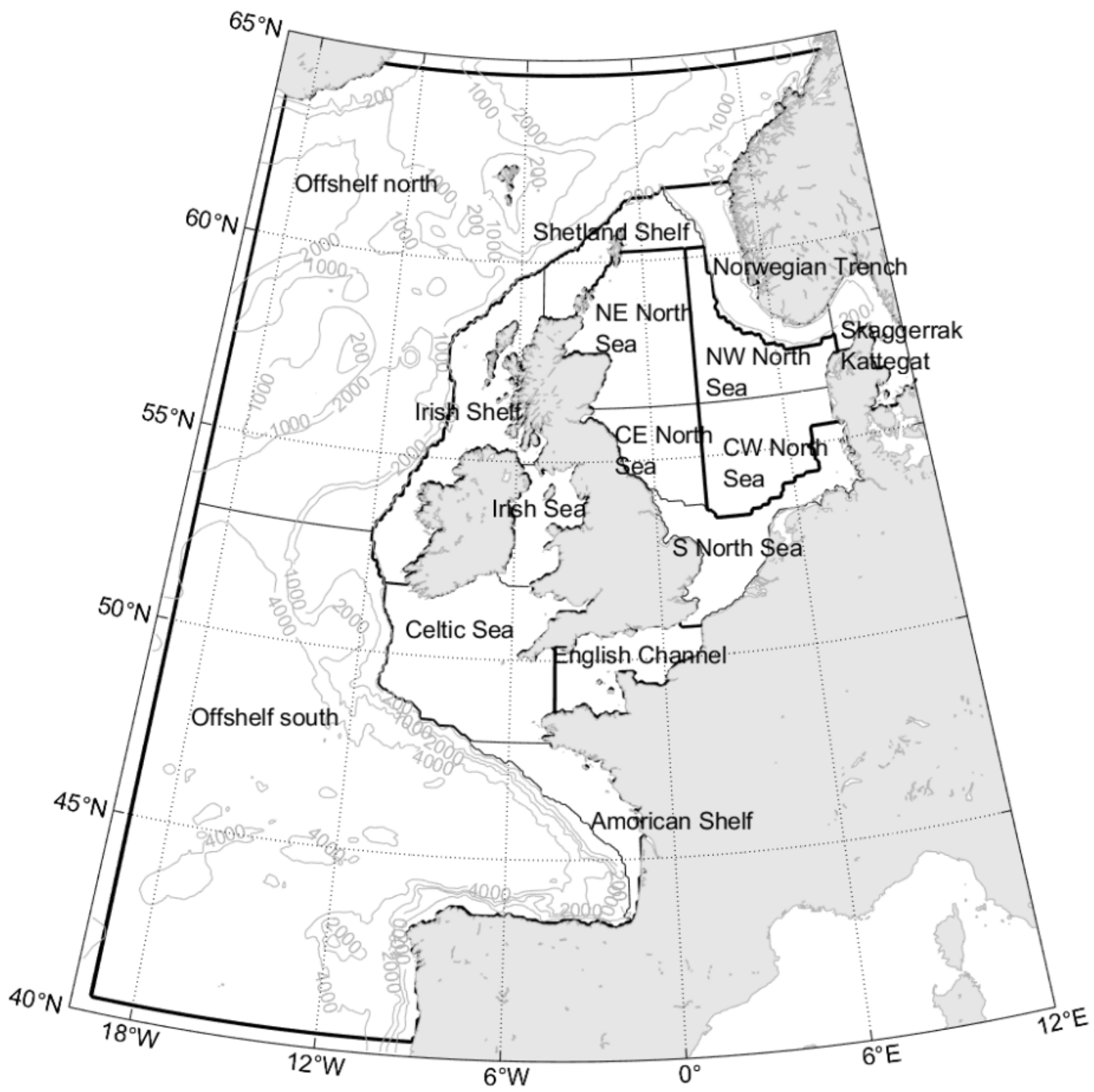

Figure 1: Model domain and averaging regions.

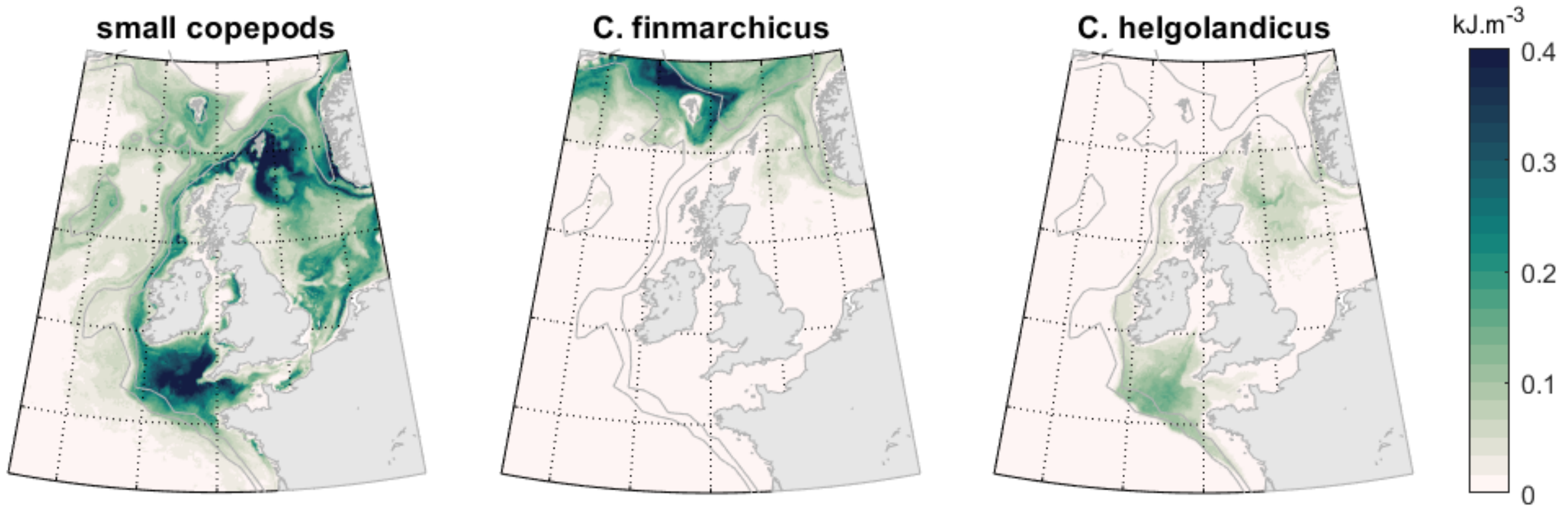

Figure 2: Modelled recent past distributions of aggregated small copepods, *Calanus finmarchicus* and *Calanus helgolandicus*: mean over 2010-2022. Contour lines at 200 m and 800 m to indicate shelf edge.

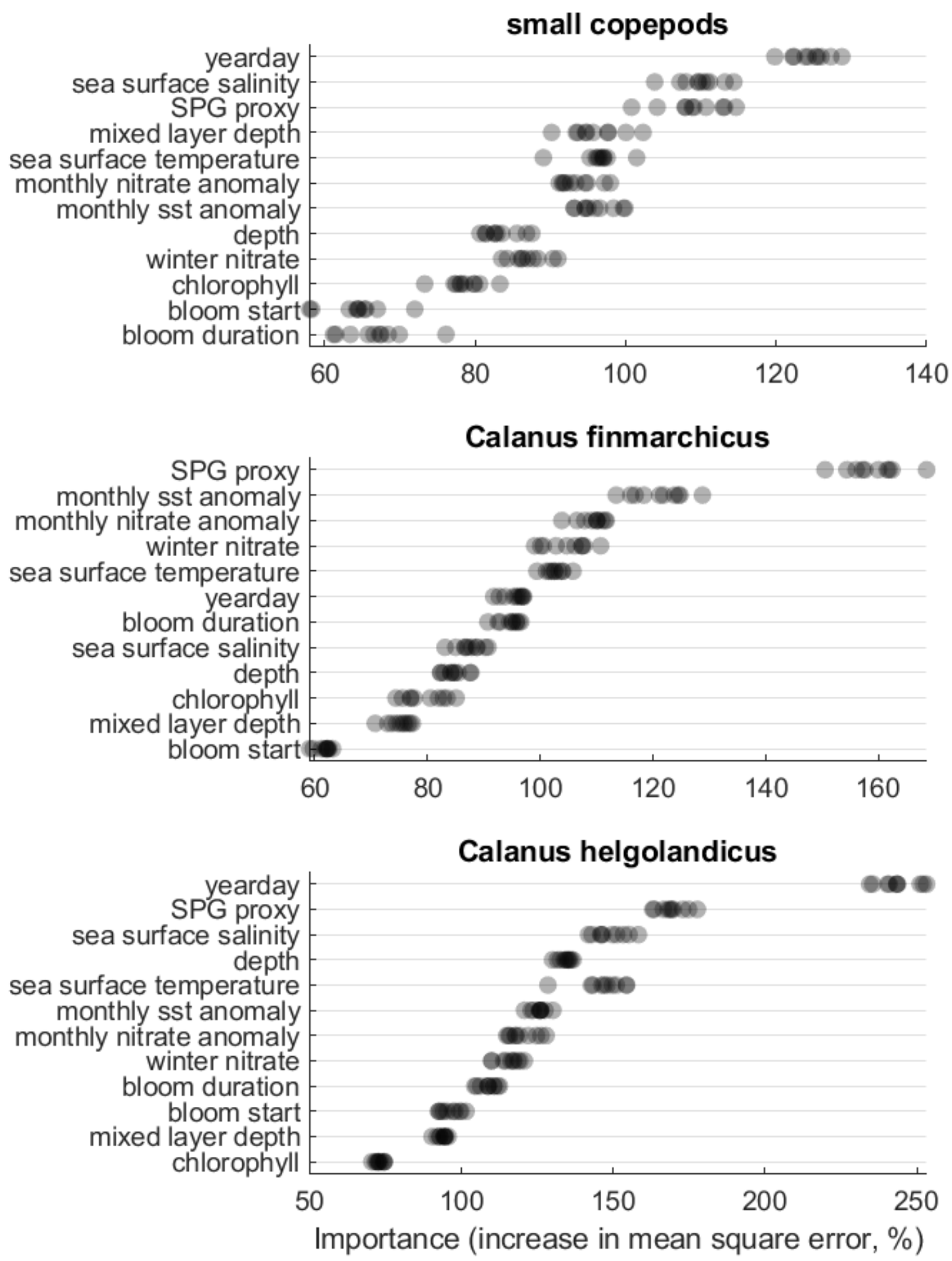

Figure 3: Importance ranking of predictors for each zooplankton class. Each point represents a repetition of training the models with the same parameters but with random subsampling of data for each tree and predictors at each tree node.

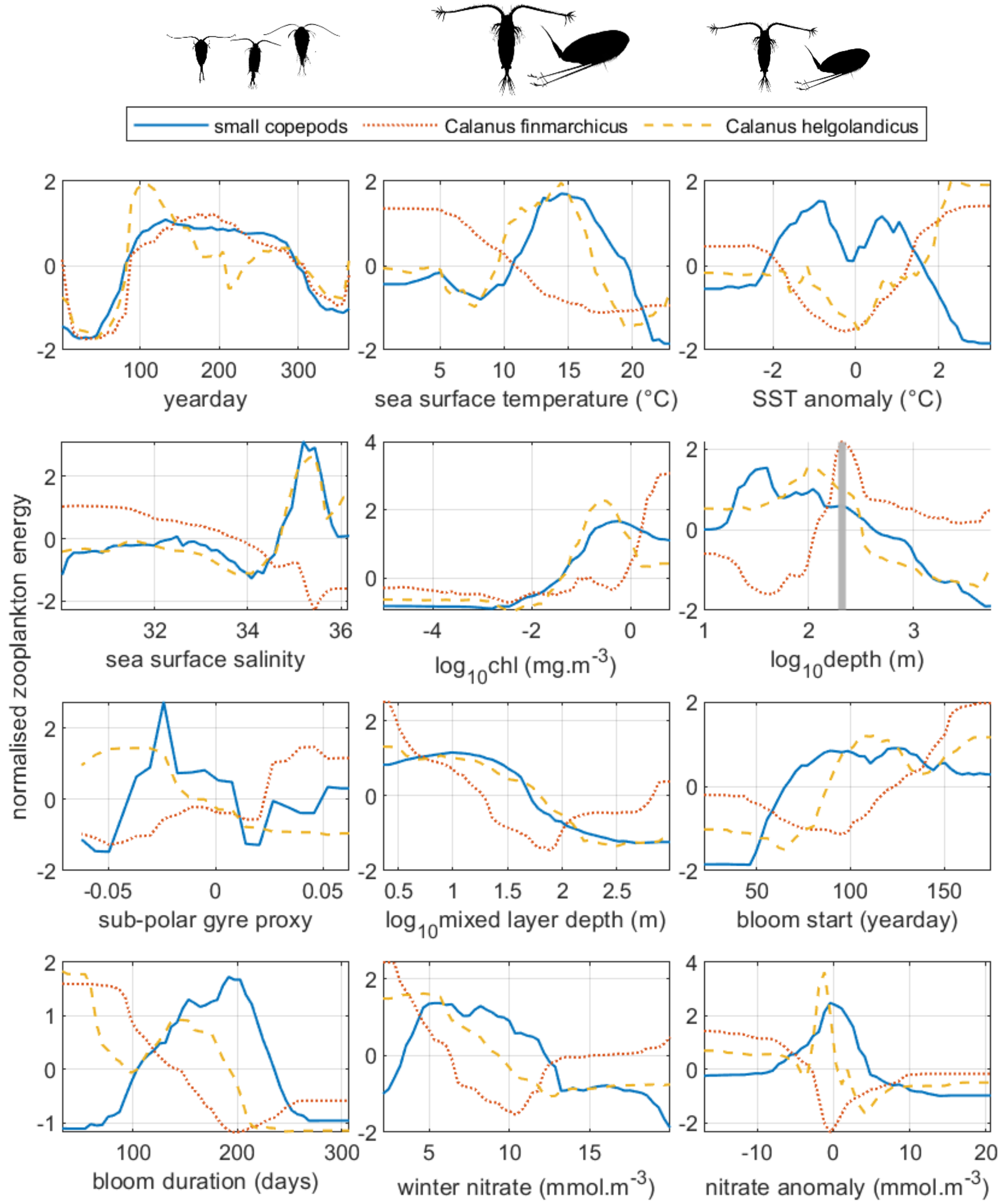

Figure 4: Partial dependence of normalised zooplankton energy on each predictor. Vertical line in depth plot indicates the continental shelf edge.

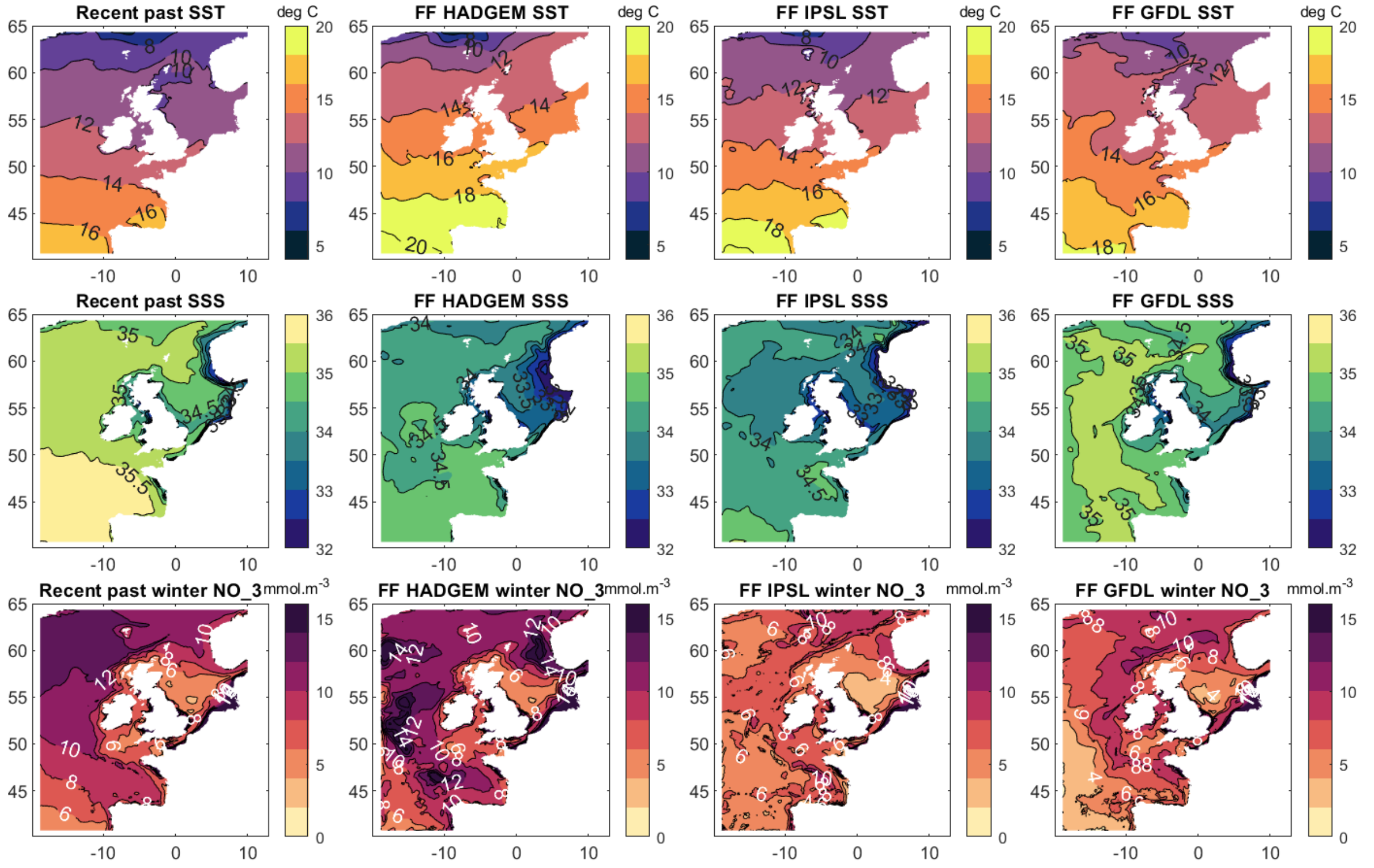

Figure 5: Bias corrected far future mean decadal sea surface temperature, salinity and winter nitrate concentration for each climate projection.

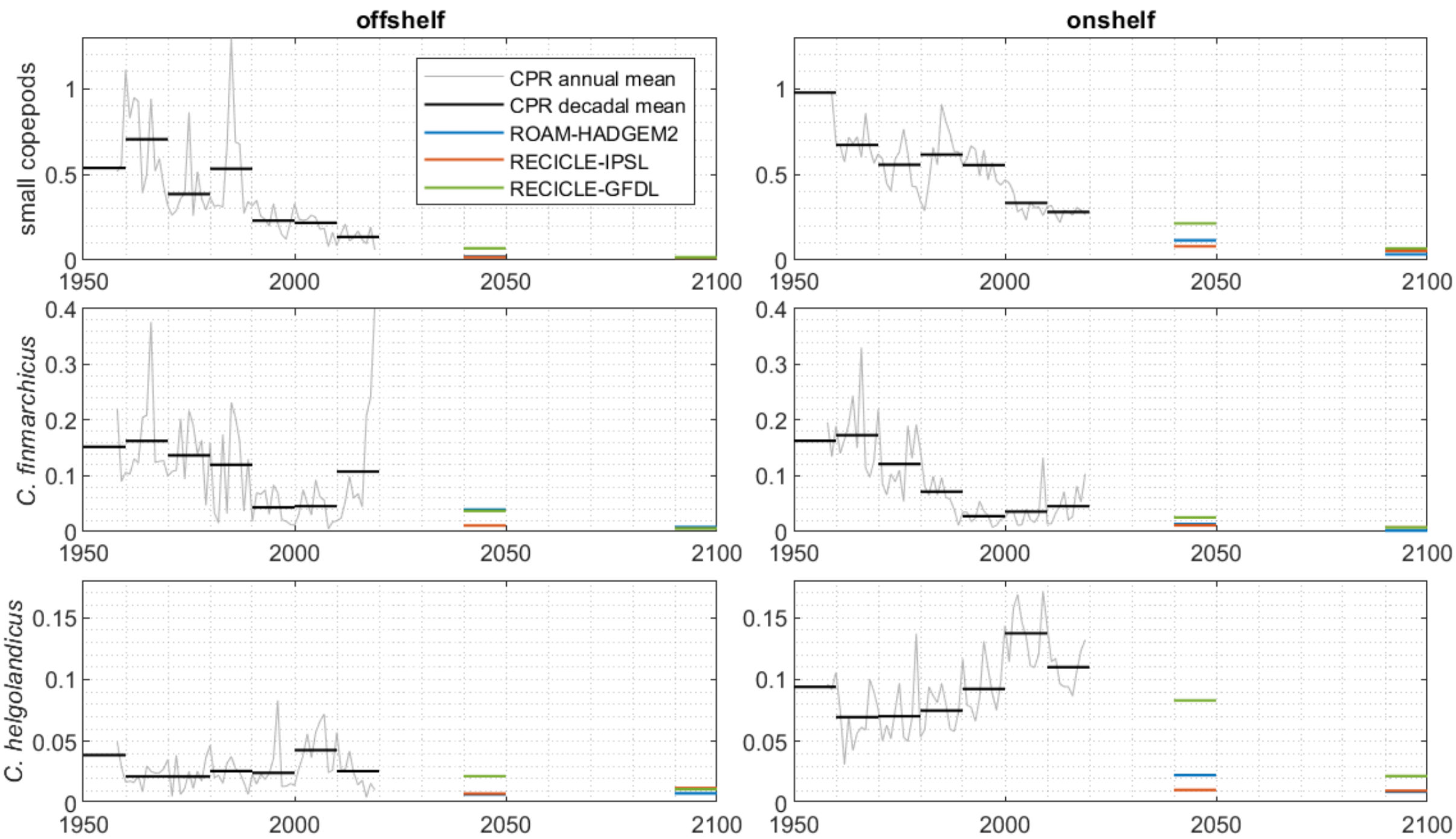

Figure 6: Observed mean annual and decadal Continuous Plankton Recorder zooplankton energy and modelled projected zooplankton energy for shelf (<200 m water depth) and off-shelf regions of the Northeast Atlantic.

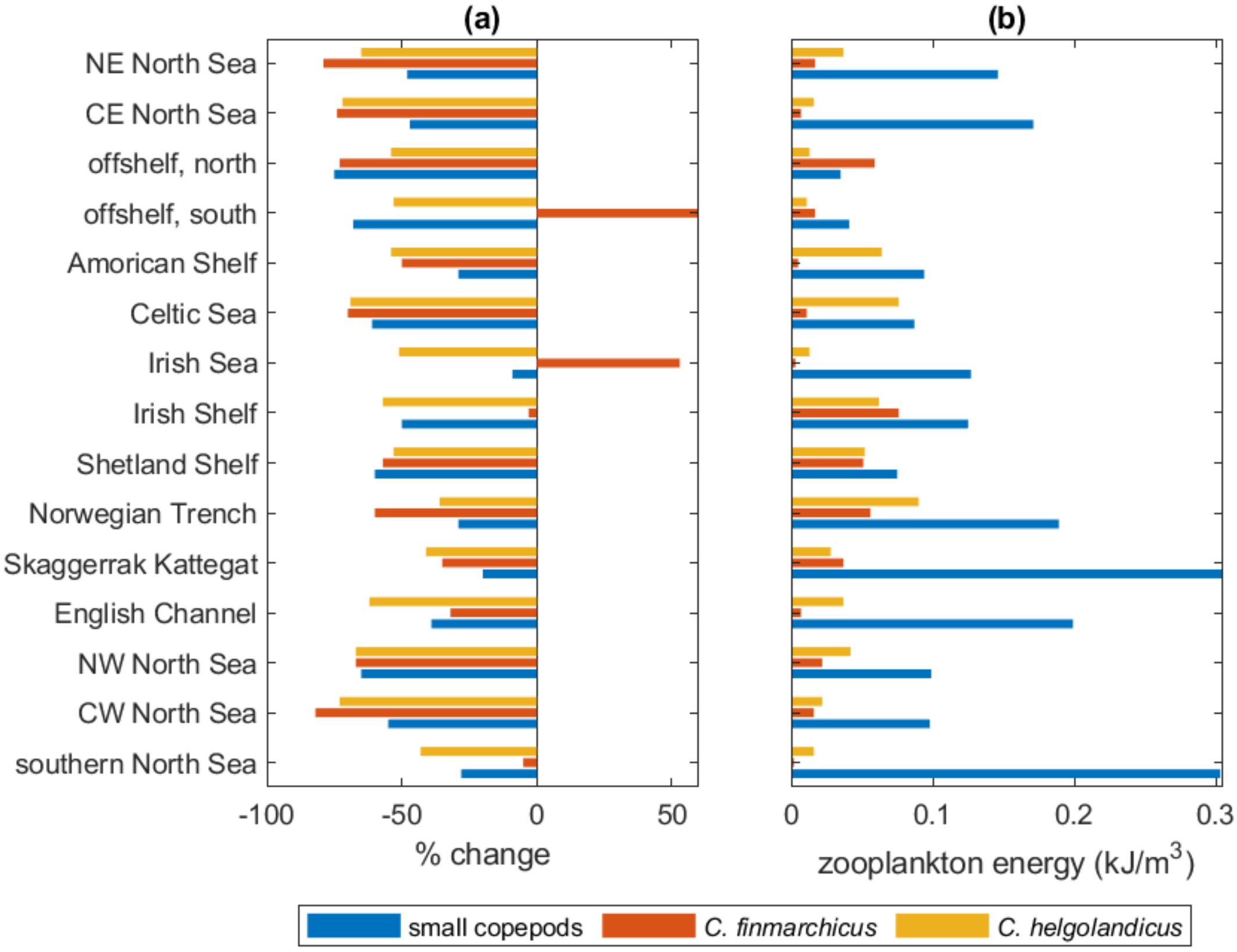

Figure 7: Projected near future (NF) ensemble mean (a) percentage change and (b) absolute zooplankton energy by taxa and Northeast Atlantic region. Note that the *C. finmarchicus* increase in offshelf south (800%) extends beyond the upper limit of the percentage change axis, which is curtailed to allow interpretation for the other regions. Historical *C. finmarchicus* abundance in this region is negligible, so the resulting projected densities are still low in comparison with historical densities in the core regions for this species (see b).

SUPPLEMENTARY INFORMATION

Sub-polar gyre proxy

The sub-polar gyre strength proxy is based on monthly upper ocean (50-500 m) salinity calculated from the AMM7 physical reanalysis. We calculated the correlation coefficient between the upper ocean salinity at each grid-cell and a monthly historical SPG index for 1993-2018 (Chafik, 2019; Hátún & Chafik, 2018). The strongest correlation was *R*=-0.7 at 12.1W 54.1N near the shelf edge west of Ireland (Figure 1). The time series of inverted salinity anomaly at this location was then used as the SPG proxy in this study.

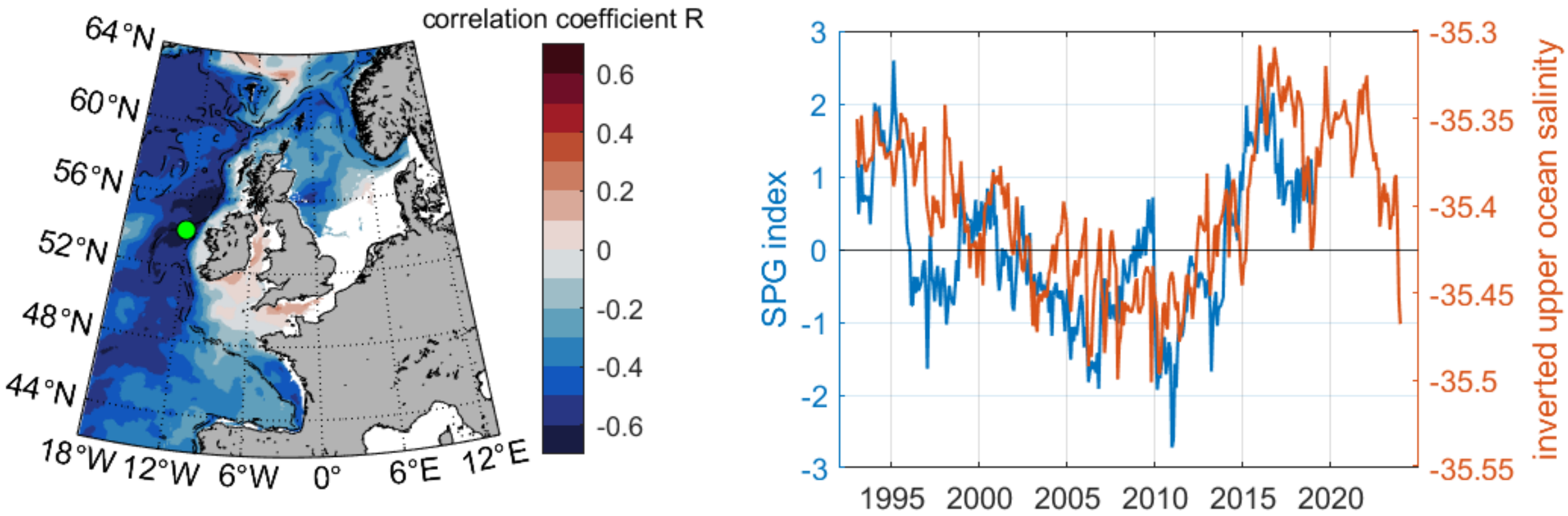

Figure 1: Correlation between upper ocean salinity and sub-polar gyre index over 1993-2018 and time series of both at the grid-cell with strongest correlation (indicated by green dot).

Block cross-validation

With irregular sampling such as the CPR, there is no obvious or agreed spatial blocking strategy, e.g. equal area or equal sample frequency. The three zooplankton groups also vary in their spatial distributions so that no blocking strategy would be ideal for all three. We therefore divided the space into 9 regular contiguous blocks (longitude bounds at -20, -10, 0 and 10°E, latitude bounds at 40, 48, 56 and 65°N) to avoid introducing bias through a selective design. The RMSE error on the test data was on average approximately 2.5 times higher than on the training run (Table). The mean R^2 on the test data was 0.27 for small copepods, 0.22 for Calanus finmarchicus and 0.17 for Calanus helgolandicus. The models performed below average in two cases: (1) test block was outside the main species distribution, e.g. attempting to predict *C. finmarchicus* abundance in the Celtic Sea (block 5), and (2) excluded test block had high species abundance resulting in a loss of key training data, e.g. small copepods in the southern North Sea (block 7). The first case implies that the models will have lower predictive abilities at the edge of species ranges, as found previously (Brun et al., 2016), some areas of which may become areas of range expansion under climate change. The second case will result in an underestimate of the full models' predictive abilities.

For temporal cross-validation the 22-year (1998-2019) full dataset was split into three contiguous blocks of 8 (1998-2005), 7 (2006-2012) and 7 (2013-2019) years to allow for reasonably long blocks without overlap (Table 2). The predictive performance on the test data was similar for each left-out period, with a mean R^2 of 0.33, 0.25 and 0.24 for small copepods, *C. finmarchicus* and *C. helgolandicus*, respectively. The importance ranking was relatively stable for small copepods and *C. helgolandicus* but varied for *C. finmarchicus* (Figure 2). This is because the SPG proxy, the strongest predictor for *C. finmarchicus*, varies over longer periods than the size of the cross-validation blocks. Removal of this strong signal results in reordering of the remaining high-ranking

variables. Overall, the temporal cross-validation suggests reasonable predictive ability into time periods beyond the training data.

Table 1: Spatial cross-validation. Root-mean square error (RMSE; log_{10} kJ.m^{-3}) and Pearson's correlation (R^2) for model trained excluding one block at a time and testing on excluded block. Bold indicates blocks with low R^2 or high RMSE compared with the mean over all runs. Blank rows indicate regions with no test samples in a block.

		Small copepods				*C. finmarchicus*				*C. helgolandicus*			
		Training		Test		Training		Test		Training		Test	
**Block**	**n**	**RMSE**	**R^2**	**RMSE**	**R^2**	**RMSE**	**R^2**	**RMSE**	**R^2**	**RMSE**	**R^2**	**RMSE**	**R^2**
1	2038	1.07	0.92	2.67	0.26					0.68	0.93	1.43	0.22
2	4432	1.07	0.92	2.75	0.29					0.68	0.93	1.57	0.27
3	3467	1.08	0.92	2.47	0.45	0.56	0.93	1.85	0.40	0.69	0.93	1.37	**0.08**
4	5416	1.06	0.92	**2.82**	**0.18**					0.68	0.93	1.72	0.21
5	13103	1.07	0.92	2.60	0.28	0.58	0.93	1.60	**0.04**	0.66	0.93	1.95	**0.12**
6	8436	1.07	0.92	2.54	0.39	0.52	0.93	2.00	0.19	0.67	0.93	1.81	0.26
7	10627	1.07	0.92	**2.72**	**0.08**					0.65	0.93	1.95	**0.04**
8	5504	1.08	0.92	2.46	**0.23**	0.54	0.93	2.02	0.26	0.67	0.93	1.94	0.15
**Mean**		**1.07**	**0.92**	**2.63**	**0.27**	**0.55**	**0.93**	**1.87**	**0.22**	**0.67**	**0.93**	**1.72**	**0.17**

Table 2: Temporal cross-validation. Root-mean square error (RMSE; log_{10} kJ.m^{-3}) and Pearson's correlation (R^2) for model trained excluding one period at a time.

	Small				*C. finmarchicus*				*C. helgolandicus*			
	Training		Test		Training		Test		Training		Test	
**Block**	**RMSE**	**R^2**	**RMSE**	**R^2**	**RMSE**	**R^2**	**RMSE**	**R^2**	**RMSE**	**R^2**	**RMSE**	**R^2**
1998-2005	1.04	0.92	2.63	0.30	0.58	0.93	1.42	0.25	0.67	0.93	1.79	0.25
2006-2012	1.08	0.92	2.51	0.35	0.59	0.93	1.39	0.25	0.67	0.93	1.79	0.25
2013-2019	1.08	0.92	2.53	0.33	0.53	0.93	1.80	0.24	0.67	0.93	1.80	0.22
**Mean**	**1.07**	**0.92**	**2.56**	**0.33**	**0.56**	**0.93**	**1.53**	**0.25**	**0.67**	**0.93**	**1.79**	**0.24**

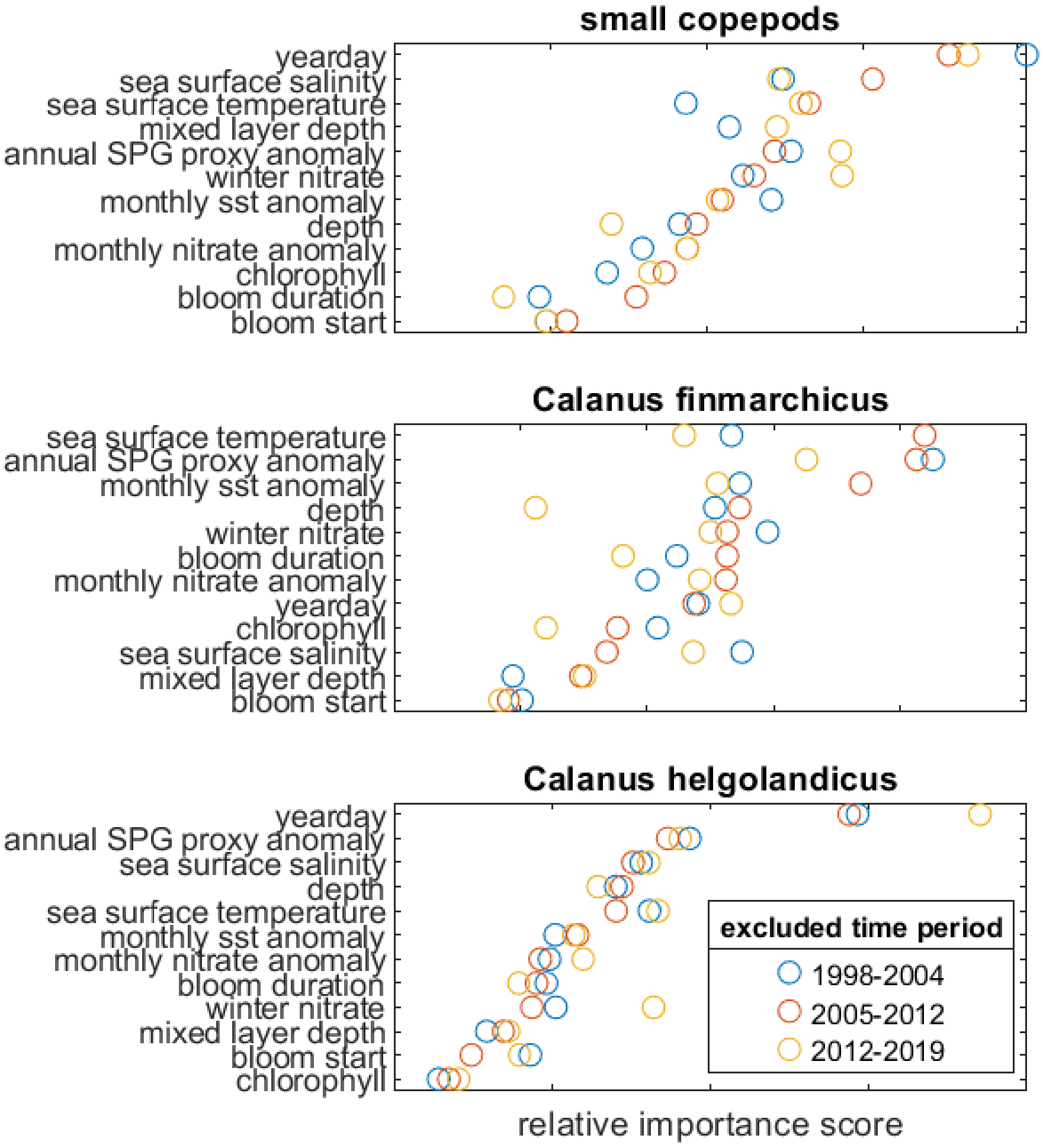

1076

1077 **Figure 2: Importance ranking by cross-validation time block.**

1078 **Bias corrected projected SST and SSS**

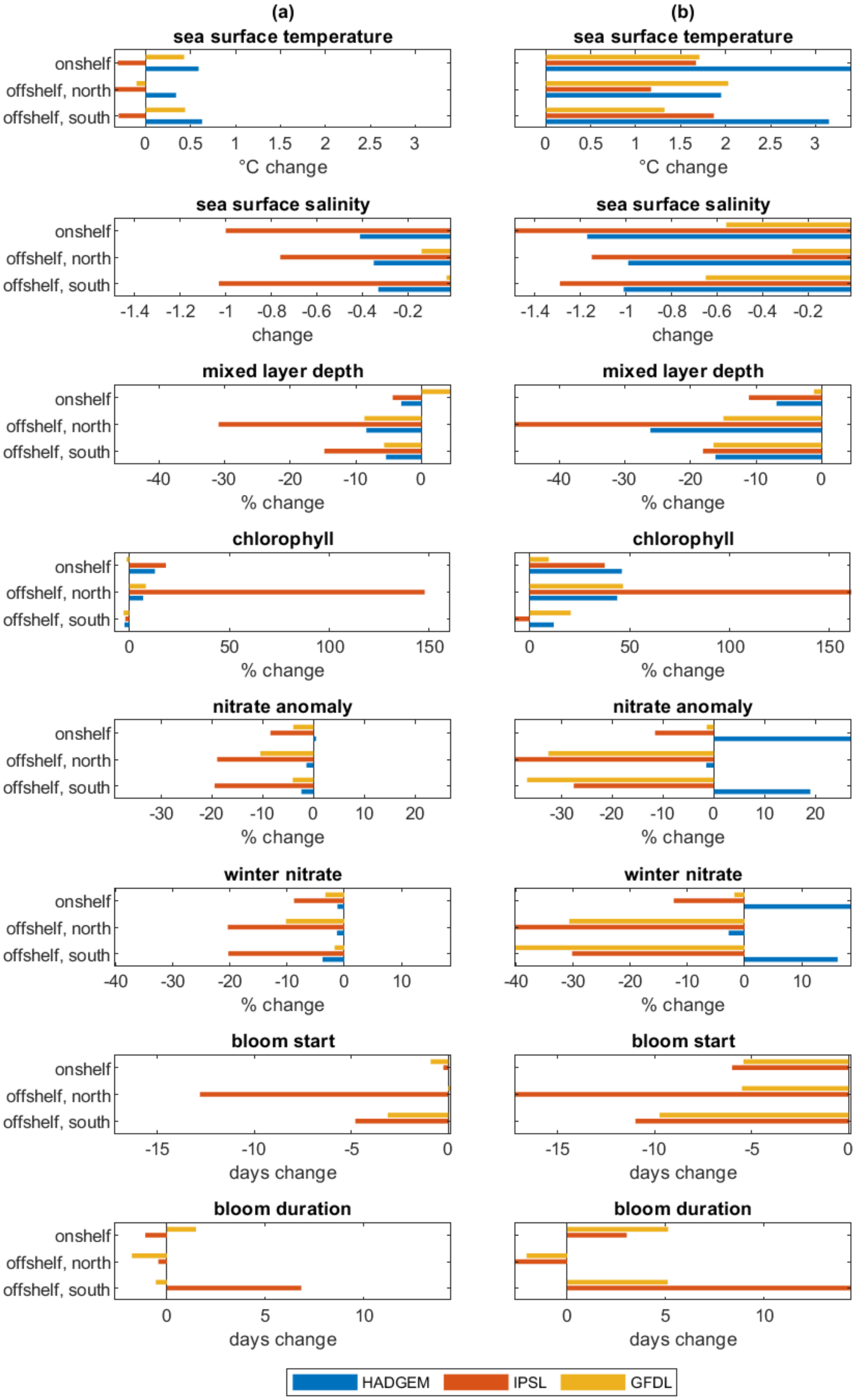

1079

1080 **Figure 3: Projected annual mean Delta change from recent past (2010s and 2020s) to (a) near future and (b) far**
1081 **future by predictor variable, climate projection model and region.**

Regional zooplankton projections

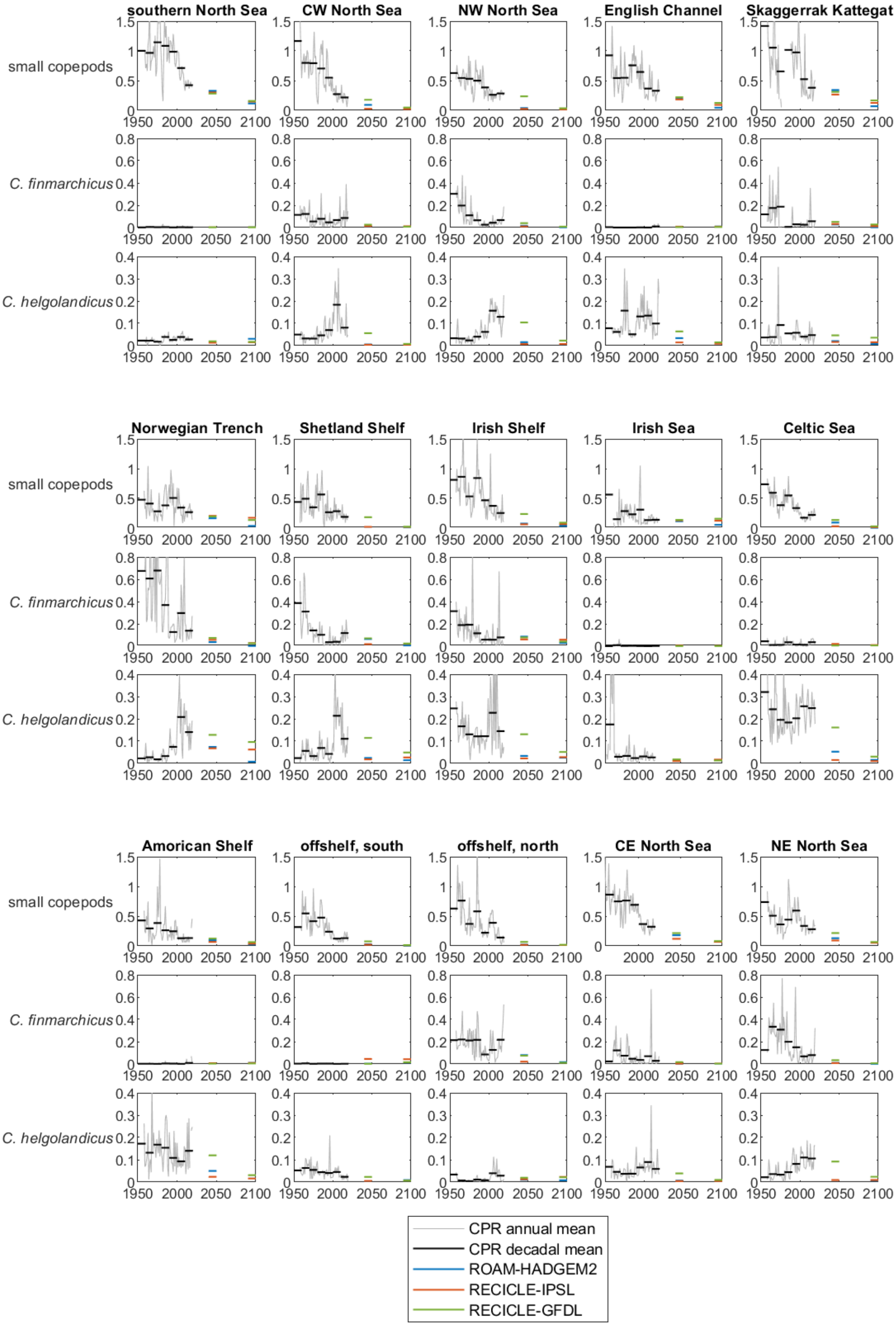

Figure 4: Observed mean annual and decadal Continuous Plankton Recorder zooplankton energy and modelled projected zooplankton energy by Northeast Atlantic region.

Zooplankton seasonality projections

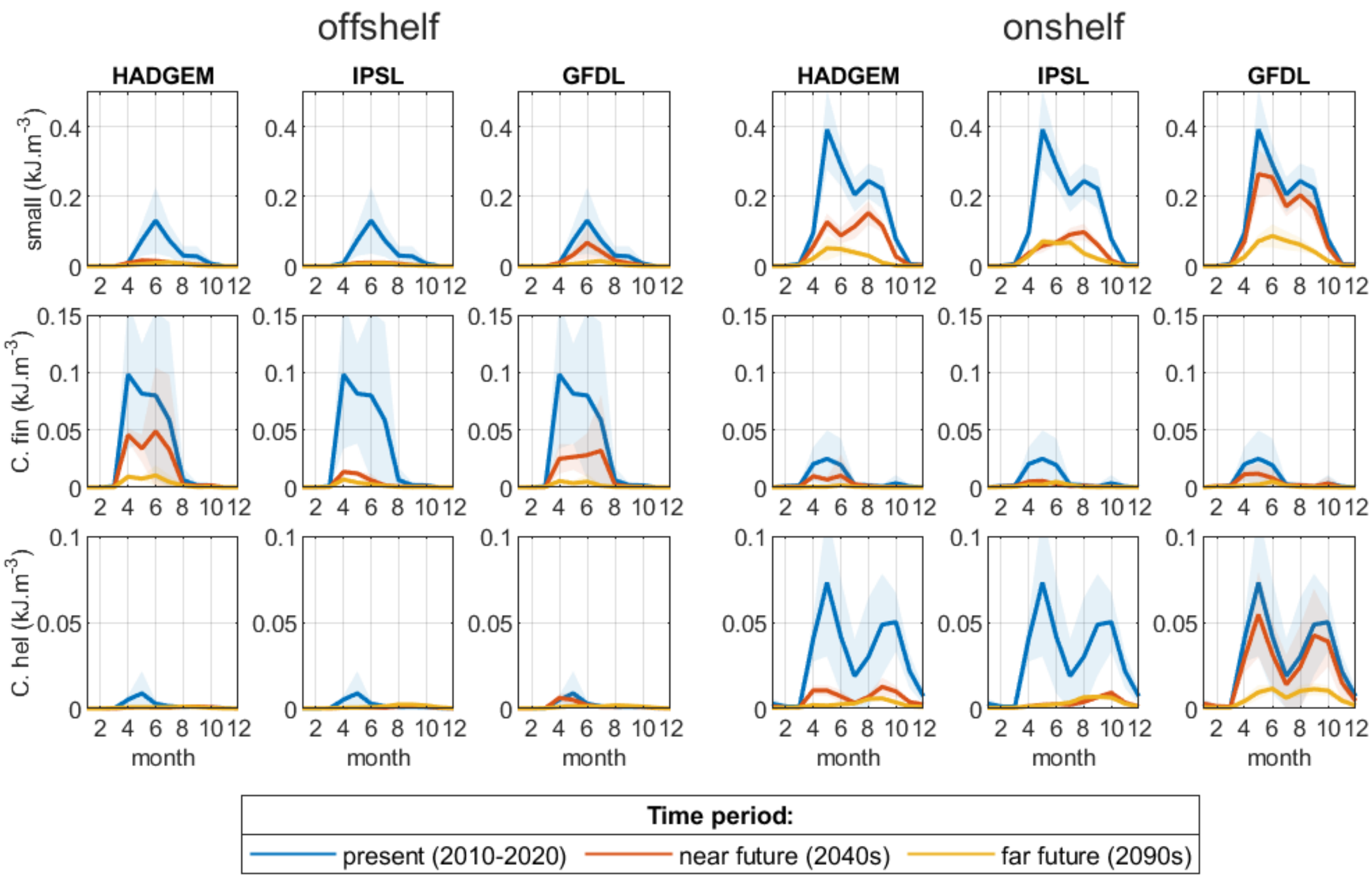

Figure 5: Mean modelled seasonal cycles by zooplankton class, time period and climate projection model for shelf (water depth < 200 m) and off-shelf areas of the Northeast Atlantic.

Mapping novel predictor space

Figure 6 shows the areas in the near and far future where environmental predictor variables are outside the bounds of the training data set. This was calculated globally, i.e. by checking at each grid-cell whether the projected variable was within the range of the training data over the whole model domain.

By the NF, the main areas with variables projected to exceed previous bounds are the Bay of Biscay (SST), Norwegian Trench (salinity and SST anomaly), inshore areas and eastern North Sea (SST anomaly), and central and northern North Sea (chlorophyll and nitrate anomaly). GFDL additionally shows areas of increased stratification off the continental shelf. By the FF, most of the domain is outside the training data because of the large increase in SST anomaly compared with the 1998-2023 climatology.

1102

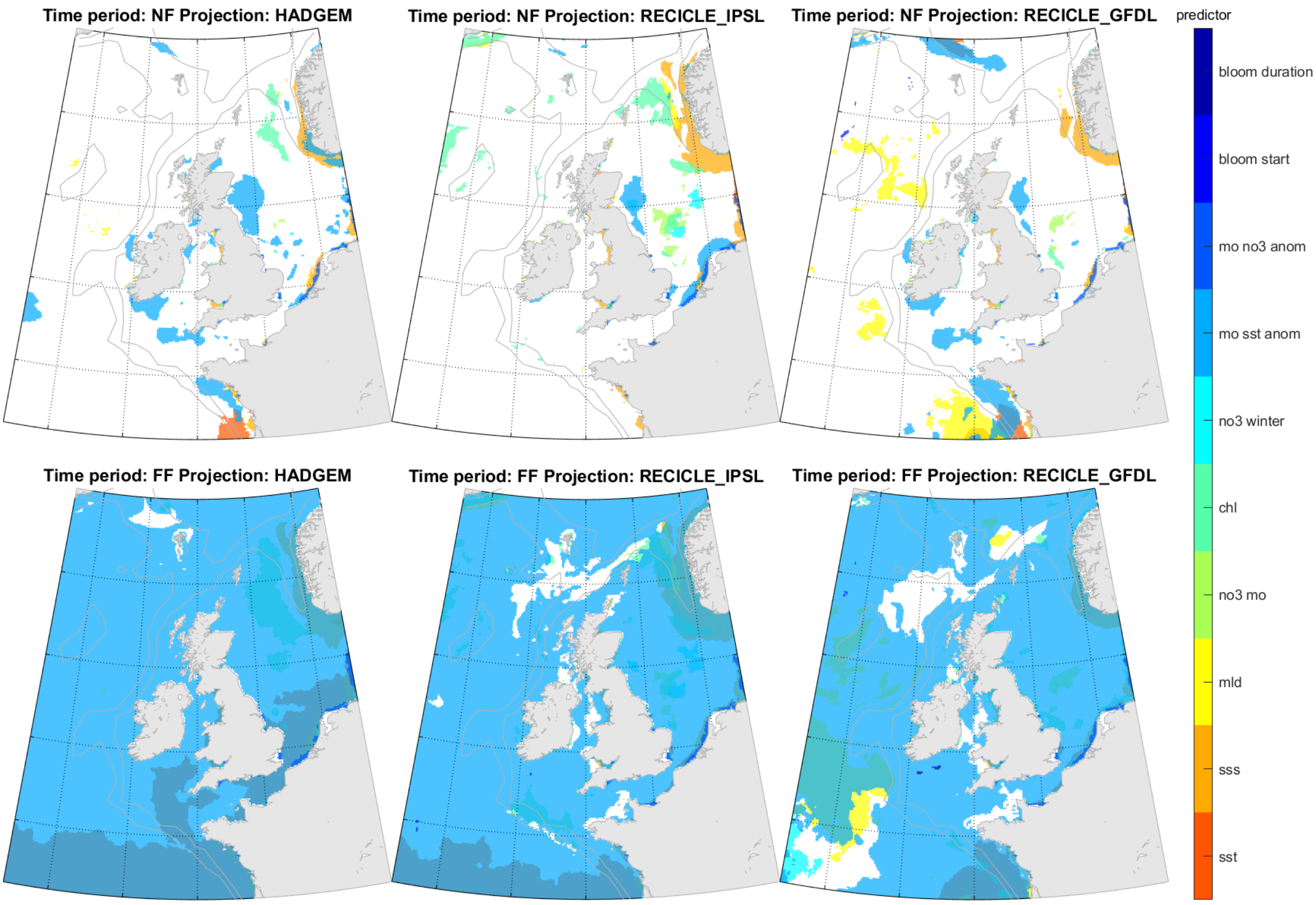

1103 **Figure 6: Areas in near future (NF) and far future (FF) with predictor variables outside the bounds of training data.**
