# Supplementary material for: Projected declines in zooplankton energy supporting Northwest European Shelf ecosystems
Source: Commun Earth Environ. 2026 Jul 23;7(1):897. doi: 10.1038/s43247-026-03840-1 (PMC13759333; doi:10.1038/s43247-026-03840-1)
Supplement: Supplementary file 2 [file 43247_2026_3840_MOESM2_ESM.pdf]

SUPPLEMENTARY INFORMATION

Sub-polar gyre proxy

The sub-polar gyre strength proxy is based on monthly upper ocean (50-500 m) salinity calculated from the AMM7 physical reanalysis. We calculated the correlation coefficient between the upper ocean salinity at each grid-cell and a monthly historical SPG index for 1993-2018 (Chafik, 2019; Hátún & Chafik, 2018). The strongest correlation was *R*=-0.7 at 12.1W 54.1N near the shelf edge west of Ireland (Figure S1). The time series of inverted salinity anomaly at this location was then used as the SPG proxy in this study.

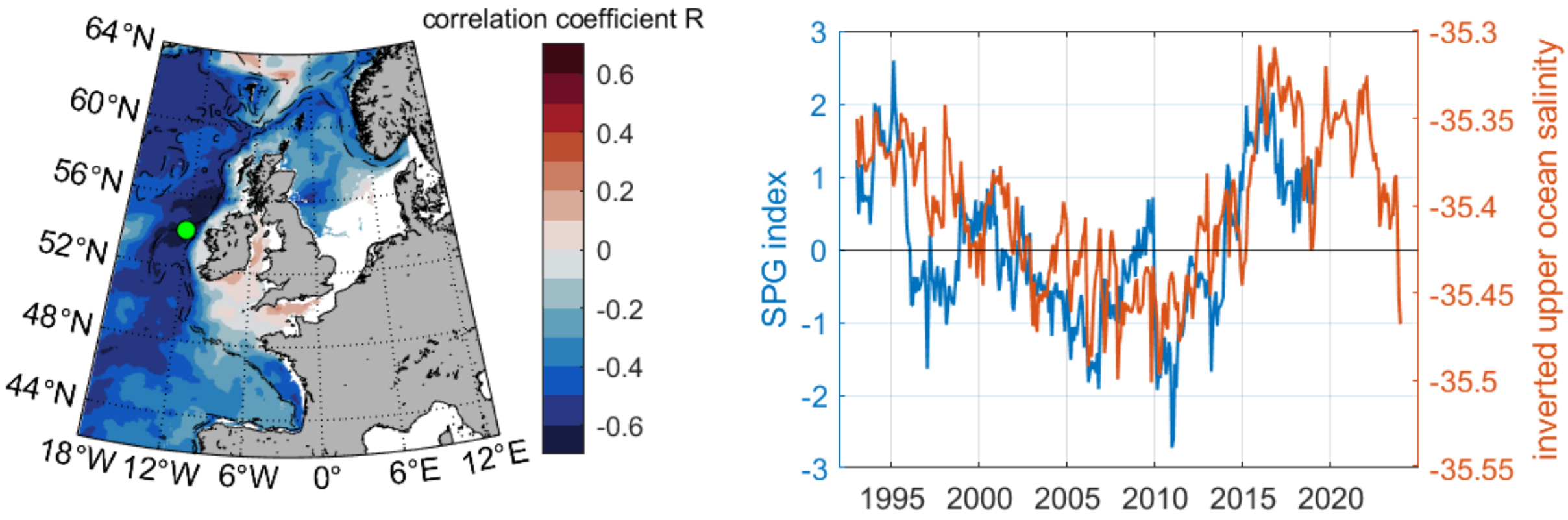

Figure S 1: Correlation between upper ocean salinity and the sub-polar gyre index over 1993-2018 and time series of both at the grid-cell with the strongest correlation (indicated by green dot).

Block cross-validation

With irregular sampling such as the CPR, there is no obvious or agreed spatial blocking strategy, e.g. equal area or equal sample frequency. The three zooplankton groups also vary in their spatial distributions so that no blocking strategy would be ideal for all three. We therefore divided the space into 9 regular contiguous blocks (longitude bounds at -20, -10, 0 and 10°E, latitude bounds at 40, 48, 56 and 65°N) to avoid introducing bias through a selective design. The RMSE error on the test data was on average approximately 2.5 times higher than on the training run (Table S1). The mean R^2 on the test data was 0.27 for small copepods, 0.22 for *Calanus finmarchicus* and 0.17 for *Calanus helgolandicus*. The models performed below average in two cases: (1) the test block was outside the main species distribution, e.g. attempting to predict *C. finmarchicus* abundance in the Celtic Sea (block 5), and (2) the excluded test block had high species abundance resulting in a loss of key training data, e.g. small copepods in the southern North Sea (block 7). The first case implies that the models will have lower predictive abilities at the edge of species ranges, as found previously (Brun et al., 2016), some areas of which may become areas of range expansion under climate change. The second case will result in an underestimate of the full models' predictive abilities.

For temporal cross-validation the 22-year (1998-2019) full dataset was split into three contiguous blocks of 8 (1998-2005), 7 (2006-2012) and 7 (2013-2019) years to allow for reasonably long blocks without overlap (Table S2). The predictive performance on the test data was similar for each left-out period, with a mean R^2 of 0.33, 0.25 and 0.24 for small copepods, *C. finmarchicus* and *C. helgolandicus*, respectively. The importance ranking was relatively stable for small copepods and *C. helgolandicus* but varied for *C. finmarchicus* (Table S2). This is because the SPG proxy, the strongest predictor for *C. finmarchicus*, varies over longer periods than the size of the cross-validation blocks. Removal of this strong signal results in reordering of the remaining

high-ranking variables. Overall, the temporal cross-validation suggests reasonable predictive ability into time periods beyond the training data.

Table S 1:Spatial cross-validation. Root-mean square error (RMSE; log_{10} kJ.m^{-3}) and Pearson's correlation (R^2) for model trained excluding one block at a time and testing on excluded block. Bold indicates blocks with low R^2 or high RMSE compared with the mean over all runs. Blank rows indicate regions with no test samples in a block.

		Small copepods				*C. finmarchicus*				*C. helgolandicus*			
		Training		Test		Training		Test		Training		Test	
Block	n	RMSE	R^2	RMSE	R^2	RMSE	R^2	RMSE	R^2	RMSE	R^2	RMSE	R^2
1	2038	1.07	0.92	2.67	0.26					0.68	0.93	1.43	0.22
2	4432	1.07	0.92	2.75	0.29					0.68	0.93	1.57	0.27
3	3467	1.08	0.92	2.47	0.45	0.56	0.93	1.85	0.40	0.69	0.93	1.37	**0.08**
4	5416	1.06	0.92	**2.82**	**0.18**					0.68	0.93	1.72	0.21
5	13103	1.07	0.92	2.60	0.28	0.58	0.93	1.60	**0.04**	0.66	0.93	1.95	**0.12**
6	8436	1.07	0.92	2.54	0.39	0.52	0.93	2.00	0.19	0.67	0.93	1.81	0.26
7	10627	1.07	0.92	**2.72**	**0.08**					0.65	0.93	1.95	**0.04**
8	5504	1.08	0.92	2.46	**0.23**	0.54	0.93	2.02	0.26	0.67	0.93	1.94	0.15
Mean		1.07	0.92	2.63	0.27	0.55	0.93	1.87	0.22	0.67	0.93	1.72	0.17

Table S 2: Temporal cross-validation. Root-mean square error (RMSE; log_{10} kJ.m^{-3}) and Pearson's correlation (R^2) for model trained excluding one period at a time.

	Small				*C. finmarchicus*				*C. helgolandicus*			
	Training		Test		Training		Test		Training		Test	
Block	RMSE	R^2	RMSE	R^2	RMSE	R^2	RMSE	R^2	RMSE	R^2	RMSE	R^2
1998-2005	1.04	0.92	2.63	0.30	0.58	0.93	1.42	0.25	0.67	0.93	1.79	0.25
2006-2012	1.08	0.92	2.51	0.35	0.59	0.93	1.39	0.25	0.67	0.93	1.79	0.25
2013-2019	1.08	0.92	2.53	0.33	0.53	0.93	1.80	0.24	0.67	0.93	1.80	0.22
Mean	1.07	0.92	2.56	0.33	0.56	0.93	1.53	0.25	0.67	0.93	1.79	0.24

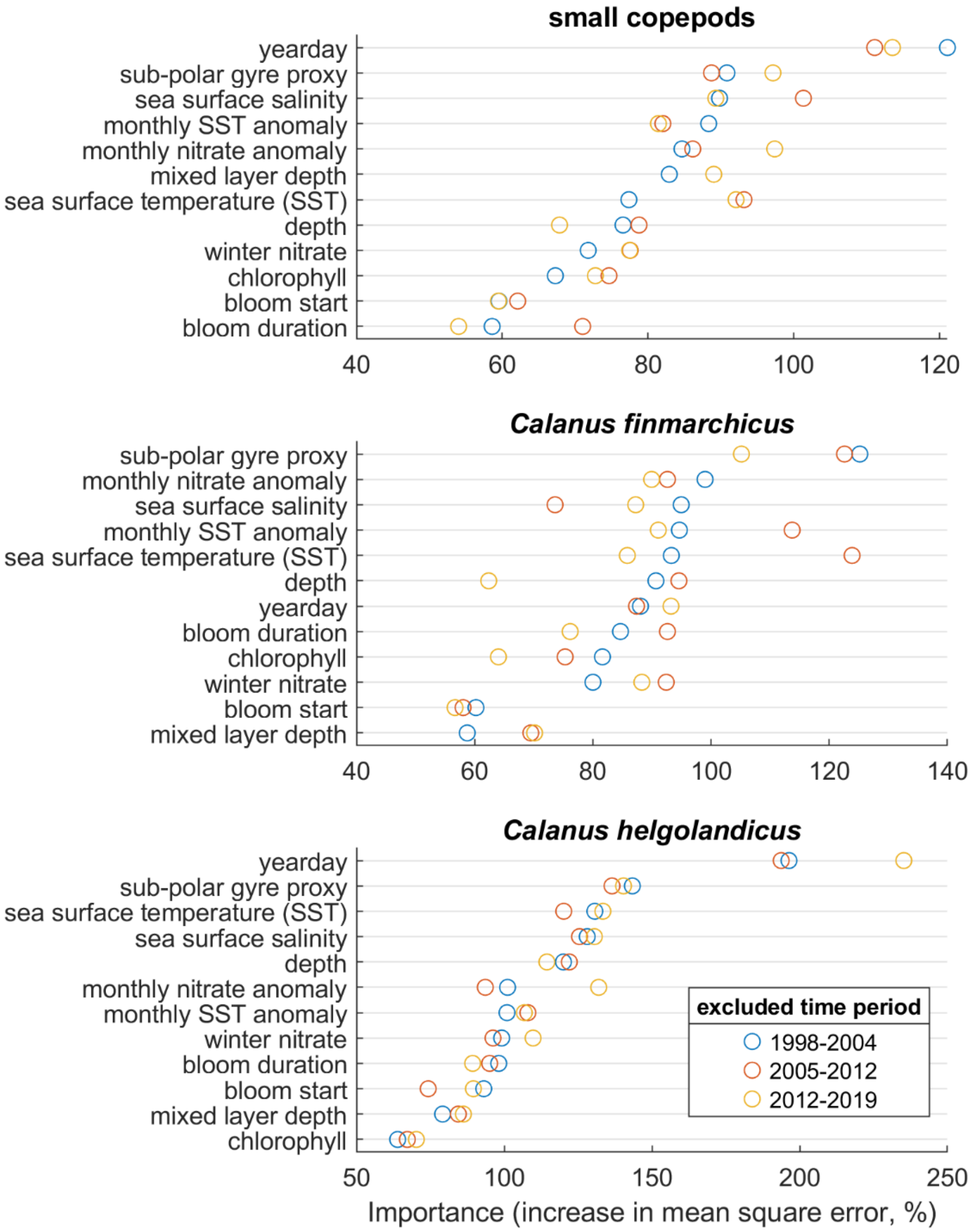

Figure S 2: Predictor variable importance ranking by cross-validation time block.

Bias corrected projected SST and SSS

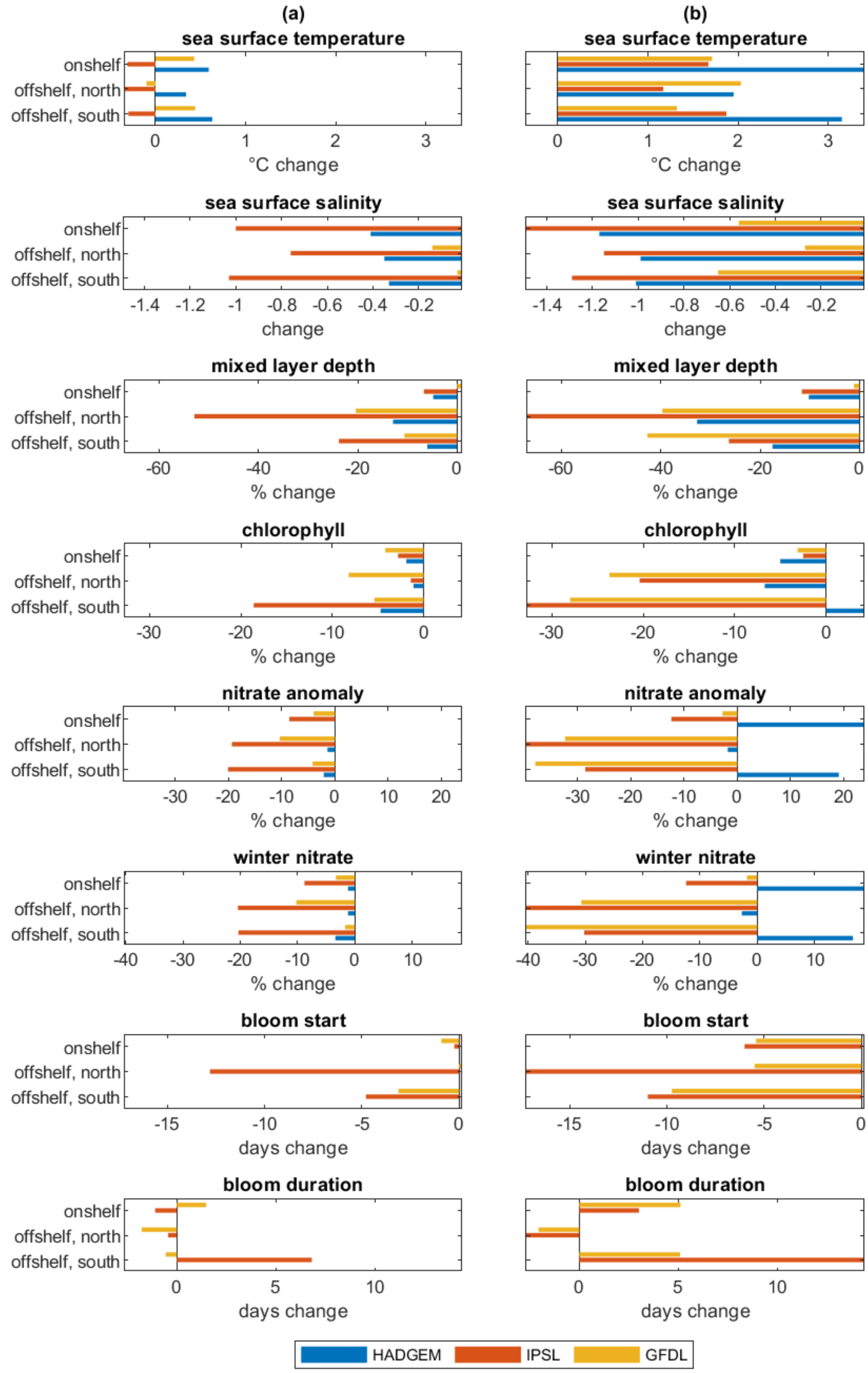

Figure S 3: Projected annual mean Delta change from recent past (2010s and 2020s) to (a) near future (2040-2050) and (b) far future (2090-2100) by predictor variable, climate projection model and region. HADGEM, IPSL and GFDL are the regional climate projection models, each using the high emissions RCP8.5 scenario but with varying model structures and global forcing data – see Table 1 of the main text for further details.

Regional zooplankton projections

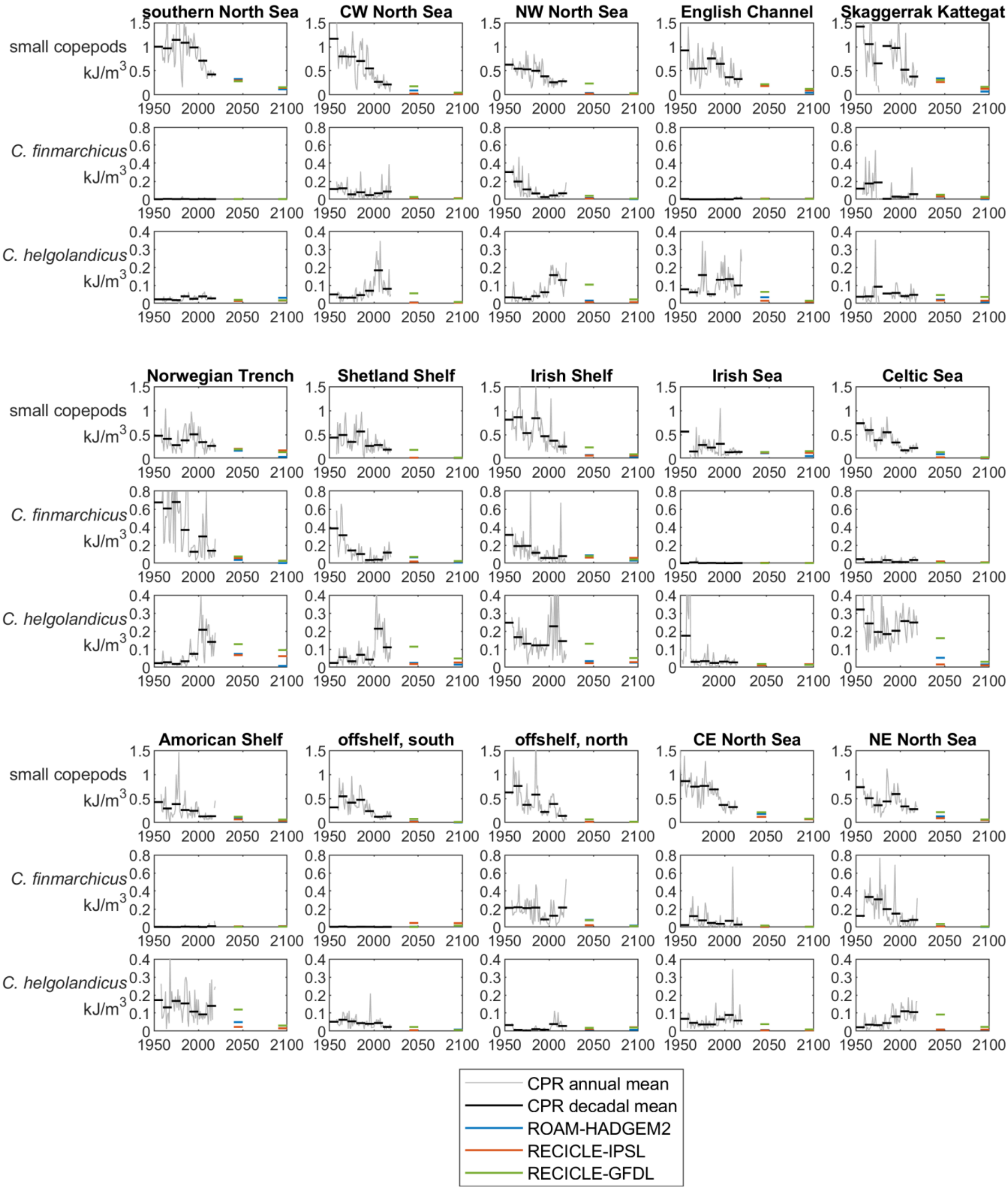

Figure S 4: Observed mean annual and decadal Continuous Plankton Recorder (CPR) zooplankton energy and modelled projected zooplankton energy by Northwest European Shelf region. ROAM-HADGEM2, RECICLE-IPSL and RECICLE-GFDL are the three regionally downscaled physical-biogeochemical ocean model projections that have been used to drive the zooplankton projection model.

Zooplankton seasonality projections

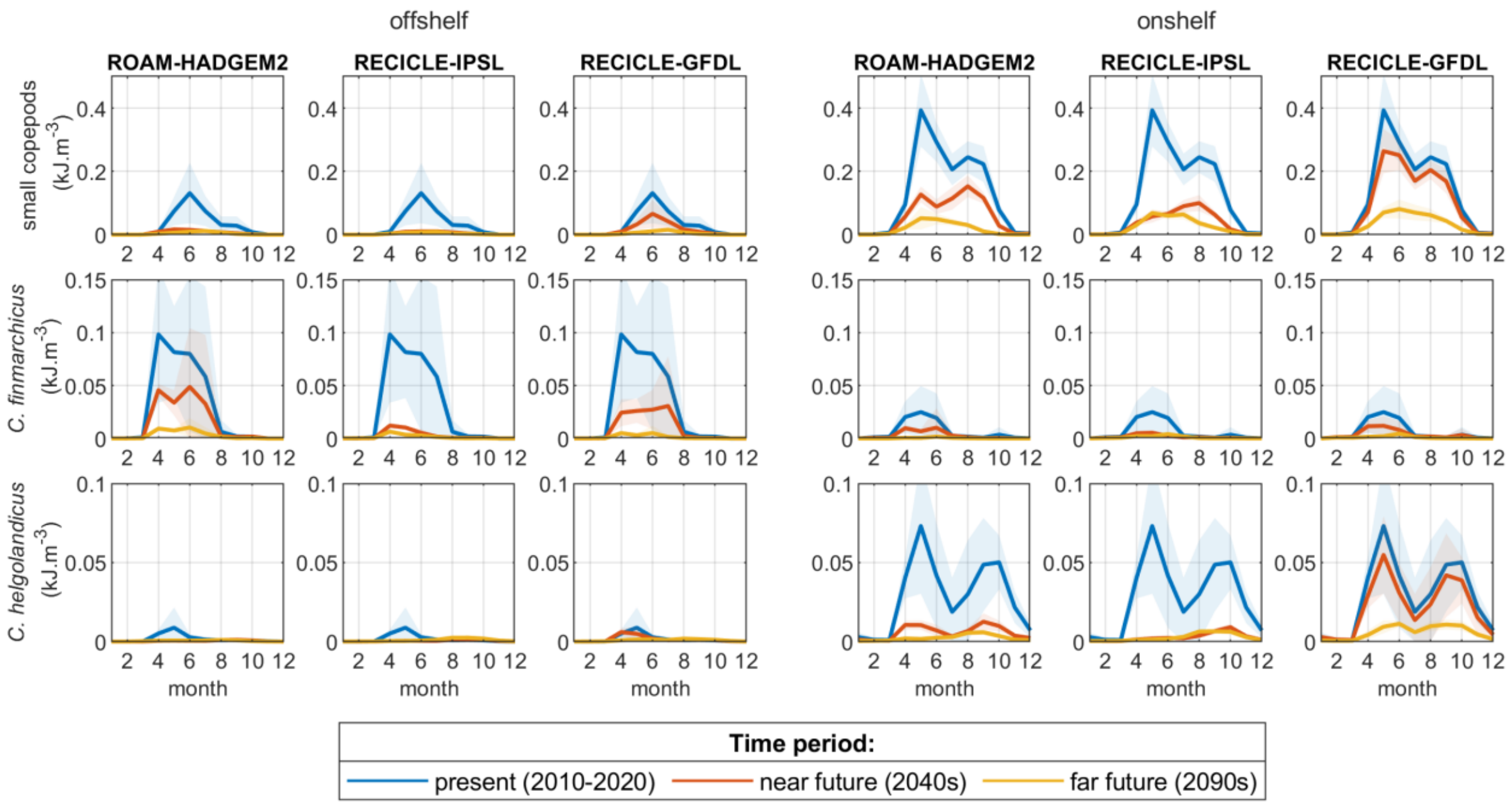

Figure S 5: Mean and standard deviation of the modelled seasonal cycles by zooplankton species, time period and climate projection model for shelf (water depth < 200 m) and off-shelf areas of the Northwest European Shelf.

Mapped zooplankton projections

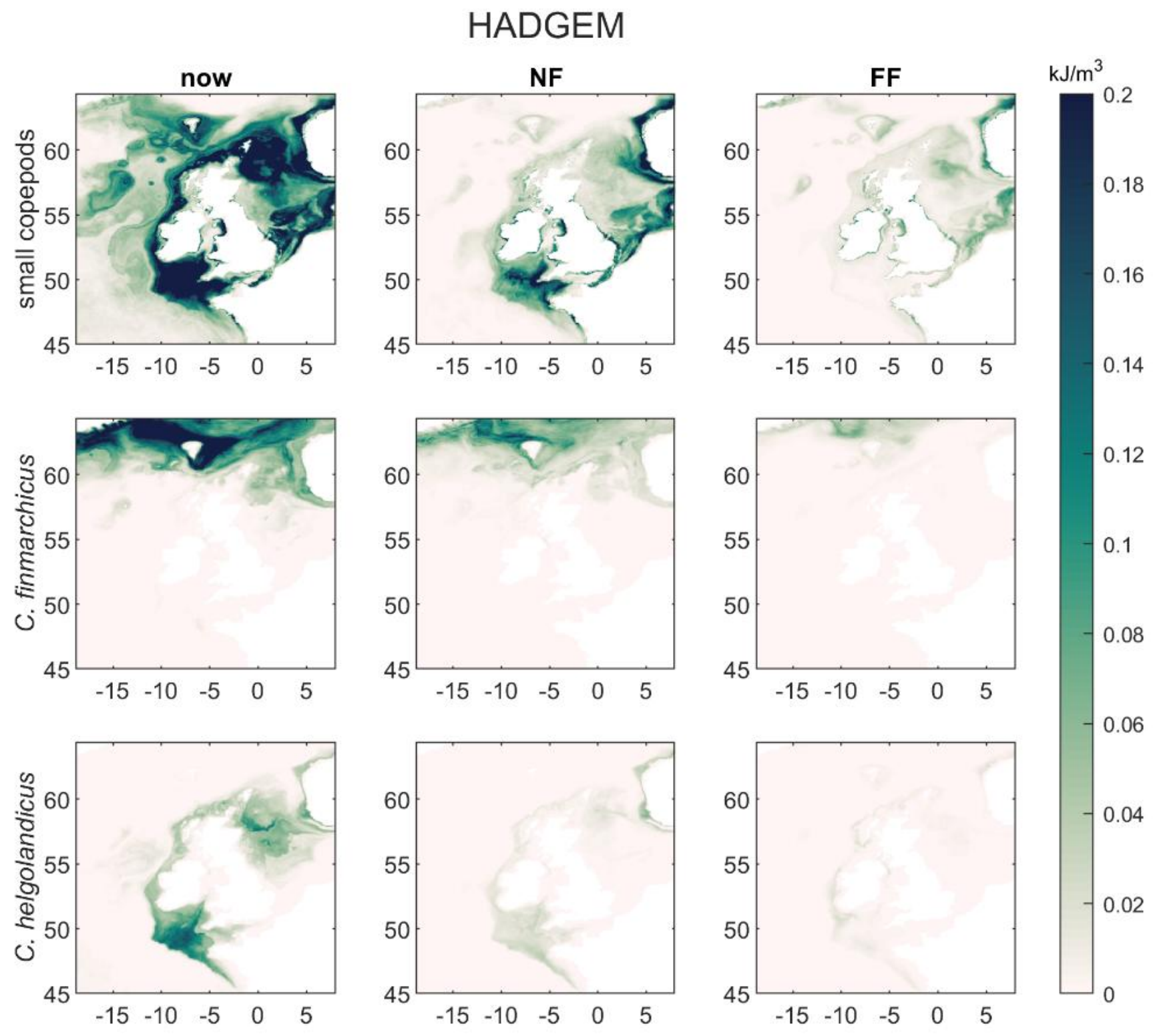
HADGEM
now
NF
FF
small copepods
C. finmarchicus
C. helgolandicus
kJ/m3
0.2
0.18
0.16
0.14
0.12
0.1
0.08
0.06
0.04
0.02
0
45
50
55
60
-15
-10
-5
0
5

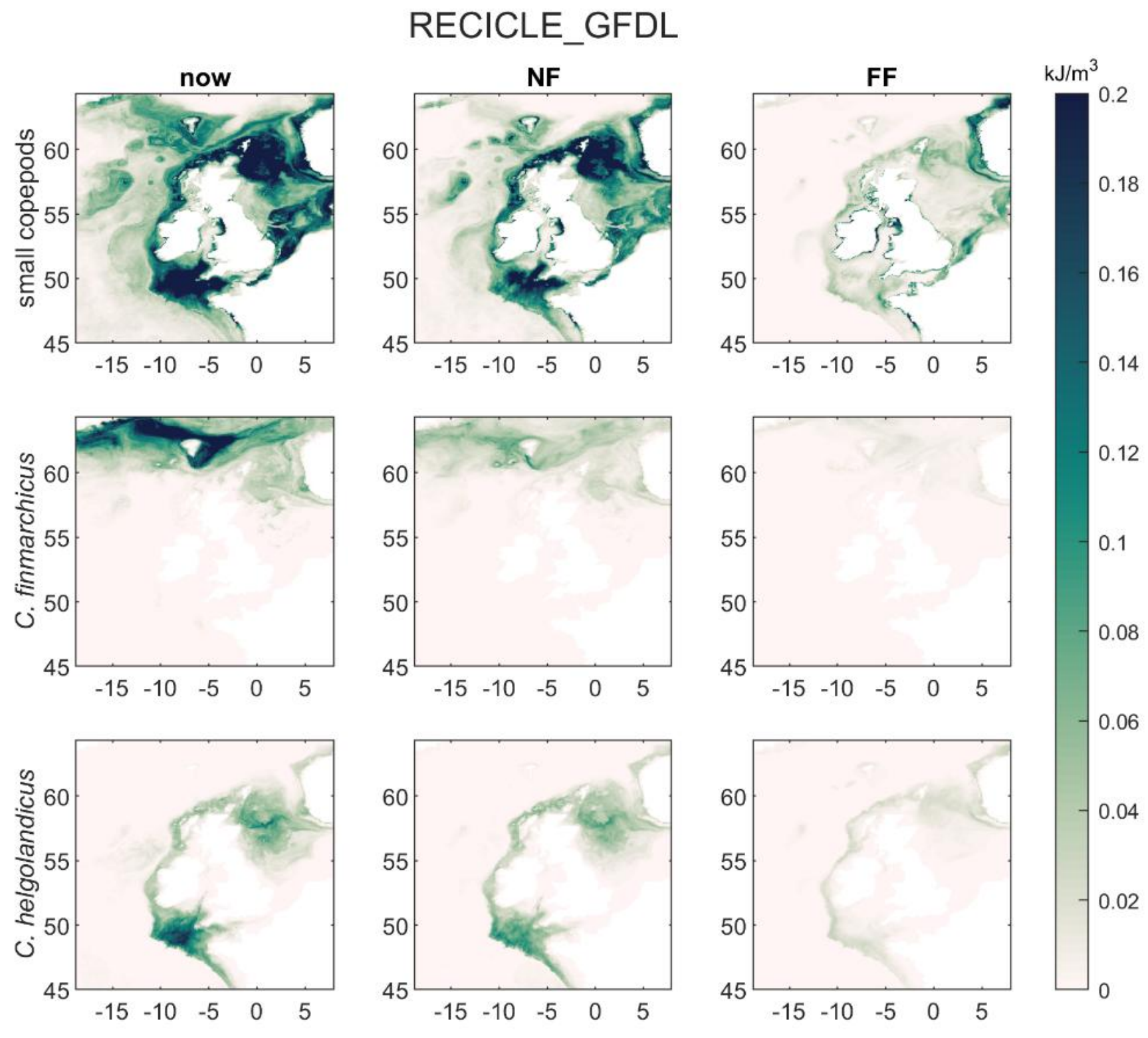
RECICLE_GFDL
now
NF
FF
small copepods
C. finmarchicus
C. helgolandicus
kJ/m3
0.2
0.18
0.16
0.14
0.12
0.1
0.08
0.06
0.04
0.02
0
45
50
55
60
-15
-10
-5
0
5

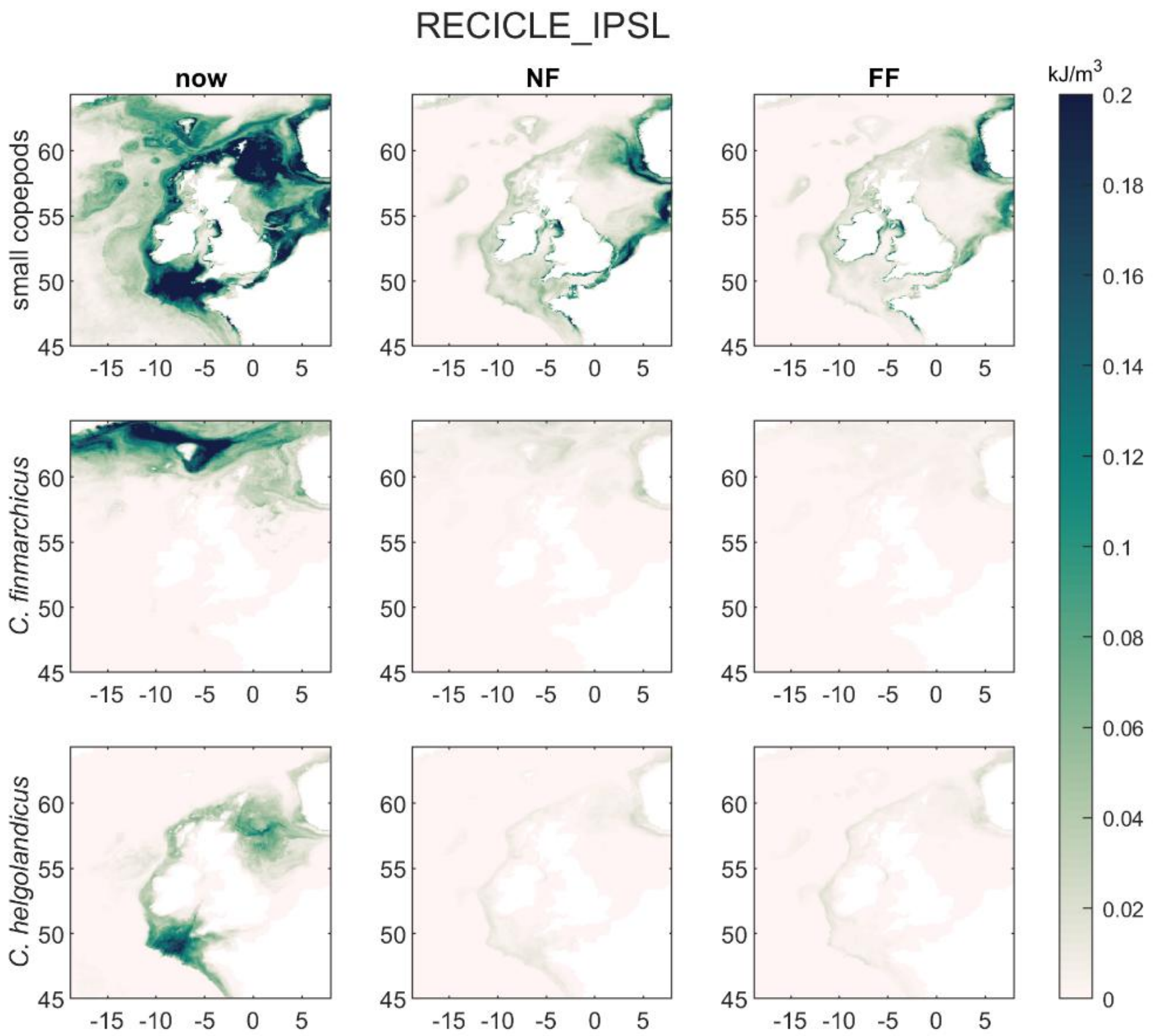

Figure S 6: Projected zooplankton energy for each climate model and zooplankton species.

Petrik et al. (2022) global zooplankton climate projections on the Northwest European Shelf

Petrik et al. (2022) assessed the skill of an Coupled Model Intercomparison Project Phase 6 (CMIP6) Earth System Models (ESMs) in representing mesozooplankton and found a wide ensemble spread in projected mean global change in mesoplankton from 1965-2014 to 2051-2100 under SSP5-8.5. As part of the present study, we wished to compare our zooplankton projections for the Northwest European Shelf with their results for the same area. The gridded global percentage change in mesozooplankton for each simulation was kindly provided by C. M. Petrik, from which we calculated an area-weighted mean over grid-cells within the Northwest European Shelf domain. The results are shown in Table S3.

Table S 3: Percent change in mean mesozooplankton biomass on the Northwest European Shelf from 1965-2014 to 2051-2100 simulated by CMIP6 ESMs under SSP5-8. See Petrik et al. (2022) for details of the simulations. Calculated here as a weighted mean from global results provided by Petrik. Results given to 2 s.f. for whole domain and shelf (<400 m water depth) and off shelf (>=400 m water depth) regions.

	Percentage change in mesozooplankton		
**CMIP6 Earth System Model**	**Whole domain**	**Shelf**	**Off shelf**
CMCC	-37	-28	-43
CNRM	-6.5	-3.1	-7.9
GFDL	-10	-11	-8.1
IPSL	-16	-12	-21
UKESM	-64	-77	-76

Mapping novel predictor space

Figure S7 shows the areas in the near and far future where environmental predictor variables are outside the bounds of the training data set. This was calculated globally, i.e. by checking at each grid-cell whether the projected variable was within the range of the training data over the whole model domain.

By the near future, the main areas with variables projected to exceed previous bounds are the Bay of Biscay (SST), the Norwegian Trench (salinity and SST anomaly), inshore areas and the eastern North Sea (SST anomaly), and the central and northern North Sea (chlorophyll and nitrate anomaly). GFDL additionally shows areas of increased stratification off the continental shelf. By the far future, most of the domain is outside the training data because of the large increase in SST anomaly compared with the 1998-2023 climatology.

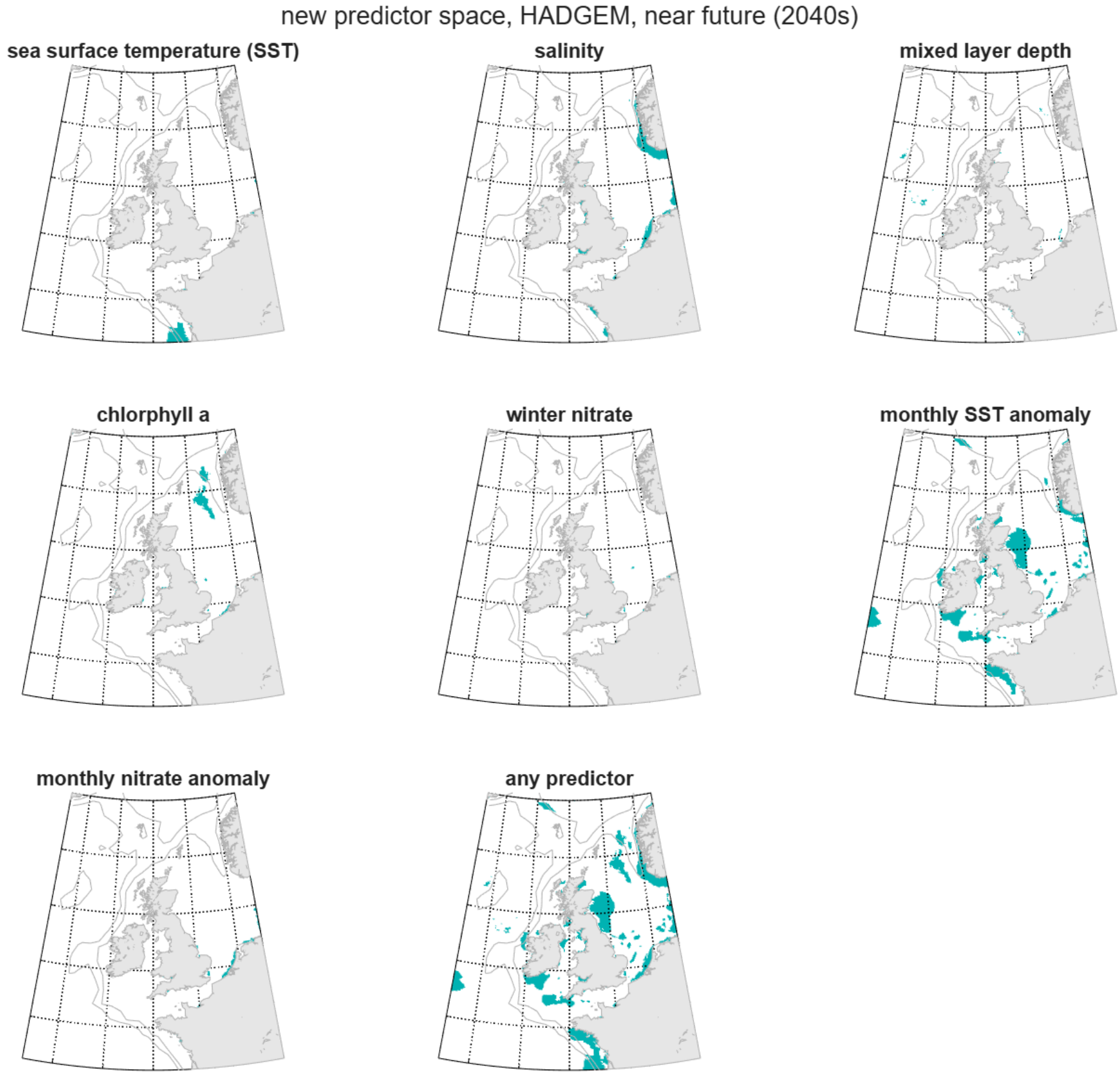
new predictor space, HADGEM, near future (2040s)
sea surface temperature (SST)
salinity
mixed layer depth
chlorphyll a
winter nitrate
monthly SST anomaly
monthly nitrate anomaly
any predictor

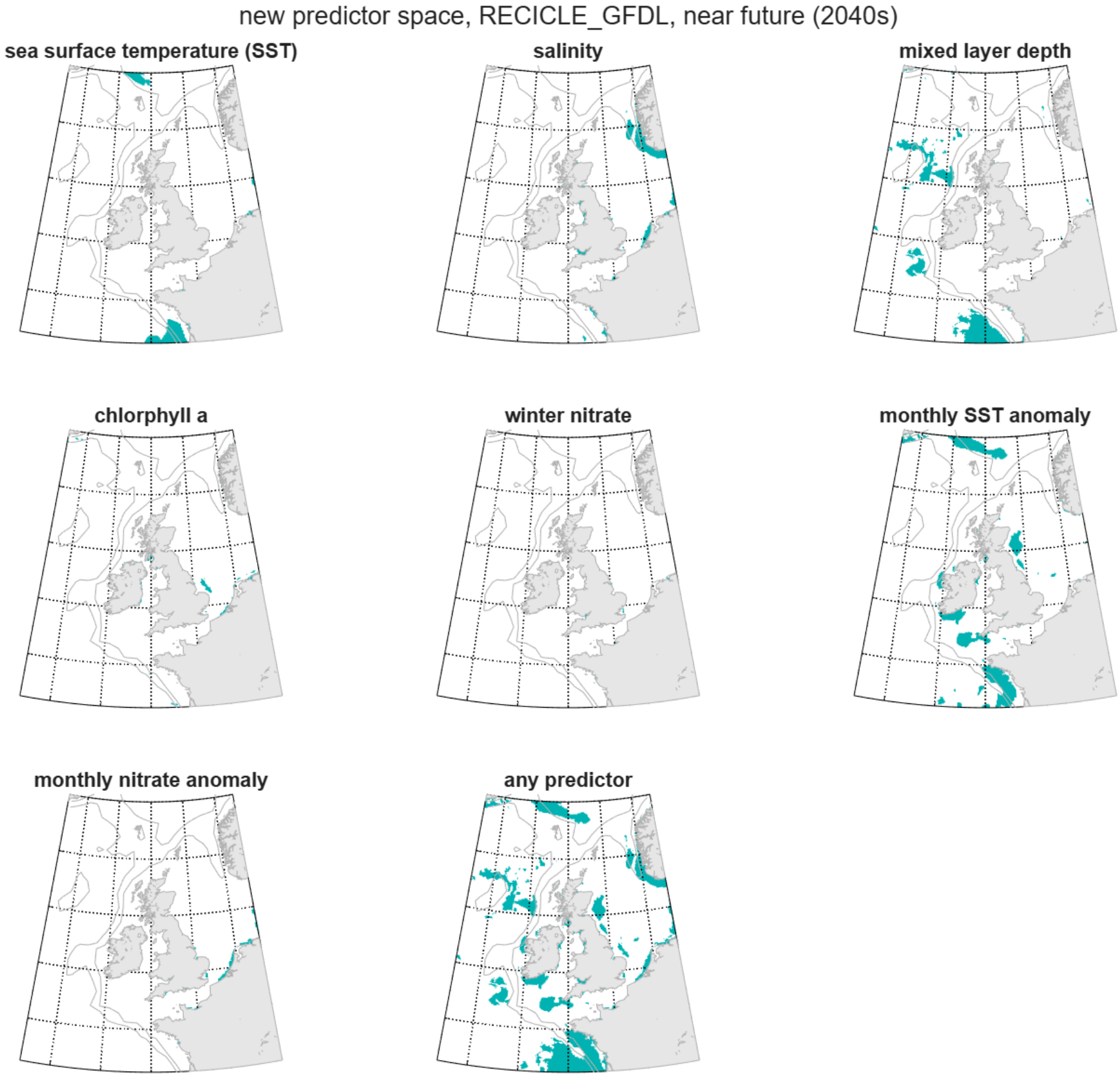
new predictor space, RECICLE_GFDL, near future (2040s)
sea surface temperature (SST)
salinity
mixed layer depth
chlorphyll a
winter nitrate
monthly SST anomaly
monthly nitrate anomaly
any predictor

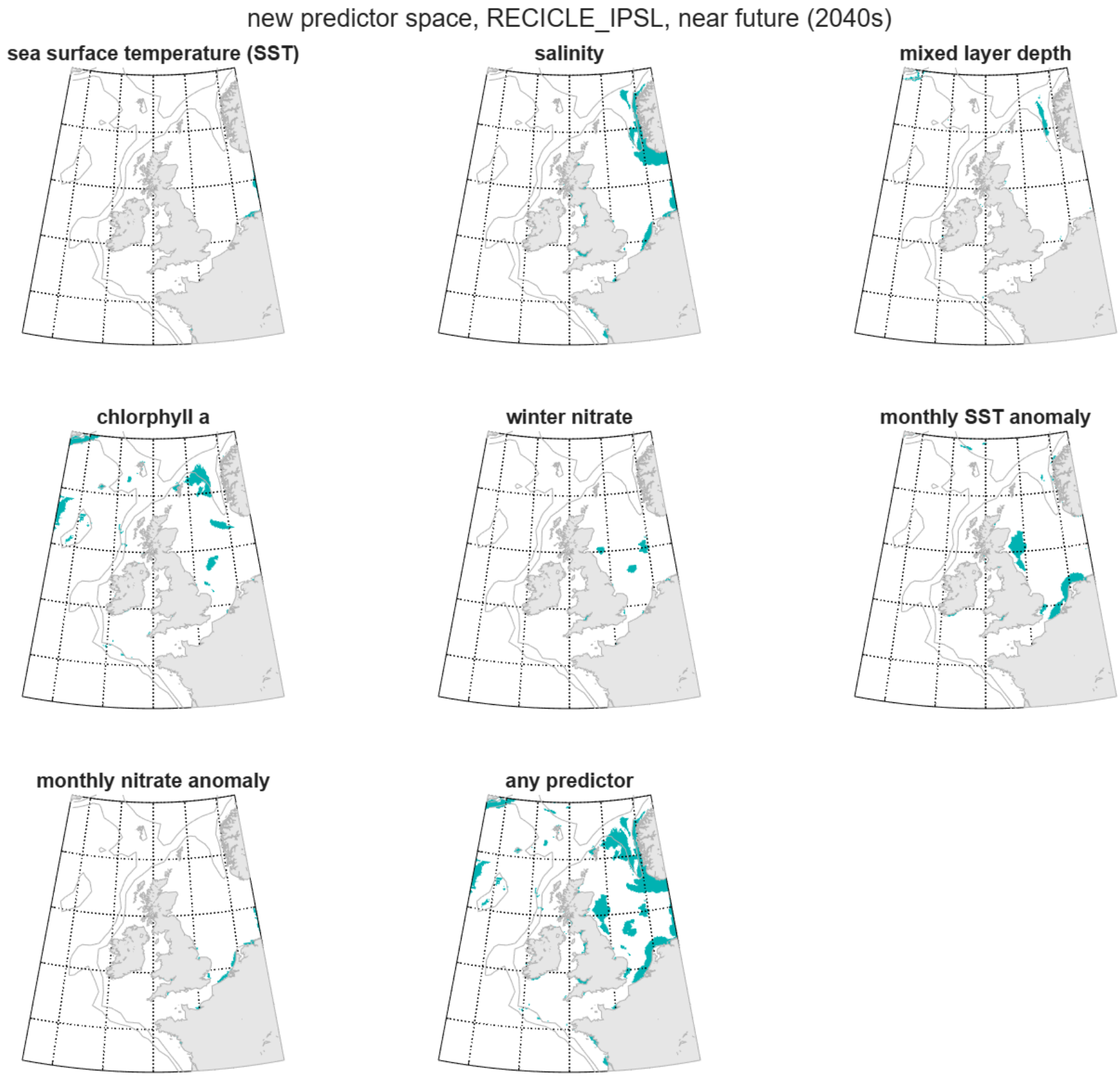
new predictor space, RECICLE_IPSL, near future (2040s)
sea surface temperature (SST)
salinity
mixed layer depth
chlorphyll a
winter nitrate
monthly SST anomaly
monthly nitrate anomaly
any predictor

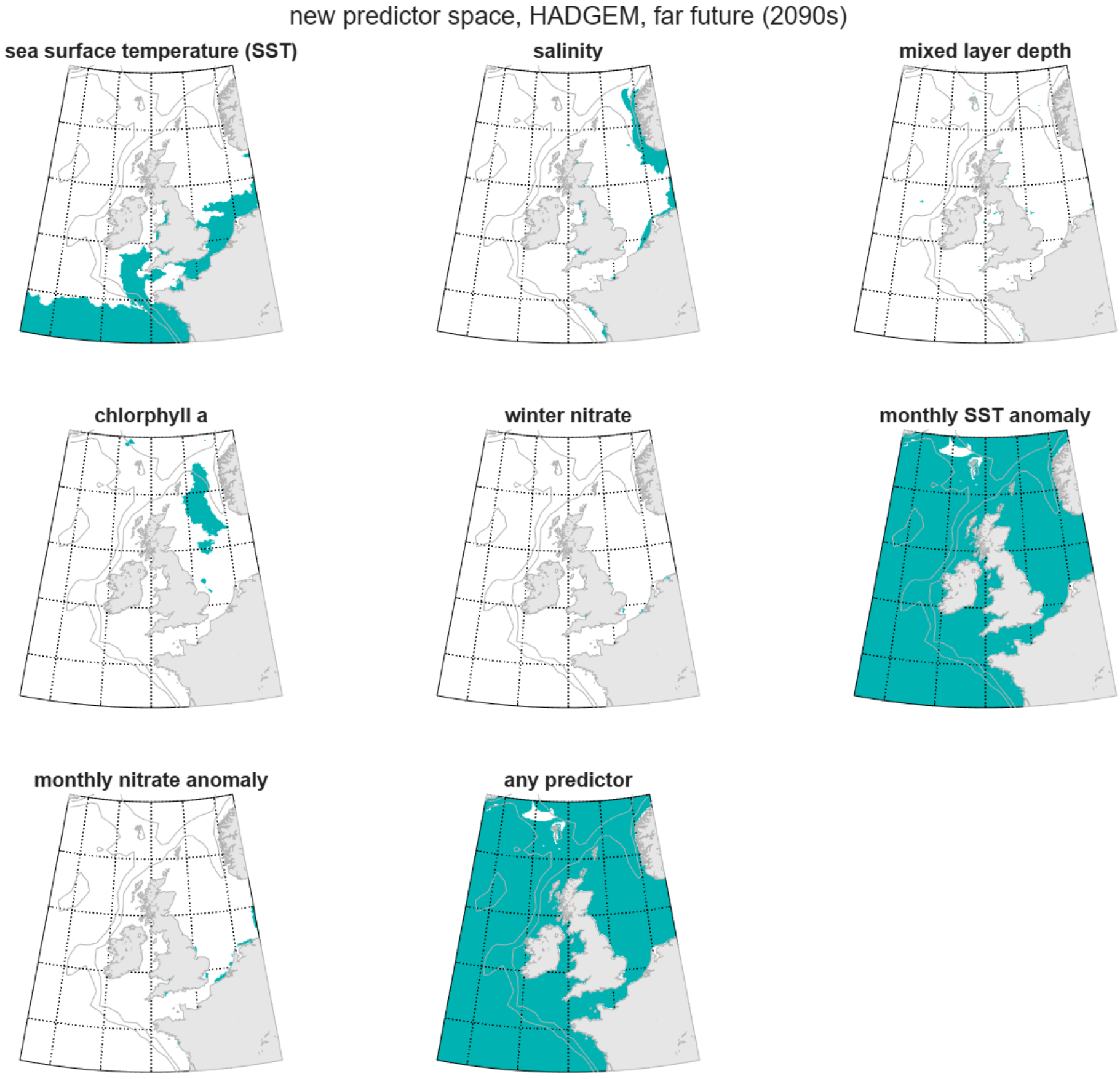
new predictor space, HADGEM, far future (2090s)
sea surface temperature (SST)
salinity
mixed layer depth
chlorphyll a
winter nitrate
monthly SST anomaly
monthly nitrate anomaly
any predictor

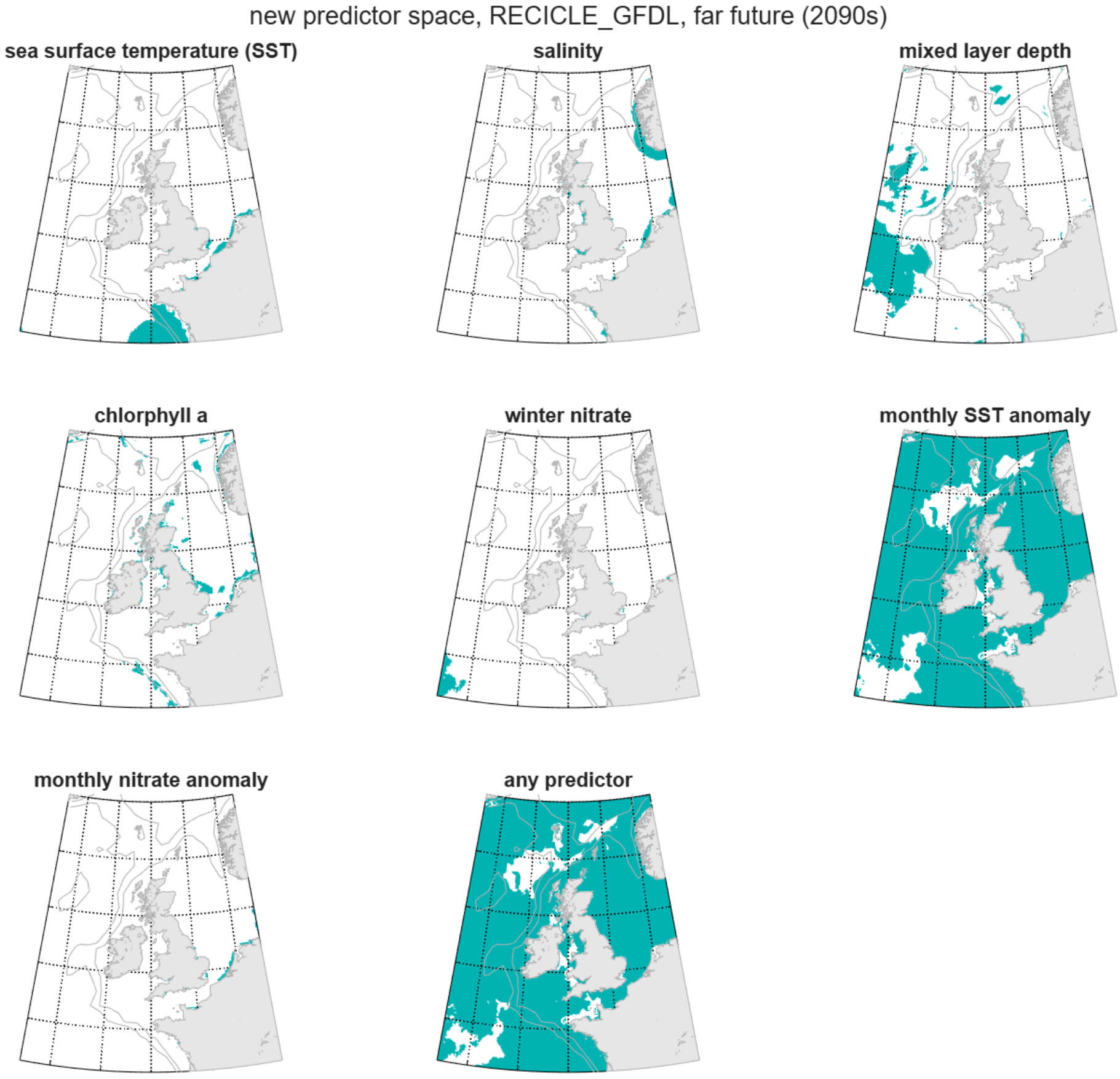
new predictor space, RECICLE_GFDL, far future (2090s)
sea surface temperature (SST)
salinity
mixed layer depth
chlorphyll a
winter nitrate
monthly SST anomaly
monthly nitrate anomaly
any predictor

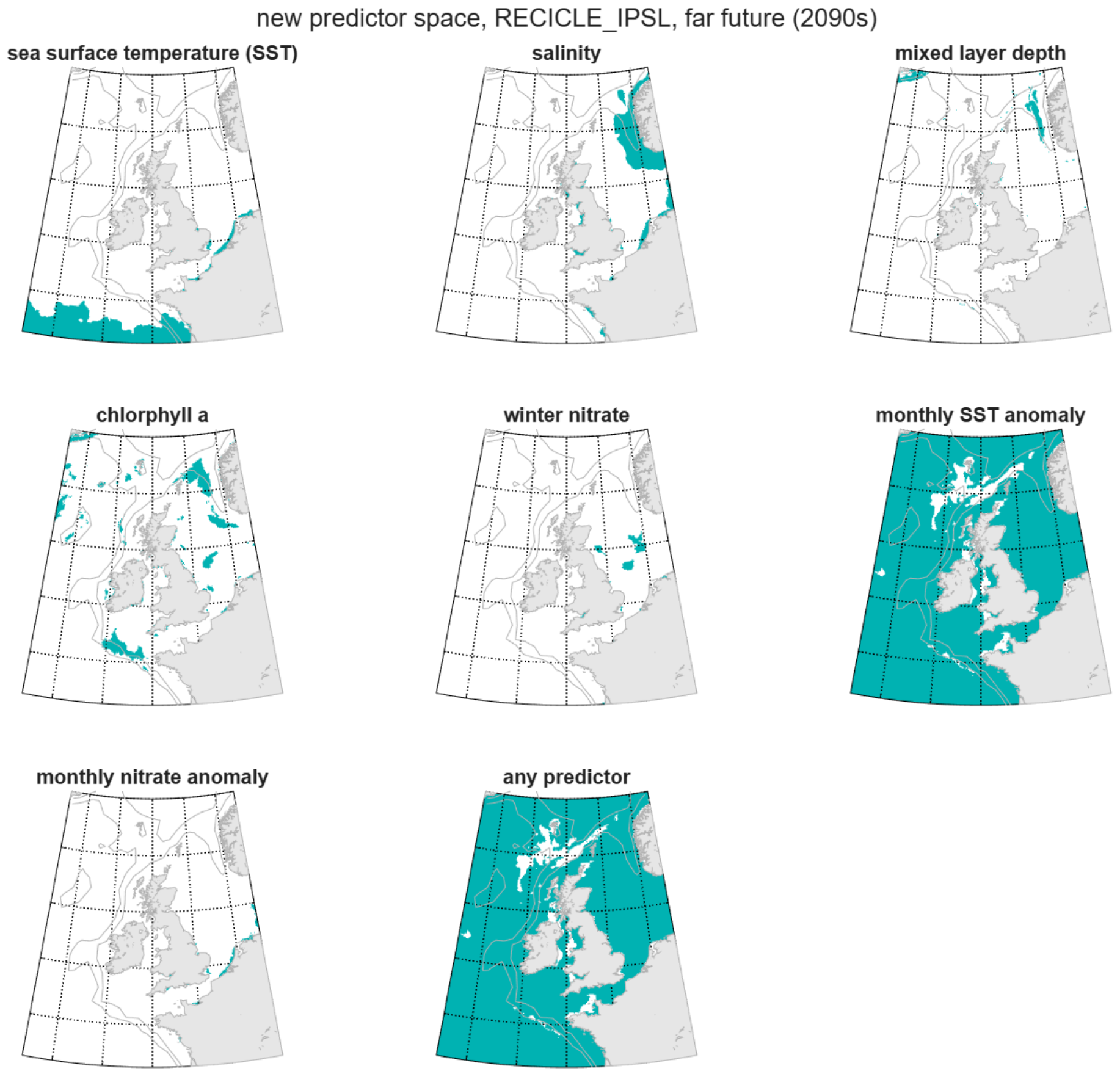

Figure S 7: Areas of the model domain in near future (2040s) and far future (2090s) where predictor variables move outside the bounds of training data for each climate projection model.
